# Synthesis, Characterization, and In Vitro Evaluation of New Amino‐Alcohol Quinolines as Promising Antimycobacterial Agents

**DOI:** 10.1002/cmdc.70543

**Published:** 2026-10-22

**Authors:** Elise Si Ahmed Charrier, François Peltier, Jean‐François Raimbert, Marie Perret, Claire Andréjak, Alexandra Dassonville‐Klimpt, Pascal Sonnet

**Affiliations:** ^1^ AGents Infectieux Résistance et chimiothérapie UR4294 UFR de Pharmacie Université de Picardie Jules Verne Amiens France; ^2^ Département de Bactériologie CHU Amiens‐Picardie Amiens France; ^3^ Service de Pneumologie CHU Amiens‐Picardie Amiens France

**Keywords:** amino‐alcohol quinolines, asymmetric synthesis, in vitro evaluation, nontuberculous mycobacteria, physicochemical properties

## Abstract

Nontuberculous mycobacteria (NTM) mainly *Mycobacterium avium* complex (MAC), are emerging pathogens responsible for pulmonary infections. Therefore, the effectiveness of the gold standard treatment is compromised by the emergence of intrinsic and acquired resistance of MAC. In this context, seventeen amino‐alcohol quinolines as mefloquine analogs were developed as part of this work. Studies on the physicochemical properties, cytotoxicity and activity of these quinoline‐based compounds, in relation to their structures, highlighted the important role of the trifluoromethyl and piperazine moieties. Thus, (*R*)‐**4c** was identified as the most promising anti‐MAC compound with the highest selectivity index value of 5.8. It was tested, in two‐drug combinations, using a checkerboard assays with the standard antibiotics (clarithromycin, rifampicin and ethambutol), demonstrating an additive effect with each antibiotic of this gold standard treatment. These results confirm the potential of amino‐alcohol quinolines as alternative of current anti‐NTM agents.

## Graphical abstract

Nontuberculous mycobacteria (NTM) mainly *Mycobacterium avium* complex (MAC), are emerging pathogens responsible for pulmonary infections. The efficacy of the standard treatment is therefore compromised by the emergence of intrinsic and acquired resistance in MAC. In this context, (*R*)‐**4c**, an amino‐alcohol quinoline, was identified as the most promising anti‐MAC compound, thereby confirming the potential of this class of molecules.

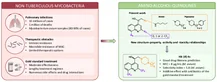

**Abbreviations**
ATB: antibiotic
CC_50_
: median cytotoxic concentration
CFZ: clofazimine
CLR: clarithromycin
ee: enantiomeric excess
EMB: ethambutol
FICI: fractional inhibitory concentration index
HBA: hydrogen bond acceptor
HBD: hydrogen bond donor
MAC: *Mycobacterium avium* complex
MIC: minimal inhibitory concentration
MOX: moxifloxacin
MQ: mefloquine
MTT: 3‐(4,5‐dimethylthiazol‐2‐yl)‐2,5‐diphenyltetrazolium bromide
MW: molecular weight
NA: not applicable
ND: not determined
NTM: nontuberculous mycobacteria
PC: physicochemical
PK: pharmacokinetic
RIF: rifampicin
SAR: structure–activity relationships
SI: selectivity index
SPR: structure–property relationships
STR: structure–toxicity relationships

## 1 Introduction

Pulmonary infections caused by mycobacteria affect several million people worldwide each year. In 2024, more than 10 million cases and over 1 million deaths attributable to *Mycobacterium tuberculosis* were reported [1]. Although this species is the most widely recognized, nontuberculous mycobacteria (NTM) are increasingly emerging in certain regions of the world such as Northern Europe. In 80%–90% of cases, these pathogens infect the lungs of immunocompromised individuals and patients with chronic respiratory diseases [2]. The majority of pulmonary infections are caused by species of the *Mycobacterium avium* complex (MAC).

The gold standard treatment for MAC infections includes a macrolide (clarithromycin (CLR) or azithromycin), a rifamycin (rifampicin (RIF) or rifabutin), and ethambutol (EMB) [3]. However, treatment efficacy, estimated at 52%–66%, is limited by the thick and poorly permeable cell wall of MAC, as well as by the emergence of resistance to macrolides, which constitute the main class of antibiotics used in therapy [4, 5, 6]. In addition, the treatment regimen is lengthy and associated with numerous adverse effects [7].

Currently, various therapeutic approaches are being developed to improve treatment efficacy and reduce side effects, while also limiting the risk of resistance development [8]. For the treatment of MAC infections, clinical studies are underway to optimize the CLR/RIF/EMB triple therapy or to replace some of these antibiotics (example switch RIB by clofazimine (CFZ)) in order to enhance both tolerability and effectiveness [7, 9, 10]. New formulations are also being explored, particularly the development of amikacin liposomal suspensions or CFZ inhaled formulations, which are currently in clinical trials [11, 12, 13, 14, 15, 16, 17]. These delivery systems offer the advantage of increasing antibiotic concentrations within pulmonary tissues, thereby improving efficacy while reducing systemic side effects. Another strategy involves the development of new anti‐MAC agents with novel mechanisms of action, such as epetraborole which inhibits the leucyl‐tRNA synthetase [18, 19].

Additionally, the repurposing of antitubercular compounds such as streptomycin, linezolid, or bedaquiline is also being investigated for activity against MAC [7, 10, 20, 21]. Bedaquiline is currently undergoing phases II/III clinical trials to evaluate its efficacy and safety against *M. avium*, combined with CLR and EMB (NCT04630145) [22]. Mefloquine (MQ), an antimalarial drug available as a racemic mixture of (+) and (−)‐*erythro* enantiomers, has also been studied as a repurposed antimycobacterial drug (Figure 1) [24, 25, 26, 27]. This amino‐alcohol quinoline exhibited moderate minimal inhibitory concentration (MIC) values of 4–16 μg/mL against most NTM including both MAC macrolide‐susceptible and resistant strains [23, 25]. However, due to its lipophilic nature (*c*logP = 3.5) and its long half‐life estimated at 16.4 days, MQ is able to accumulate intracellularly, making it bactericidal against MAC in vivo [23, 25, 27, 28]. This effect is enhanced when MQ is combined with EMB and moxifloxacin (MOX) [27]. Bermudez et al. demonstrated that MQ/EMB/MOX combination decreased colony‐forming unit counts per mL in both the liver and the spleen compared with MQ alone. However, triple antibiotic therapy was not more effective than MQ/MOX dual therapies [26].

**FIGURE 1:**
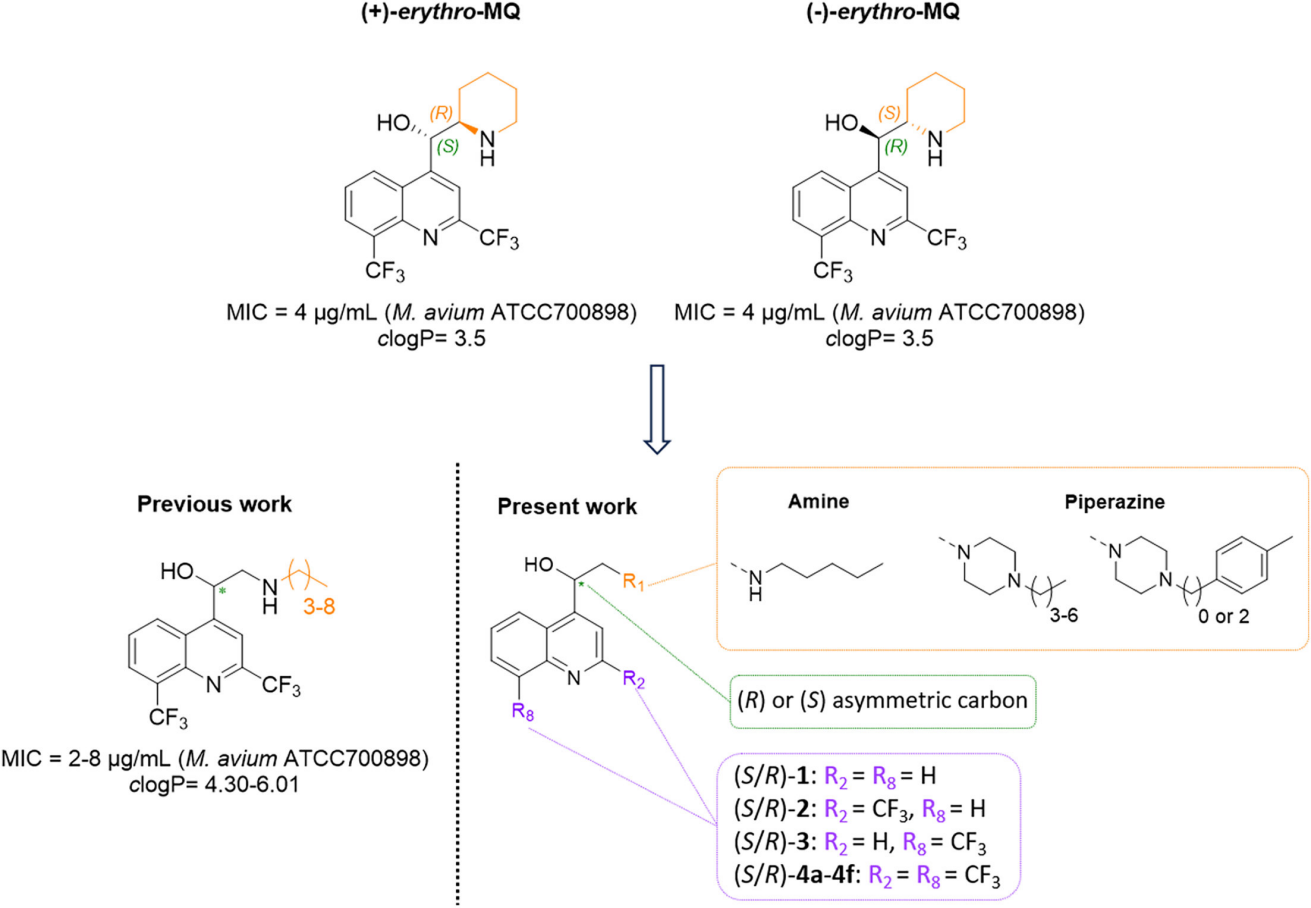
Previous work inspired by MQ and the design of new generation of amino‐alcohol quinolines [23].

This synergistic effect may be explained by adjuvant properties of MQ. Although its antibacterial mechanism of action is not yet fully elucidated, data have shown that MQ is able to interact with lipid components of the mycobacterial cell wall, thereby disrupting its integrity and facilitating the entry of the associated antibiotic into mycobacteria. Other hypotheses suggest that it may interfere with (myco)bacterial efflux pumps and mycobacterial ATP synthase, the target of bedaquiline [29, 30, 31, 32].

Interestingly, MQ stereoisomers seem to have different behaviors in vitro and in vivo. Bermudez et al. previously identified the (+)‐*erythro*‐MQ as the most effective enantiomer against MAC infection in mice [33]. Recently, the most in vivo MAC efficacy of (+)‐MQ was reaffirmed when combined with CLR and EMB [34]. This difference may be explained by stereospecific interactions with the MQ target and different pharmacokinetic (PK) profiles of MQ enantiomers [35, 36]. Thus, MQ is an attractive lead compound for pharmacomodulation in the treatment of MAC infections, as it acts on multiple targets that is represent an important advantage in limiting the emergence of resistance, and it can be combined with other antimycobacterial agents. However, MQ exhibits anti‐MAC activity that could be enhanced and causes side effects, primarily neuropsychiatric effects which can be attributed to the (−)‐MQ [37]. So, our team is motivated to find new MQ analogs enantiomerically pure that demonstrate greater anti‐MAC efficacy without significant toxicity.

Previously, we reported the anti‐MAC activity of a series of enantiomeric MQ analogs bearing aliphatic chains of varying lengths (C_4_–C_9_) (Figure 1) [23]. Generally, these compounds were as active as *erythro*‐MQ enantiomers, with MIC values typically ranging from 2 to 8 µg/mL against MAC. The first structure–activity relationships (SAR) highlighted that neither the side‐chain length nor the absolute configuration of the stereogenic center influenced the antimycobacterial activity. Motivated by these encouraging results, we describe here new chemical modifications to the quinoline pharmacophore. Given the importance of piperazine and trifluoromethyl substituents in the design of bioactive molecules, these groups were introduced and their number varied in order to assess their impact on antimycobacterial activity and PK properties [38, 39, 40, 41, 42, 43].

To evaluate the influence of the trifluoromethyl group, we synthesized three pairs of enantiomers (**1**–**3**), corresponding to MQ analogs bearing a moderate‐length aliphatic chain (pentyl chain), incorporating zero or one trifluoromethyl group at the C_2_ and/or C_8_ positions of the quinoline scaffold. Due to its amphiphilic character, which facilitates modulation of the overall lipophilicity of compounds, a piperazine moiety was introduced between the quinoline scaffold and the aliphatic chains (**4a–** **d**). This moiety was either optionally functionalized with a toluene group (**4e)**, or directly linked to a toluene group (**4f**). As the differences in activity and toxicity between the two enantiomers of MQ have been demonstrated, particularly in vivo, compounds described herein are enantiomerically pure to enable evaluation of the effect of absolute configuration on antimycobacterial activity and cytotoxicity.

Here, we report the asymmetric synthesis of 17 amino‐alcohol quinolines, and the determination of their in silico and/or experimental physicochemical (PC) properties, together with their in silico PK profiles. Their in vitro antimycobacterial activity against MAC strains, as well as their cytotoxicity, are also discussed. Furthermore, to mitigate the risk of resistance development, the hit compound identified in these studies was subsequently evaluated in two‐drug combinations with antibiotics from the gold standard treatment using checkerboard assays.

## 2 Results and Discussion

### 2.1 Chemistry

The synthesis of amino‐alcohol quinolines (*S*/*R*)‐**1** **–4a** **–f** required the coupling of the corresponding amino moiety, pentan‐1‐amine or piperazines **5**–**9**, to the related epoxide including quinoline either unsubstituted (**10**) or bearing one (**11** and **12**) or two trifluoromethyl groups (**13**) (Scheme 1). The amino substituents are commercial (pentan‐1‐amine or 1‐(*p*‐tolyl)piperazine) or prepared (*N*‐alkylpiperazines **5**–**9**). Epoxides (*S*/*R*)‐**10**–**13** were synthesized from the corresponding diols (*S*/*R*)‐**14**–**17**, which were themselves obtained from the related 4‐hydroxyquinoline **18**–**21** [44]. Of these 4‐hydroxyquinolines **18**‐**21**, compounds **18**, **19**, and **21** are commercially available; consequently, only 4‐hydroxy‐8‐(trifluoromethyl)quinoline **20** was synthesized [45, 46, 47].

**SCHEME 1:**
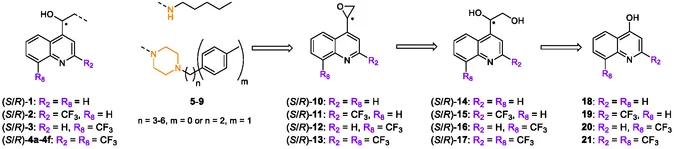
Retrosynthetic route for final amino‐alcohol quinolines (*S*/*R*)‐**1**‐**4a–f**.

#### 2.1.1 Synthesis of 4‐Hydroxy‐8‐(Trifluoromethyl)quinoline 20

4‐Hydroxy‐8‐(trifluoromethyl)quinoline **20** was synthesized in four steps (Scheme 2) [45, 48]. First, 2‐(trifluoromethyl)aniline **22** was reacted with diethyl ethoxymethylene malonate **23** to afford the diethyl 2‐(((2‐(trifluoromethyl)phenyl)amino)methylene)malonate **24** in a moderate yield of 41%. Next, ethyl 4‐hydroxy‐8‐(trifluoromethyl)quinoline‐3‐carboxylate **25** was obtained in 70% yield via cyclization in diphenylester at 250 °C for 4 h. Subsequent saponification afforded 4‐hydroxy‐8‐(trifluoromethyl)quinoline‐3‐carboxylic acid **26** in 91% yield. Finally, the key intermediate 4‐hydroxy‐8‐(trifluoromethyl)quinoline **20** was obtained in 65% yield via decarboxylation at the C_3_ position in diphenylester at 250 °C.

**SCHEME 2:**
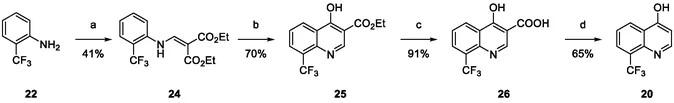
Synthetic route for 8‐(trifluoromethyl)‐quinolin‐4‐ol **20**. Reagents and conditions: (a) diethyl ethoxymethylenemalonate **23**, NaOH, 110 °C, 24 h; (b) diphenylether, 250 °C, 6 h; (c) NaOH (1N), 100 °C, 19 h; (d) diphenylether, 250 °C, 2 h.

#### 2.1.2 Synthesis of Epoxides 10–13

Epoxides **10**–**13**, required for the synthesis of the final amino‐alcohol quinolines, were obtained through a four‐step sequence (Scheme 3) [44]. First, 4‐hydroxyquinolines **18**–**21** were reacted with POBr_3_ to afford 4‐bromoquinolines **27**–**30** in yields ranging from 56% to 96% (Table 1). Suzuki–Miyaura coupling reaction was carried out to obtain 4‐vinylquinolines **31**–**34** in 57% to 77% yield. Then, **31**–**34** were converted to (*S*) or (*R*) diols by an asymmetric Sharpless dihydroxylation using AD‐mix *α* or AD‐mix *β*, respectively. The diols (*S*/*R*)‐**14**–**17** were obtained in yields ranging from 83% to 99% and an enantiomeric excess (ee) higher than or equal to 91%. A one‐pot SN_2_ ring closure with retention of the configuration was carried out to give the corresponding enantiopure epoxides (*S*/*R*)‐**11** and (*S*/*R*)‐**13** with a yield ranging from 51% to 54% and an ee of 80% or higher. However, for the unsubstituted epoxides (*S*/*R*)‐**10** and mono‐substituted at C_8_ position **12a**–**b**, the one‐pot epoxidation result in partially (50%) and complete racemization, respectively. This loss of stereoselectivity could be explained by the destabilization of the bromide intermediate due to the absence of the electron‐withdrawing ─CF_3_ group at the C_2_ position of the quinoline, promoting racemization.

**SCHEME 3:**
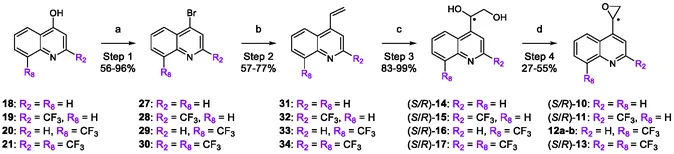
Synthetic route for epoxides **10**–**13**. Reagents and conditions: (a) POBr_3_, 150 °C, 18 h. (b) C_2_H_3_BF_3_K, Cs_2_CO_3_, PdCl_2_(dppf)·CH_2_Cl_2_, THF/H_2_O (9/1, v/v), 90 °C, 16 h. (c) AD‐mix *α* or *β*, K_2_[OsO_2_(OH)_4_], *tert*‐BuOH/H_2_O (1/1, v/v), 0 °C–25 °C, 16 h. (d) (i) CH_3_C(OCH_3_)_3_, PTSA·H_2_O, CH_2_Cl_2_, 25 °C, 5 h; (ii) TMSBr, CH_2_Cl_2_, 25 °C, 16 h; (iii) K_2_CO_3_, CH_3_OH, 25 °C, 4 h.

**TABLE 1:** Yields, enantiomeric excesses, and optical rotation of intermediate compounds.

Compound	***R***_**2**_	***R***_**8**_	**Yield, %** a	**ee, %** b	${\left[\alpha \right]}_{D}^{T}$ c
**Step 1: Bromination**					
**27**	H	H	96	NA	NA
**28**	CF_3_	H	56		
**29**	H	CF_3_	96		
**30**	CF_3_	CF_3_	95		
**Step 2: Suzuki–Miyaura coupling**					
**31**	H	H	67	NA	NA
**32**	CF_3_	H	62		
**33**	H	CF_3_	57		
**34**	CF_3_	CF_3_	77		
**Step 3: Asymmetric Sharpless dihydroxylation**					
(*S*)‐**14**	H	H	94	94	${\left[\alpha \right]}_{D}^{21}$= +79
(*R*)‐**14**			95	95	${\left[\alpha \right]}_{D}^{21}$ = −70
(*S*)‐**15**	CF_3_	H	83	93	${\left[\alpha \right]}_{D}^{21}$ = +20
(*R*)‐**15**			94	93	${\left[\alpha \right]}_{D}^{21}$ = −26
(*S*)‐**16**	H	CF_3_	99	91	${\left[\alpha \right]}_{D}^{21}$ = +58
(*R*)‐**16**			99	96	${\left[\alpha \right]}_{D}^{21}$ = −50
(*S*)‐**17**	CF_3_	CF_3_	99	91	${\left[\alpha \right]}_{D}^{20}$ = +31
(*R*)‐**17**			95	91	${\left[\alpha \right]}_{D}^{20}$ = −37
**Step 4: Epoxidation**					
(*S*)‐**10**	H	H	55	50	${\left[\alpha \right]}_{D}^{21}$ = +18
(*R*)‐**10**			27	55	${\left[\alpha \right]}_{D}^{21}$ = −23
(*S*)‐**11**	CF_3_	H	51	86	${\left[\alpha \right]}_{D}^{21}$ = +57
(*R*)‐**11**			54	80	${\left[\alpha \right]}_{D}^{21}$ = −57
**12a** d	H	CF_3_	29	NA	NA
**12b** d			55	NA	NA
(*S*)‐**13**	CF_3_	CF_3_	51	91	${\left[\alpha \right]}_{D}^{21}$ = +46
(*R*)‐**13**			54	93	${\left[\alpha \right]}_{D}^{21}$ = −49

Abbreviation: NA, not applicable.

a Isolated yield in the final step.

b Enantiomeric excess determined by chiral HPLC; see conditions in the Experimental Section.

c See optical rotation, concentration, solvent, and temperature conditions in the Experimental Section.

d NA, ee < 10%, ${\left[\alpha \right]}_{D}^{21}$ ≈ 0.

The final step consisted of nucleophilic substitution of the corresponding epoxides **10**–**13** with pentan‐1‐amine to form amino‐alcohol quinolines **1**–**3** or with synthesized *N*‐alkylpiperazines **5**–**9** (see conditions in the Experimental Section) to form amino‐alcohol quinolines **4a**–**4e** or with commercial 1‐(p‐tolyl)piperazine to form amino‐alcohol quinolines **4f**. The final step allowed to obtain **1–** **4a–** **f** with yield ranging from 17% to 80%. Thus, amino‐alcohol quinolines **2** and **4a**–**f** were afforded with overall yields ranging from 6% to 31% over five or seven steps and with ee ranging from 84% to 97%, except for **1** and **3a**, **b** compounds which were obtained with partial (50%) or complete racemization with overall yields of 1%–6% (Table 2).

**TABLE 2:** Yields, enantiomeric excesses, and optical rotation of final amino‐alcohol quinolines.

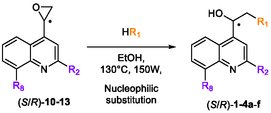					
**Compound**	**R**_**1**_	**Yield, %** a	**Overall yield, %** b	**ee, %** c	${\left[\alpha \right]}_{D}^{T}$ d
R_2_= R_8_= H					
(*S*)‐**1** (*R*)‐**1**	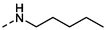	25 17^e^	6 3^e^	NA NA	NA NA
R_2_= CF_3_, R_8_= H					
(*S*)‐**2** (*R*)‐**2**	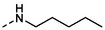	63 56	10 8	84 93	${\left[\alpha \right]}_{D}^{22}$ = +133 ${\left[\alpha \right]}_{D}^{22}$ = −125
R_2 _= H, R_8 _= CF_3_					
**3a** f **3b** f	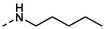	69 55	2 1	NA NA	NA NA
R_2 _= R_8 _= CF_3_					
(*S*)‐**4a** (*R*)‐**4a**	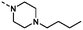	50 40	18 9	91 89	${\left[\alpha \right]}_{D}^{21}$ = +57 ${\left[\alpha \right]}_{D}^{21}$ = −63
(*S*)‐**4b** (*R*)‐**4b**	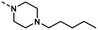	80 67	18 6	87 86	${\left[\alpha \right]}_{D}^{21}$ = +69 ${\left[\alpha \right]}_{D}^{21}$ = −48
(*S*)‐**4c** (*R*)‐**4c**	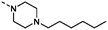	59 63	18 29	97 93	${\left[\alpha \right]}_{D}^{21}$ = +79 ${\left[\alpha \right]}_{D}^{21}$ = −75
(*S*)‐**4d** (*R*)‐**4d**	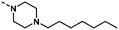	63 75	13 15	85 85	${\left[\alpha \right]}_{D}^{21}$ = +59 ${\left[\alpha \right]}_{D}^{21}$ = −66
(*S*)‐**4e** (*R*)‐**4e**	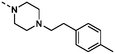	44 77	14 11	91 89	${\left[\alpha \right]}_{D}^{21}$ = +93 ${\left[\alpha \right]}_{D}^{21}$ = −98
(*S*)‐**4f** (*R*)‐**4f**	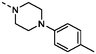	79 69	31 30	92 94	${\left[\alpha \right]}_{D}^{21}$ = +53 ${\left[\alpha \right]}_{D}^{21}$ = −51

Abbreviation: NA, not applicable.

a Isolated yield in the final step.

b Calculated yield over all steps.

c Enantiomeric excess determined by chiral HPLC; see conditions in the Experimental Section.

d See optical rotation, concentration, solvent, and temperature conditions in the Experimental Section.

e Yield corrected for 70% purity.

f NA, ee < 10%, ${\left[\alpha \right]}_{D}^{21}$ ≈ 0.

### 2.2 Drug‐Likeness Prediction

Selected structural, PC, and PK properties of the amino‐alcohol quinolines, as racemic mixtures, were measured in silico using the Schrödinger software. Particular parameters, including pKa and partition coefficients logP and logD, were determined experimentally using the Sirius T3 Pion instrument. The calculated and experimental values were compared with those of the racemic MQ and with standard ranges defined by Lipinski’s rule of five, commonly used to predict oral absorption [49].

Overall, the synthesized amino‐alcohol quinolines meet the recommended thresholds for hydrogen bond acceptor (HBA), hydrogen bond donor (HBD), and molecular weight (MW) with the exception of enantiomers **4e** for MW (Table 3). For unsubstituted or mono‐substituted quinolines (**1**–**3**), logP values are below 5. For quinolines with two trifluoromethyl substituents (**4a**–**f**), experimental logP values are often higher than 5, except for **4a** and **4f**. However, logD values measured at physiological pH remain below 4, except for **4d**. Thus, the synthesized amino‐alcohol quinolines, similarly to MQ, display structural and PC properties compatible with favorable oral absorption.

**TABLE 3:** Physicochemical descriptors, pKa, and solubility values of final amino‐alcohol quinolines.

Compound	MW, g/mol	**HBA** a	**HBD** a	pKa	logP	logD (pH = 7.4)	**S, mol/L** a In silico data
				Experimental data			
R_2 _= R_8 _= H							
**1**	258.17	3	2	5.83^a^, ^b^ 9.13^a^	2.90^a^	0.19^d^	6.92 × 10^−4^
R_2 _= CF_3_, R_8 _= H							
**2**	326.36	3	2	8.82^c^	3.41^c^	NA^c^	2.64 × 10^−4^
R_2 _= H, R_8 _= CF_3_							
**3**	326.36	3	2	2.56^c^ 8.86^c^	3.92^c^	1.64^c^	7.38 × 10^−4^
R_2 _= R_8 _= CF_3_							
**4a**	449.19	4	1	2.57^c^ 8.19^c^	4.68^c^	1.71^c^	3.09 × 10^−5^
**4b**	463.21	4	1	2.77^c^ 8.09^c^	5.07^c^	2.23^c^	6.02 × 10^−5^
**4c**	477.22	4	1	3.06^c^ 8.06^c^	6.42^c^	3.62^c^	4.68 × 10^−5^
**4d**	491.24	4	1	2.66^c^ 7.85^c^	9.14^c^	6.72^c^	2.29 × 10^−5^
**4e**	511.21	4	1	3.17^c^ 7.83^c^	5.39^c^	1.88^c^	3.24 × 10^−6^
**4f**	483.17	4	1	5.52^c^	3.93^c^	2.38^c^	6.63 × 10^−7^
**References**							
Racemic MQ	414.77	2	3	1.43^a^ 4.87^a^	3.87^a^	2.13^d^	3.16 × 10^−5^

a Calculated by the software Schrödinger.

b pKa <1 did not measured by Sirius and are not reported in this table.

c Measured by the Sirius T3 Pion; see conditions in the Experimental Section.

d Calculated by the software Pallas.

The pKa values reflect the ionization state of the molecules, which governs their ability to cross biological membranes. The quinoline nitrogen is essentially nonbasic (pKa = 0.43–0.54), due to the presence of the electron‐withdrawing ─CF_3_ substituent at position C_2_, for compounds **2** and **4a**–**f**. These pKa values did not measured by the Sirius. The quinoline nitrogen is basic in the other compounds, **1** and **3**, with pka values of 5.83 and 2.56, respectively. Compounds **1**, **2**, and **3** contain a secondary aliphatic amine with a pKa between 8.82 and 9.13, which is predominantly protonated at pH 7.4.

The piperazine compounds **4a**–**f** possess two basic functions (i) the nitrogen substituted by the aliphatic chain with pKa between 7.83 and 8.19 depending on the length of the aliphatic chain, which is mainly protonated at pH 7.4, and (ii) the second nitrogen, with an experimental pKa of approximately 3, significantly lower compared to the first one due to the electron‐withdrawing effect of the ammonium ion formed during the first protonation, which is unprotonated at physiological pH. For enantiomers **4f**, the piperazine nitrogen in the alpha position of the secondary alcohol exhibits a higher predicted pKa than the nitrogen bearing the aryl group, as the latter contributes less to the molecule’s basic character. The majority of the amino‐alcohol quinolines display solubility values within the range recommended by Schrödinger software, except **4f** (S ≤ 6.63 × 10^−7 ^mol/L). Although the alkylamine and *N*‐alkylpiperazine chains differ, the aqueous solubility of quinolines **4a**–**f** disubstituted with ─CF_3_ groups is lower than that of monosubstituted and unsubstituted compounds **1**–**3**.

Thus, the presence of trifluoromethyl substituent increases lipophilicity, which generally enhances membrane permeability but reduces aqueous solubility. The incorporation of a piperazine moiety improves aqueous solubility through increased ionization and hydrophilicity. Hence, the combination of both ─CF_3_ and piperazine on the quinoline core leads to compounds with the best lipophilicity/hydrophilicity balance, that is generally compatible with good oral bioavailability.

### 2.3 In Vitro Antimycobacterial Activity and Cytotoxicity

The anti‐NTM activity of all synthesized amino‐alcohol quinolines, except for (*R*)‐**1** (*R*/*S*: 75/25) due to its low purity, was evaluated against reference strains of *M. avium* subsp. *hominissuis* and other slow‐growing NTM such as *M. marinum*, *M. xenopi* and *M. kansasii*, as well as rapid‐growing NTM such as the rough (*R*) and smooth (*S*) phenotypes of *M. abscessus* subsp*. abscessus* (Table 4). CLR, RIF, and EMB included in the gold standard treatment, racemic MQ, and its two enantiomers (+)‐MQ and (−)‐MQ were used as references. Most of the amino‐alcohol quinolines were active against both slow‐growing and rapid‐growing NTM, except for **1** which was completely inactive against all strains (MIC > 128 µg/mL). Compounds **3a,** **b** were inactive against the two phenotypes of *M abscessus* R and S, while compound **4f** was inactive against both *M avium* and *M abscessus*. The active compounds were often more effective against *M. kansasii* (MIC ≤ 2–8 µg/mL) compared to other NTM, as the references. They were often less active (MIC ≤ 2–64 µg/mL) than CLR, RIF and EMB (MIC = 0.25–4 µg/mL) against other slow‐growing NTM strains and as active against rapid‐growing strains (MIC = 16–‍64 µg/mL). In most cases, these compounds were as active as racemic MQ and its two enantiomers against slow‐growing NTM strains (MIC ≤ 2 to 16 µg/mL vs. MIC ≤ 2–4 µg/mL) and more active against rapid‐growing strains (MIC = 4–64 µg/mL vs. MIC = 16–64 µg/mL). As for the two MQ enantiomers, no difference in activity was observed between the enantiomers of an amino‐alcohol quinoline pair, except for **4c** against *M. xenopi* and for **4d** and **4e** against *M. marinum* and/or *M. avium* (*e.g.*, MIC (*R*)‐**4c** = 8 µg/mL and MIC (*S*)‐**4c** = 1 µg/mL against *M. xenopi*). At equivalent aliphatic chain length (pentyl chain), enantiomers **2** with ─CF_3_ at position 2 of the quinoline were active against *M. avium* and *M. abscessus* (MIC = 8–32 µg/mL), whereas **3** with ─CF_3_ at position 8 were active against *M. avium* (MIC = 8 µg/mL) and inactive against rapid‐growing strains (MIC = 128 µg/mL). For the majority of amino‐alcohol quinolines substituted by two trifluoromethyl groups and piperazine moiety (**4a**–**f**), activity was observed against all tested reference strains (MIC ≤ 64 µg/mL), except (*S*)‐**4e** which was inactive against *M. abscessus* (MIC > 128 µg/mL) and enantiomers **4f** which were inactive against *M. avium* and both *M. abscessus* R and S (MIC > 64 µg/mL). Among compounds **4a**–**f**, those bearing a long aliphatic chain (hexyl and heptyl chains) were more active than those with a shorter chain (butyl and pentyl chains) against all NTM strains (MIC = 4–16 µg/mL for **4c** and **4d** vs. MIC = 8–64 µg/mL for **4a** and **4b**). The benefit of an alkyl chain was also highlighted with enantiomers **4e**, which were more active than the corresponding pair **4f** without an ethyl chain between the piperazine moiety and the aryl group (MIC = 4–32 µg/mL vs. MIC = 16 to >128 µg/mL). The first SAR can be established for this new generation of amino‐alcohol quinolines: (i) the stereochemistry does not significantly affect activity, (ii) the substitution of quinoline core by one ─CF_3_, particularly at position 2, or two ─CF_3_ is essential for antimycobacterial activity, (iii) substitution by a piperazine–alkyl is beneficial for activity, and (iv) a long alkyl chain contributed to improve activity compared with shorter carbon chains.

**TABLE 4:** In vitro antimycobacterial activity, cytotoxicity, and selectivity indexes of final amino‐alcohol quinolines.

Compound	**MIC, µg/mL** a						**CC**_**50 **_ **± SD, µg/mL** b	**SI** c
	*M. avium*	*M. marinum*	*M. kansasii*	*M. xenopi*	*M. abscessus* R	*M. abscessus* S	HepG2	*M. avium*
R_2 _= R_8 _= H								
(*S*)‐**1** d	>128	ND	ND	ND	>128	>128	11.0 ± 2.6	<0.1
R_2 _= CF_3,_ R_8 _= H								
(*S*)‐**2** (*R*)‐**2**	16 16	ND	ND	ND	32 16	16–32 8	3.8 ± 1.5 5.6 ± 1.6	0.2 0.3
R_2 _= H, R_8 _= CF_3_								
**3a** **3b**	8 8	ND	ND	ND	128 128	128 128	1.2 ± 0.4 2.7 ± 1.6	0.2 0.3
R_2 _= R_8 _= CF_3_								
(*S*)‐**4a** (*R*)‐**4a**	64 32	8 16	≤2 4	8 16	64 32	64 64	27.0 ± 2.8 23.3 ± 2.1	0.4 0.7
(*S*)‐**4b** (*R*)‐**4b**	8 32	8 16	≤2 ≤2	8 16	32 32	32 32	18.2 ± 6.1 32.7 ± 3.0	2.3 1.0
(*S*)‐**4c** (*R*)‐**4c**	8 8	4 8	≤2 ≤2	1 8	16 16	16 8	12.5 ± 0.3 46.0 ± 0.5	1.6 5.8
(*S*)‐**4d** (*R*)‐**4d**	4 16	≤2 8	≤2 ≤2	≤2–4 8	4 4	8 4	16.5 ± 2.9 20.1 ± 1.8	4.1 1.3
(*S*)‐**4e** (*R*)‐**4e**	8 32	4 4	≤2 4	8 4	32–64 8	>128 16	24.0 ± 0.04 43.4 ± 2.0	3.0 1.4
(*S*)‐**4f** (*R*)‐**4f**	>128 >128	32 16	≤2 8	16 16	≥128 ≥128	≥128 ≥128	18.8 ± 1.6 22.9 ± 1.2	<0.1 <0.2
**References**								
Racemic MQ	≤2	4	≤2	1	16–32	16–32	2.6 ± 0.4	≥1.3
(−)‐MQ	4	4	≤2	2	16	32	1.2 ± 0.1	0.3
(+)‐MQ	≤2	4	≤2	1	32	64	1.2 ± 0.2	≥1.7
CLR	0.5–1	0.25–0.5	≤2	≤0.125	>16	>16	32.6 ± 6.7	32.6–65.2
RIF	≤0.25–1	0.25–0.5	0.25	2	>16	>16	>58	>58
EMB	4–16	≤1–2	<2	2–4	32	64	>20.4	1.3–5.1

Abbreviation: ND, not determined.

a *M. avium* ATCC700898, *M. marinum* DSM44344, *M. kansasii* DSM44162, *M. xenopi* DSM43995, *M. abscessus* R, and S DSM44196.

b Results are mean ± standard deviation from three experiments; see conditions in the Experimental Section.

c Selectivity index = CC_50_/MIC on *M. avium* ATCC700898.

d Compound obtained from (*S*)‐10 (mixture of *S*/*R*: 75/25).

The median cytotoxic concentration (CC_50_) of the amino‐alcohol quinolines and references was determined in the human hepatic cancer cell line HepG2 (Table 4), using the MTT assay allowing the potential toxicity of metabolites to be taken into account. CC_50_ values of amino‐alcohol quinolines **1**‐**3** (CC_50_ ≤ 11.0 µg/mL) were close to those of racemic MQ, (−)‐MQ and (+)‐MQ (CC_50_ ≤ 2.6 µg/mL). However, they were more cytotoxic than CLR, RIF, and EMB by a factor greater than 5.8. In contrast, all amino‐alcohol quinolines with a piperazine moiety (**4a**–**f**) were less cytotoxic than MQ and displayed moderately cytotoxicity, similar to that observed for most reference antibiotics. Overall, amino‐alcohol quinolines substituted by two ─CF_3_ groups (**4a**–**f**) were less cytotoxic than those unsubstituted (**1**) or substituted by one ─CF_3_ group (**2** and **3**), suggesting that trifluoromethyl substituents favor higher CC_50_ values (CC_50_ = 12.5–46.0 vs. 1.2–11 µg/mL). Introduction of an ethyl chain between the piperazine and the toluene group (**4e**) reduced cytotoxicity (CC_50_ (**4e**) = 24.0–43.4 µg/mL vs. CC_50_ (**4f**) = 18.8–22.9 µg/mL). Structure–toxicity relationships (STR) can be established for compounds **1**‐**4a**‐**f**: (i) the stereochemistry slightly affects cytotoxicity with the (*R*)‐enantiomers being less toxic than the (*S*)‐enantiomers (CC_50_ (*R*) >1.2–3.7‐fold CC_50_ (*S*)), and (ii) the substitution by both piperazine and two trifluoromethyl substituents decreased cytotoxicity.

Due to the high prevalence of *M. avium* in pulmonary infections and the limited therapeutic options avaiblable, we were interested to evaluate the compound with the best balance activity/toxicity in combination with antibiotics of the gold standard treatment using checkerboard assays to highlight potential synergistic effect [34, 50]. Thus, the selectivity index (SI) of the amino‐alcohol quinolines was calculated against *M. avium* (Table 4). As stereochemistry slightly affects cytotoxicity, the SI also differ between the (*R*) and (*S*) enantiomers, with variations ranging from 1.2‐ to 3.6‐fold. Among the compounds evaluated, (*R*)‐**4c** displayed the highest SI value of 5.8 and was selected for checkerboard assays.

The anti‐NTM activity of the selected amino‐alcohol quinolines (*R*)‐**4a** and (*R*)‐**4c**–**f** was evaluated against clinical isolates of *M. avium*, *M. xenopi*, *M. abscessus* sp. *bolletii*, and sp. *massiliense* obtained from patients at the Amiens‐Picardie University Hospital (Table 5). Racemic MQ, CLR, and RIF were used as references. The selected final compounds exhibited a similar anti‐NTM activity profile to that observed against the reference strains. Although these results confirmed the interest of our amino‐alcohol quinolines and especially against *M. abscessus* sp. *bolletii*, a macrolide resistant strain, additional evaluations against other clinical strains, particularly those resistant to macrolides should be evaluated.

**TABLE 5:** In vitro antimycobacterial activity on clinical strains of selected final amino‐alcohol quinolines.

Compound	MIC, µg/mL			
	***M. avium*** a	***M. xenopi*** b	***M. abscessus* sp. *bolletii*** c	***M. abscessus* sp. *massiliense*** d
R_2 _= R_8 _= CF_3_				
(*R*)‐**4a**	32	16	64	64
(*R*)‐**4c**	16–32	8	8–16	8
(*R*)‐**4d**	16	8	4	4
(*R*)‐**4e**	32	8	8	8
(*R*)‐**4f**	>128	64	>128	>128
**Reference**				
Racemic MQ	4	2–4	16–32	16–32
CLR	2	≤0.125	>64	0.25–0.5
RIF	>16	0.5–2	>8	>8
EMB	ND	ND	ND	ND

Abbreviation: ND, not determined.

a Clinical isolate of *M. avium* from a 76‐year‐old patient; sputum sample collected at the Amiens‐Picardie University Hospital.

b Susceptible clinical isolate of *M. xenopi* from a 60‐year‐old patient with COPD at the stage of respiratory failure and co‐infected with *Aspergillus*; sputum sample collected at the Amiens‐Picardie University Hospital.

c Clinical isolate of *M. abscessus* sp. *bolletii* (smooth morphotype), CLR‐resistant, isolated from a 7‐year‐old patient with cystic fibrosis; sputum sample collected at the Amiens‐Picardie University Hospital.

d Clinical isolate of *M. abscessus* sp. *massiliense* (smooth morphotype), macrolide‐susceptible, isolated from a 26‐year‐old patient with cystic fibrosis; sputum sample collected at the Amiens‐Picardie University Hospital.

### 2.4 Checkerboard Assays

In vitro checkerboard method is a technique used to identify synergistic, additive, or antagonistic effects resulting from the combination of two antibiotics compared with each antibiotic used alone. The observed effect is expressed as the fractional inhibitory concentration index (FICI), calculated as the sum of the ratios between the MIC of antibiotics in combination and the MIC of the antibiotic alone.

Four combinations of two antibiotics were tested against *M. avium* ATCC 700898: EMB/CLR, EMB/(*R*)‐**4c**, CLR/(*R*)‐**4c**, and RIF/(*R*)‐**4c** (Table 6). The in vitro activity against *M. avium* is conserved for (*R*)‐**4c** and for the three reference antibiotics. The FICI value calculated for the four combinations was equal to 1 ‍or 1.5, indicating an additive effect between EMB and CLR as well as between the hit (*R*)‐**4c** and each of the three antibiotics of the gold standard treatment. These results confirm the absence of antagonistic interactions between (*R*)‐**4c** and EMB, CLR, and RIF. Therefore, these molecules could be associated in tri‐therapy to limit the risk of resistance development, which is an asset for the long treatment of MAC lung infections. Moreover, (*R*)‐**4c** could be an alternative to RIF which is a potent enzymatic inducer.

**TABLE 6:** FICI value on *M. avium* ATCC700898 strain.

**Combinations,** ‍(ATB A/ATB B)	**MIC, µg/mL** **alone**	**MIC, µg/mL** **in combination** a	**FICI value** b	Mode of interaction^c^
EMB/CLR	4/0.125	2/0.125	1.5	Additive effect
EMB/(*R*)‐**4c**	4/8	2/4	1.0	
CLR**/**(*R*)‐**4c**	0.5/16	0.25/8	1.0	
RIF/(*R*)‐**4c**	0.5/16	0.5/8	1.5	

Abbreviation: ATB = antibiotic.

a MIC of two antibiotics (ATB) in combination determined by checkerboard assays; see conditions in the Experimental Section.

b Fractional inhibitory concentration index = (MIC of ATB A in combination/MIC of ATB A alone) + (MIC of ATB B in combination/MIC of ATB B alone).

c Two ATB have a synergistic effect when FICI ≤ 0.5, an additive effect when 0.5 < FICI < 2 and an antagonist effect when 2 ≥ FICI.

## 3 Conclusion

In this work, we reported an asymmetric synthesis of seventeen amino‐alcohol quinolines, designed based on the structure of MQ and our previous study, in order to identify new antimycobacterial candidates. Amino‐alcohol quinolines were synthesized in five or seven steps, with overall yields ranging from 1% to 31% and with ee ranging from 84% to 97%, except for **1** ‍and **3a**,**b**, which were obtained in partially and fully racemized forms. In parallel, in silico and experimental PC and PK properties were measured. The results showed that trifluoromethyl substituents and piperazine moiety increased the lipophilicity and aqueous solubility of amino‐alcohol quinolines, respectively. Thus, the combination of these two motifs on the quinoline core resulted in an optimal lipophilicity/hydrophilicity balance, which promotes good apparent membrane permeability and oral bioavailability. All amino‐alcohol quinolines were evaluated against *M. avium* reference strain and then against other slow‐ and rapid‐growing NTM species. Five selected *N*‐alkylpiperazine amino‐alcohol quinolines showed similar results against clinical isolates confirming the interest of our amino‐alcohol quinolines additional evaluations against other clinical strains, particularly those resistant to macrolides should be evaluated. The results showed that amino‐alcohol quinolines substituted by both two trifluoromethyl groups and piperazine moiety were the most active against NTM strains and the least cytotoxic on the HepG2 cell line, confirming the importance of these two motifs to develop new anti‐NTM candidates. In general, stereochemistry does not affect antimycobacterial activity but slightly affects cytotoxicity as the enantiomers (*R*) are less cytotoxic. The most promising compound (*R*)‐**4c** showed addictive effect in combination with CLR, EMB, and RIF against *M. avium*. These findings supported the potential of amino‐alcohol quinolines as anti‐NTM agents and emphasize the importance of pursing pharmacomodulation efforts to improve their SI.

## Funding

Elise Si Ahmed Charrier was the recipient of a grant from “Université de Picardie Jules Verne.”

## Conflicts of Interest

The authors declare no conflicts of interest.

## Supporting information

Supplementary Material

## Data Availability

The data that support the findings of this study are available in the Supplementary Information of this article.
