# Supplementary material for: Synthesis, Characterization, and In Vitro Evaluation of New Amino‐Alcohol Quinolines as Promising Antimycobacterial Agents
Source: ChemMedChem. 2026 Oct 22;21(20):e70543. doi: 10.1002/cmdc.70543 (PMC13691408; doi:10.1002/cmdc.70543)
Supplement: Supplementary file 1 [file CMDC-21-e70543-s001.pdf]

Supporting Information

Synthesis, characterization and *in vitro* evaluation of new amino-alcohol-quinolines as promising antimycobacterial agents

Elise Si Ahmed Charrier,[a] François Peltier, [a,b] Jean-François Raimbert, [a] Marie Perret, [a] Claire Andréjak,[a] Alexandra Dassonville-Klimpt,[a] and Pascal Sonnet* [a]

[a] AGents Infectieux, Résistance et chimiothérapie, UR4294, UFR de Pharmacie, Université de Picardie Jules Verne, Campus du Thil, 80025, Amiens, France.
[b] Département de Bactériologie, CHU Amiens-Picardie, 80000, Amiens, France.
[c] Service de Pneumologie, CHU Amiens-Picardie, 80000, Amiens, France.

Table of content

S1. Chemistry. 2

S2. Mosher's esters synthesis and analysis. 20

S3. ^{1}H and ^{13}C-NMR spectra of compounds **1**-**4a**-**f**. 21

S4. HRMS spectra of compounds **1**-**4a**-**f**. 30

S5. HPLC chromatograms of compounds **1**-**4a**-**f**. 39

S6. Physicochemical and pharmacokinetic properties. 47

S7. *In vitro* antimycobacterial activity. 48

S8. *In vitro* cytotoxicity. 49

S9. Checkerboard assays. 50

S1. Chemistry.

General information

Reactants and reagents. Chemical reagents and solvents were obtained from commercial suppliers and used without further purification. Anhydrous solvents were purchased in sealed containers from commercial suppliers or dried on the PureSolv® MD 5 solvent purification system purchased from Serlabo Technologies. Moisture or air sensitive reactions were performed under argon atmosphere, using dry solvents and all glassware were flushed under argon prior to use.

Microwave-assisted synthesis. Microwave assisted chemical reactions were conducted on a CEM Discover® 2.0 microwave synthesizer.

Chromatography techniques. Thin Layer Chromatographies (TLC) were performed on silica gel 60 precoated aluminium plates ALUGRAM® Xtra SIL G/UV254 (Macherey-Nagel). Visualization was achieved by fluorescence quenching under UV light at 254 nm or 365 nm, and stained by one of the following reagents: solution of $KMnO_4$ or solution of phosphomolybdic acid.

Purifications with chromatography techniques were conducted by two methods: column chromatography or Flash chromatography. Depending on the compound and the solubility, liquid or solid deposit on Celite® 545 were performed. Column chromatography purifications used silica gel Kieselgel 60 (40-63 µm, Merck) or NH_2 gel (15 µm, Interchim). Flash chromatography purifications were performed on prepacked columns (silica cartridges purchased from Reveleris, Interchim or Claricep) by a Puriflash® XS520plus apparatus from Interchim.

Gas chromatography (GC) analyses were performed on a ZB-5-MS (Phenomenex) column by a Shimadzu GC-MS QP2010S apparatus with a quadrupole electron ionization (EI) source. The gaseous vehicle was H_2, used at 4.5 mL/min. Analytical samples were performed in DCM or MeOH.

Enantiomeric excesses (ee) were determined by high performance liquid chromatography (HPLC) on a Shimadzu LC-20AD apparatus using UV detection. Depending of the compound structure, different analytical chiral columns were used: Chiralpak® IG, IF, ID, IC, IB or IA (for all, particle size: 5 µM, dimensions: 4.6 x 250 mm). A mix of heptane and isopropanol was used as mobile phase at 1 mL/min flow rate. Analytical samples were performed in the same mixture (< 5% of isopropanol) at 1 mg/mL.

Melting point. Melting points (m.p.) were estimated on a Stuart Automatic Melting Point SMP-50 apparatus.

Spectrometry techniques. NMR spectra were recorded on a Bruker Advance 400 spectrometer (400 and 101 MHz for ^1H and ^{13}C nuclei respectively). $CDCl_3$, CD_3OD or $(CD_3)_2SO$ were used as solvents. ^1H and ^{13}C chemical shift (δ) were quoted in part per million (ppm) relative to residual solvent peaks as appropriate. Complete assignment of all NMR signals was performed using a combination of COSY, HSQC, HMBC and DEPTQ experiments. NMR coupling constants (*J*) were reported in Hertz (Hz) and splitting patterns were indicated as follows: singlet (s), doublet (d), triplet (t), quartet (q), multiplet (m), or a combination thereof.

MS spectra were recorded on a Shimadzu LCMS-2020 equipped with a positive or negative quadrupole electrospray ionization (ESI) source. Analytical samples were performed in MeOH or in a mixture of H_2O and MeCN (1/1) at 1 mg/mL.

HRMS spectra were recorded on an ACQUITY UPLC HClass system (Waters-Micromass, Manchester, UK) coupled with a SYNAPT G2-Si Q-TOF hybrid quadrupole time of flight instrument (Waters-Micromass, Manchester, UK), equipped with a positive or negative quadrupole ESI source. Analytical samples were performed in MeOH or in a mixture of H_2O and MeCN (1/1 v/v) at 1 mg/mL.

IR spectra were recorded on a Jasco FT/IR4200 spectrometer equipped with an ATRGolden gate system. Absorptions are reported in wavenumbers (cm^{-1}). Only peaks of interest were reported.

Polarimetry. Optical rotations were measured by a Jasco P-2000 polarimeter with Peltier thermostated cell hold PTC-262. Specific rotations are reported in 10^{-1} deg.cm^2.g^{-1} and concentrations in g/100 mL. Analytical samples were performed in MeOH or DCM at 1 mg/mL.

General procedures

Procedure A: Bromination. The solid phosphorus oxybromide (1 or 2 eq.) was warmed up to 90 °C under argon to obtain a liquid. The corresponding 4-hydroxyquinoline (1 eq.) was added and the mixture was stirred at 150 °C. After cooling at 25 °C, cold H_2O was added. The precipitate was filtered and washed before dried under reduced pressure.

Procedure B: Suzuki-Miyaura type coupling. To a solution of corresponding 4-bromoquinoline (1 or 1.7 eq.) in a mixture THF/H_2O 9/1 were successively added potassium vinyltrifluoroborate (1 eq.), cesium carbonate (2.8 eq.) and $PdCl_2$(dppf).CH_2Cl_2 (0.03-0.1 eq.). The mixture was stirred at reflux (90 °C), under argon. The THF was evaporated, then the product was extracted with DCM. The organic layers were brought together, dried over Na_2SO_4, filtered and concentrated in vacuo. The product was purified by a chromatography on silica gel column.

Procedure C: Asymmetric dihydroxylation of Sharpless. To a solution of AD-mix α or AD-mix β and $K_2[OsO_2(OH)_4]$ (0.1 eq.) in *t*BuOH/H_2O (1/1) at 0 °C under argon, was added the corresponding 4-vinylquinoline (1 eq.). The mixture was stirred at 20 °C for 16 h. Na_2SO_3 was added at 0 °C for 30 min then, the product was extracted with EtOAc. The organic layers were brought together, dried over Na_2SO_4 and concentrated.

Procedure D: Epoxydation. Step 1: the corresponding (*S*/*R*)-1-(quinolin-4-yl)ethane-1,2-diol (1 eq.) was suspended in anhydrous DCM. PTSA.H_2O (0.05 or 1 eq.) was added then trimethylorthoacetate (3 eq.) was added dropwise. The mixture was stirred under argon for 5 h at 20-25 °C. Step 2: the resulting solution was concentrated under reduced pressure and was solubilized in anhydrous DCM under argon. TMSBr (3 eq.) was stirred at 20-25 °C for 16 h under argon. Step3: after concentration under reduced pressure, the solution was solubilized in anhydrous MeOH and K_2CO_3 (1.5 eq.) was added. The mixture was stirred at 20-25 °C for 4 h. At the solution, saturated ammonium chloride solution was added and the product was extracted with DCM. The organic layers were brought together, dried over Na_2SO_4 and concentrated. The residue was purified by a chromatography on silica gel column.

Procedure E: Alkylation of *tert*-butyl piperazine-1-carboxylate. To a solution of *tert*-butyl piperazine-1-carboxylate (1 eq.) in anhydrous CH_3CN (0.1 M) under argon, was successively added halogenoalkane (2 eq.) and diisopropylethylamine (DIPEA) (1.5 eq.). The mixture was stirred at 20-25 °C until for the completion of the reaction. The solution was diluted in H_2O, acidified and washed with EtOAc and brine. The organic layers were brought together, dried over Na_2SO_4 and concentrated *in vacuo.* The product was purified by a chromatography on silica gel column.

Procedure F: Deprotection of *tert*-butyl 4-alkylpiperazine-1-carboxylate. To a solution of *tert*-butyl 4-alkylpiperazine-1-carboxylate **35**-**39** (1 eq.) in anhydrous DCM (0.1 M) was added TFA. The mixture was stirred, under argon, at 16-18 °C for the completion of the reaction. The TFA was removed under reduced pressure.

Procedure G: Basic treatment of 4-alkylpiperazin-1-ium 2,2,2-trifluoroacetate salt. To a solution of 4-alkylpiperazin-1-ium 2,2,2-trifluoroacetate salt (136 mg, 0.34 mmol, 1 eq.) in mixture of DCM/MeOH 3/1 was added NaOH 1N until a pH of 12-14. The product was extracted with DCM. The organic layers were brought together, dried over Na_2SO_4 and concentrated *in vacuo*.

Procedure H: Coupling between epoxides (*S*/*R*)-10-13 and monoalkylated piperazines 5-9. To a solution of (*S*,*R*)-epoxides (1 eq.) in EtOH was added the corresponding piperazine (3 eq.). The resulting solution was stirred at 130 °C for 30 min or 1 h under microwaves (150 W). The product was concentrated *in vacuo* and purified by a chromatography on silica gel column.

Synthesis of compounds 1-4a-f.

Synthesis of 4-hydroxy-8-(trifluoromethyl)quinoline 20.

Diethyl 2-(((2-(trifluoromethyl)phenyl)amino)methylene)malonate 24. A solution of 2-(trifluoromethyl)aniline **22** (1.56 mL, 12.4 mmol, 1 eq.), diethyl ethoxymethylene malonate **23** (3.0 mL, 14.8 mmol, 1.2 eq.) and NaOH (657 mg, 16.4 mmol, 1.3 eq.) was heated to 110 °C for 24 h. After cooling at 25 °C, the product was washed with H_2O (100 mL) and it was extracted with EtOAc (3x25 mL). The organic layers were brought together, dried over Na_2SO_4, filtered and concentrated *in vacuo*. The product was purified by a chromatography on silica gel column with a gradient cyclohexane/EtOAc 100/0 to 50/50. The product **24** was afforded as a yellow solid. Yield: 41%. R_f: 0.55 (cyclohexane/EtOAc 7/3). m.p. 90-92 °C. ^{1}H NMR (400 MHz, $CDCl_3$) δ 11.36-11.33 (m, 1H), 8.45 (d, ^3J = 13.1 Hz, 1H), 7.65 (d, ^3J = 7.8 Hz, 1H), 7.62-7.57 (m, 1H), 7.37 (d, ^3J = 8.2 Hz, 1H), 7.27-7.23 (m, 1H), 4.34 (q, ^3J = 7.1 Hz, 2H), 4.26 (q, ^3J = 7.1 Hz, 2H), 1.37 (t, ^3J = 7.1 Hz, 3H), 1.33 (t, ^3J = 7.1 Hz, 3H) ppm. ^{13}C NMR (101 MHz, $CDCl_3$) δ 168.3, 165.7, 151.9, 137.9, 133.6, 127.1 (q, ^3J = 5.3 Hz), 125.2, 124.5, 123.8 (q, ^1J = 272.8 Hz), 122.5, 120.3 (q, ^2J = 60.5 Hz), 118.9, 96.5, 60.8, 60.5, 14.5, 14.4 ppm. IR ν_{max}: 2989, 1708, 1654, 1161, 752 cm^{-1}. MS (ESI) *m/z* 395 $[M+Na+CH_3CN]^+$.

Ethyl 4-hydroxy-8-(trifluoromethyl)quinoline-3-carboxylate 25. Diethyl 2-(((2-(trifluoromethyl)phenyl)amino)methylene)malonate **24** (1.67 g, 5.0 mmol) was solubilized in diphenylether (16 mL), then a mixture was heated at 250 °C for 6 h. After cooling at 25 °C, cyclohexane (24 mL) was added and stirred for 10 minutes. The solid precipitated was filtered and dried. The product **25** was afforded as a beige solid. Yield: 70%. R_f: 0.40 (cyclohexane/EtOAc 7/3). m.p. 286-289 °C. ^{1}H NMR (400 MHz, CD_3OD) δ 8.67-8.62 (m, 2H), 8.12 (d, ^3J = 7.7 Hz, 1H), 7.60 (t, ^3J = 7.1 Hz, 1H), 4.36 (q, ^3J = 7.1 Hz, 2H), 1.39 (t, ^3J = 7.1 Hz, 3H) ppm. ^{13}C NMR (101 MHz, CD_3OD) δ 169.8, 166.3, 150.9, 147.3, 130.4 (q, ^3J = 6.2 Hz), 127.6, 125.0, 124.8, 111.2, 104.2, 62.1, 13.9 ppm. IR ν_{max}: 3239, 3193, 1708, 1653, 1164, 1090, 753 cm^{-1}. MS (ESI) *m/z* 349 $[M+Na+CH_3CN]^+$.

4-hydroxy-8-(trifluoromethyl)quinoline-3-carboxylic acid 26. To a solution of sodium hydroxide (372 mg, 0.01 mol, 2.9 eq) in water (10 mL), ethyl 4-hydroxy-8-(trifluoromethyl)quinoline-3-carboxylate **25** (974 mg, 3.4 mmol, 1 eq.) was stirred and heated at 100 °C for 19 h. After cooling at 25 °C, the mixture was acidified with hydrochloric acid (15 mL, 1M) to pH 2-3. The product was filtered and dried. The product **26** was obtained as a white solid. Yield: 91%. R_f: 0.79 (DCM/MeOH 9/1). m.p. 253-256 °C. ^{1}H NMR (400 MHz, $(CD_3)_2SO$) δ 12.16 (s, 1H), 8.21 (s, 1H), 8.16 (d, ^3J = 8.2 Hz, 1H), 7.87 (d, ^3J = 7.6 Hz, 1H), 7.30 (t, ^3J = 7.9 Hz, 1H) ppm. ^{13}C NMR (101 MHz, $(CD_3)_2SO$) δ 177.7, 165.5, 146.4, 135.4, 132.1 (q, ^3J = 5.3 Hz), 130.6, 125.9, 125.5, 123.2 (q, ^1J = 273.0 Hz), 118.1 (q, ^3J = 32.1 Hz) ppm. IR ν_{max}: 3182, 2342, 1718, 1619, 1169, 1129, 1105, 759 cm^{-1}. MS (ESI) *m/z* 258 $[M+H]^+$.

4-hydroxy-8-(trifluoromethyl)quinoline 20. 4-hydroxy-8-(trifluoromethyl)quinoline-3-carboxylic acid **26** (759 mg, 2.9 mmol) was solubilized in diphenylether (6 mL). Then, the reaction mixture was stirred and heated at 250 °C for 2 h. After cooling at 25 °C, cyclohexane (32 mL) was added. The resulting solution was stirred at room temperature for 1 h. The precipitated was filtered and washed with a mixture of cyclohexane/EtOAc 1/2 (90 mL). The product **20** was obtained as a white solid. Yield: 65%. R_f: 0.62 (DCM/MeOH 9.9/0.1). m.p. 216-220 °C. ^{1}H NMR (400 MHz, CD_3OD) δ 8.54 (d, ^3J = 8.2 Hz, 1H),

8.08 (d, ^3J = 8.3 Hz, 1H), 7.96 (d, ^3J = 7.5 Hz, 1H), 7.54-7.50 (m, 1H), 6.40 (d, ^3J = 7.4 Hz, 1H) ppm. ^{13}C NMR (101 MHz, CD_3OD) δ 179.9, 142.2, 137.5, 131.6, 130.8, 128.0, 124.3 (q, ^1J = 272.0 Hz), 119.8, 110.9 ppm. IR $ν_{max}$: 2362, 1636, 1160, 1119, 994, 751 cm^{-1}. MS (ESI) *m/z* 214 $[M+H]^+$.

Bromination 27-30.

4-bromoquinoline 27. The solid phosphorus oxybromide (4.86 g, 17 mmol, 1 eq.) was reacted with the 4-hydroxyquinoline **18** (2.47 g, 17 mmol, 1 eq.) according to the procedure A. The precipitate was filtered and washed before dried under reduced pressure to afford **27** as a brown oil. Yield: 96%. R_f: 0.4 (cyclohexane/EtOAc 4/1). ^{1}H NMR (400 MHz, CD_3OD) δ 9.09 (d, ^3J = 5.9 Hz, 1H), 8.59 (dt, ^3J = 8.4 Hz, ^4J = 0.9 Hz, 1H), 8.53 (d, ^3J = 5.9 Hz, 1H), 8.31-8.26 (m, 2H), 8.15-8.11 (m, 1H) ppm. ^{13}C NMR (101 MHz, CD_3OD) δ 146.7, 142.9, 136.9, 132.5, 129.4, 127.6, 123.0, 121.4 ppm. IR $ν_{max}$: 3063, 2470, 1577, 1375, 1195, 1055, 753, 650 cm^{-1}. GC-MS *m/z* 207.

4-bromo-2-(trifluoromethyl)quinoline 28. The solid phosphorus oxybromide (2.85 g, 9.9 mmol, 1 eq.) was reacted with the 4-hydroxy-2-(trifluoromethyl)quinoline **19** (2.12 g, 9.9 mmol, 1 eq.) according to the procedure A. The precipitate was filtered and washed before dried under reduced pressure to afford **28** as a yellow oil. Yield: 56%. R_f: 0.71 (cyclohexane/EtOAc 9.5/0.5). ^{1}H NMR (400 MHz, CD_3OD) δ 8.32-8.22 (m, 2H), 8.03 (s, 1H), 7.91-7.86 (m, 1H), 7.81-7.76 (m, 1H) ppm. ^{13}C NMR (101 MHz, CD_3OD) δ 147.9, 144.8, 136.0, 131.8, 130.8, 128.7, 127.0, 121.1 (q, ^3J = 2.4 Hz), 121.0 (q, ^1J = 275.9 Hz) ppm. IR $ν_{max}$: 3069, 1557, 1378, 1179, 1090, 760 cm^{-1}. GC-MS *m/z* 276.

4-bromo-8-(trifluoromethyl)quinoline 29. The solid phosphorus oxybromide (1.16 g, 4.0 mmol, 2 eq.) was reacted with the 4-hydroxy-8-(trifluoromethyl)quinoline **20** (432 mg, 2.0 mmol, 1 eq.) according to the procedure A. The precipitate was filtered and washed before dried under reduced pressure to afford **29** as a brown solid. Yield: 96%. R_f: 0.7 (cyclohexane/EtOAc 9.5/0.5). m.p. 136-138 °C. ^{1}H NMR (400 MHz, CD_3OD) δ 8.81 (d, ^3J = 4.8 Hz, 1H), 8.56 (d, ^3J = 8.6 Hz, 1H), 8.27 (d, ^3J = 7.3 Hz, 1H), 8.06 (d, ^3J = 4.8 Hz, 1H), 7.86 (t, ^3J = 7.9 Hz, 1H) ppm. ^{13}C NMR (101 MHz, CD_3OD) δ 151.5, 145.1, 136.9, 132.9, 130.9 (q, ^3J = 5.6 Hz), 129.8, 129.2, 128.4, 127.8, 125.1 (q, ^1J = 272.4 Hz) ppm. IR $ν_{max}$: 1588, 1312, 1126, 1082, 994, 972 cm^{-1}. GC-MS *m/z* 276.

4-bromo-2,8-*bis*(trifluoromethyl)quinoline 30. The solid phosphorus oxybromide (1.46 g, 5.0 mmol, 1 eq.) was reacted with the 2,8-*bis*(trifluoromethyl)-4-hydroxyquinoline **21** (1.50 g, 5.0 mmol, 1 eq.) according to the procedure A. The precipitate was filtered and washed before dried under reduced pressure to afford **30** as a white solid. Yield: 95%. R_f: 0.8 (cyclohexane/EtOAc 4/1). m.p. 62-65 °C. ^{1}H NMR (400 MHz, $CDCl_3$) δ 8.49 (d, ^3J = 7.3 Hz, 1H), 8.24 (d, ^3J = 7.4 Hz, 1H), 8.13 (s, 1H), 7.84 (t, ^3J = 8.0 Hz, 1H) ppm. ^{13}C NMR (101 MHz, $CDCl_3$) δ 148.5 (q, ^2J = 36.3 Hz), 144.2, 136.2, 131.3, 130.2 (q, ^3J = 5.4 Hz), 129.7 (q, ^2J = 30.7 Hz), 129.1, 128.7, 123.3 (q, ^1J = 274.8 Hz), 122.2 (q, ^3J = 2.3 Hz), 120.6 (q, ^1J = 275.6 Hz) ppm. IR $ν_{max}$: 1575, 1422, 1301, 1137, 1099, 876, 766, 691 cm^{-1}. GC-MS *m/z* 345.

Suzuki-Miyaura type coupling 31-34.

4-vinylquinoline 31. To a solution of 4-bromoquinoline **27** (4.61 g, 22 mmol, 1.7 eq.) in a mixture THF/H_2O 9/1 (49 mL) were successively added potassium vinyltrifluoroborate (1.74 g, 13 mmol, 1 eq.), cesium carbonate (12.0 g, 37 mmol, 2.8 eq.) and $PdCl_2$(dppf).CH_2Cl_2 (53 mg, 0.7 mmol, 0.05 eq.) according to the procedure B. The product was purified by a chromatography on silica gel column with

a gradient cyclohexane/EtOAc 100/0 to 80/20. The product **31** was afforded as a yellow liquid. Yield: 67%. R_f: 0.2 (cyclohexane/EtOAc 4/1). ^{1}H NMR (400 MHz, $CDCl_3$) δ 8.75 (d, ^3J = 4.6 Hz, 1H), 8.04 (d, ^3J = 8.4 Hz, 1H), 7.91 (d,^3J = 7.6 Hz, 1H), 7.57 (ddd, ^3J = 8.4 Hz, ^3J = 6.8 Hz, ^4J = 1.4 Hz, 1H), 7.39 (ddd, ^3J = 8.3 Hz, ^3J = 6.8 Hz, ^4J = 1.3 Hz, 1H), 7.28-7.26 (m, 1H), 7.24-7.20 (m, 1H), 5.80 (dd, ^3J = 17.3 Hz, ^4J = 1.2 Hz, 1H), 5.49 (dd, ^3J = 11.0 Hz, ^4J = 1.2 Hz, 1H) ppm. ^{13}C NMR (101 MHz, $CDCl_3$) δ 150.0, 148.3, 143.1, 131.7, 129.8, 129.0, 126.3, 125.9, 123.3, 120.4, 117.1 ppm. IR ν_{max}: 3064, 2922, 1572, 1499, 1309, 1143, 986, 913, 761 cm^{-1}. GC-MS *m/z* 154-157.

2-(trifluoromethyl)-4-vinylquinoline 32. To a solution of 4-bromo-2-(trifluoromethyl)quinoline **28** (1.26 g, 4.6 mmol, 1 eq.) in a mixture THF/H_2O 9/1 (40 mL) were successively added potassium vinyltrifluoroborate (1.23 g, 9.2 mmol, 2 eq.), cesium carbonate (3.73 g, 11.5 mmol, 2.8 eq.) and $PdCl_2$(dppf).CH_2Cl_2 (34 mg, 0.04 mmol, 0.01 eq.) according to the procedure B. The residue was first purified by a chromatography on silica gel column (cyclohexane/DCM, 100/0 to 70/30), then by a chromatography on silica gel column (cyclohexane/EtOAc, 100/0 to 95/05) to afford the product **32** as a colorless liquid. Yield: 62%. R_f: 0.15 (cyclohexane/DCM 9/1). ^{1}H NMR (400 MHz, $CDCl_3$) δ 8.20 (d, ^3J = 8.5 Hz, 1H), 8.12-8.09 (m, 1H), 7.80-7.76 (m, 2H), 7.67-7.62 (m, 1H), 7.45-7.37 (m, 1H), 6.10 (dd, ^3J = 17.4 Hz, ^4J = 1.9 Hz, 1H), 5.75 (dd, ^3J = 11.1 Hz, ^4J = 1.9 Hz, 1H) ppm. ^{13}C NMR (101 MHz, $CDCl_3$) δ 147.7, 145.9, 131.4, 130.7, 130.6, 128.6, 126.8, 123.6, 122.4, 121.8 (q, ^4J = 274.5 Hz), 113.2 ppm. IR ν_{max}: 1587, 1426, 1345, 1129, 1095, 982, 899, 761 cm^{-1}. HRMS (ESI) *m/z* calculated for $C_{12}H_8F_3N$ $[M+H]^+$ 224.0687, found 224.0685.

8-(trifluoromethyl)-4-vinylquinoline 33. To a solution of 4-bromo-8-(trifluoromethyl)quinoline **29** (1.93 g, 7.0 mmol, 1 eq.) in a mixture THF/H_2O 9/1 (60 mL) were successively added potassium vinyltrifluoroborate (1.88 g, 14.0 mmol, 2 eq.), cesium carbonate (5.71 g, 17.5 mmol, 2.5 eq.) and $PdCl_2$(dppf).CH_2Cl_2 (52 mg, 0.06 mmol, 0.01 eq.) according to the procedure B. The residue was first purified by a chromatography on silica gel column (cyclohexane/EtOAc, 100/0 to 95/05) to afford the product **33** as a white solid. Yield: 57%. R_f: 0.36 (cyclohexane/DCM 9/1). m.p. 76-78 °C. ^{1}H NMR (400 MHz, $CDCl_3$) δ 8.99 (d, ^3J = 4.5 Hz, 1H), 8.25 (d, ^3J = 7.9 Hz, 1H), 8.04 (d, ^3J = 7.2 Hz, 1H), 7.56 (t, ^3J = 7.9 Hz, 1H), 7.51 (d, ^3J = 4.5 Hz, 1H), 7.36 (dd, ^3J = 17.3 Hz, ^3J = 11.0 Hz, 1H), 5.96 (dd, ^3J = 17.3 Hz, ^4J = 1.0 Hz, 1H), 5.68 (dd, ^3J = 11.0 Hz, ^4J = 1.0 Hz, 1H) ppm. ^{13}C NMR (101 MHz, $CDCl_3$) δ 151.1, 145.0, 143.9, 131.7, 130.6, 128.1, 128.1, 127.9 (q, ^3J = 5.7 Hz), 126.7, 127.3 (q, ^4J = 273.3 Hz), 125.1, 121.8, 118.4 ppm. IR ν_{max}: 1591, 1427, 1309, 1124, 1102, 1071, 986, 758 cm^{-1}. GC-MS *m/z* 224.

2,8-*bis*(trifluoromethyl)-4-vinylquinoline 34. To a solution of 4-bromo-2,8-*bis*(trifluoromethyl)quinoline **30** (4.27 g, 12.4 mmol, 1 eq.) in a mixture THF/H_2O 9/1 (130 mL) were successively added potassium vinyltrifluoroborate (1.74 g, 13.0 mmol, 1 eq.), cesium carbonate (12.4 g, 38.2 mmol, 3 eq.) and $PdCl_2$(dppf).CH_2Cl_2 (30 mg, 0.04 mmol, 0.003 eq.) according to the procedure B. The residue was purified by a chromatography on silica gel column (cyclohexane/DCM, 100/0 to 90/10) to afford the product **34** as a white solid. Yield: 77%. R_f: 0.27 (cyclohexane/DCM 9/1). m.p. 79-81 °C. ^{1}H NMR (400 MHz, $CDCl_3$) δ 8.36 (d, ^3J = 7.3 Hz, 1H), 8.18 (d, ^3J = 7.3 Hz, 1H), 7.88 (s, 1H), 7.74 (t, ^3J = 7.9 Hz, 1H), 7.45 (dd, ^3J = 17.3 Hz, ^3J = 11.0 Hz, 1H), 6.10 (dd, ^3J = 17.3 Hz, ^4J = 0.7 Hz, 1H), 5.84 (dd, ^3J = 11.1 Hz, ^4J = 0.7 Hz, 1H) ppm. ^{13}C NMR (101 MHz, $CDCl_3$) δ 148.4 (q, ^2J = 35.9 Hz), 146.2, 144.0, 131.0, 129.4, 129.0 (q, ^3J = 5.7 Hz), 127.9, 124.9, 123.5 (q, ^1J = 234.4 Hz), 123.2, 121.3 (q, ^1J = 234.4 Hz), 114.1 (d,^4J = 2.2 Hz) ppm. IR ν_{max}: 1590, 1426, 1303, 1137, 1109, 982, 903, 767 cm^{-1}. GC-MS *m/z* 291.

Asymmetric dihydroxylation of Sharpless (*S*/*R*)-14-17.

(*S*)-1-(quinolin-4-yl)ethane-1,2-diol 14. To the solution of AD-mix α (2.53 g) and $K_2[OsO_2(OH)_4]$ (126 mg, 0.3 mmol, 0.1 eq.) in *t*BuOH/H_2O (1/1) (50 mL) at 0 °C under argon was added 4-vinylquinoline **31** (522 mg, 3.4 mmol, 1 eq.) according to the procedure C. The product (*S*)-**14** was obtained as a yellow oil. Yield: 94%. R_f: 0.29 (DCM/MeOH 9.9/0.1). ^{1}H NMR (400 MHz, CD_3OD) δ 8.81 (d, ^3J = 4.6 Hz, 1H), 8.18 (d, ^3J = 8.0 Hz, 1H), 8.03 (d, ^3J = 8.5 Hz, 1H), 7.72 (d, ^3J = 4.5 Hz, 1H), 7.70-7.67 (m, 1H), 7.57 (m, 1H), 5.55 (dd, ^3J = 6.7 Hz, ^3J = 3.8 Hz, 1H), 3.89 (dd, ^2J = 11.6 Hz, ^3J = 3.7 Hz, 1H), 3.71 (dd, ^2J = 11.6 Hz, ^3J = 7.0 Hz, 1H) ppm. ^{13}C NMR (101 MHz, CD_3OD) δ 150.9, 150.6, 148.6, 130.7, 129.9, 128.0, 127.4, 124.6, 119.9, 71.8, 67.9 ppm. IR ν_{max}: 3270, 2457, 1591, 1362, 1102, 756 cm^{-1}. MS (ESI) m/z 190 $[M+H]^+$. Chiral HPLC 94% ee, Chiralpak IG column, heptane/isopropanol 90/10, flow 1.0 mL/min, tr(*S*) = 31.0 min, tr(*R*) = 43.4 min. $[\alpha]_D^{21}$ = +79 (c = 0.1, MeOH).

(*R*)-1-(quinolin-4-yl)ethane-1,2-diol 14. To a solution of AD-mix β (2.531 g) and $K_2[OsO_2(OH)_4]$ (126 mg, 0.3 mmol, 0.1 eq.) in *t*BuOH/H_2O (1/1) (50 mL) at 0 °C under argon, was added 4-vinylquinoline **31** (525 mg, 3.4 mmol, 1 eq.) according to the procedure C. The product (*R*)-**14** was obtained as a yellow oil. Yield: 95%. MS (ESI) *m/z* 190 $[M+H]^+$. Chiral HPLC 95% ee, Chiralpak IG column, heptane/isopropanol 90/10, flow 1.0 mL/min, tr(*S*) = 31.7 min, tr(*R*) = 41.9 min. $[\alpha]_D^{21}$ = -70 (c = 0.1, MeOH). R_f, NMR (^{1}H and ^{13}C) and IR were identical to the enantiomer (*S*)-**14**.

(*S*)-1-(2-(trifluoromethyl)quinolin-4-yl)ethane-1,2-diol 15. To a solution of AD-mix α (1.430 g) and $K_2[OsO_2(OH)_4]$ (49 mg, 0.1 mmol, 0.1 eq.) in *t*BuOH/H_2O (1/1) (35 mL) at 0 °C under argon, was added 2-(trifluoromethyl)-4-vinylquinoline **32** (294 mg, 1.3 mmol, 1 eq.) according to the procedure C. The product (*R*)-**15** was afforded as a white solid. Yield: 83%. R_f: 0.70 (DCM/MeOH 9.5/0.5). m.p. 265-267 °C. ^{1}H NMR (400 MHz, CD_3OD) δ 8.28 (d, ^3J = 8.1 Hz, 1H), 8.21 (d, ^3J = 8.6 Hz, 1H), 8.06 (s, 1H), 7.91-7.85 (m, 1H), 7.80-7.76 (m, 1H), 7.57 (m, 1H), 5.62-5.60 (m, 1H), 3.90 (dd, ^2J = 11.6 Hz, ^3J = 3.8 Hz, 1H), 3.73 (dd, ^2J = 11.6 Hz, ^3J = 6.5 Hz, 1H) ppm. ^{13}C NMR (101 MHz, CD_3OD) δ 153.3, 148.7 (q, ^2J = 34.2 Hz), 148.4, 131.8, 131.3, 129.9, 128.0, 124.7, 121.8, 115.3 (q, ^3J = 2.4 Hz), 71.7, 67.9 ppm. IR ν_{max}: 3265, 1600, 1363, 1130, 760 cm^{-1}. HRMS (ESI) *m/z* calculated for $C_{12}H_{10}F_3NO_2$ $[M+H]^+$ 258.0742, found 258.0742. Chiral HPLC 93% ee, Chiralpak IG column, heptane/isopropanol 95/05, flow 1.0 mL/min, tr(*S*) = 16.7 min, tr(*R*) = 29.1 min. $[\alpha]_D^{21}$ = +20 (c = 0.05, MeOH).

(*R*)-1-(2-(trifluoromethyl)quinolin-4-yl)ethane-1,2-diol 15. To a solution of AD-mix β (1.430 g) and $K_2[OsO_2(OH)_4]$ (49 mg, 0.1 mmol, 0.1 eq.) in *t*BuOH/H_2O (1/1) (35 mL) at 0 °C under argon, was added 2-(trifluoromethyl)-4-vinylquinoline **32** (294 mg, 1.3 mmol, 1 eq.) according to the procedure C. The product (*R*)-**15** was obtained as a white solid. Yield: 94%. HRMS (ESI) *m/z* calculated for $C_{12}H_{10}F_3NO_2$ $[M+H]^+$ 258.0742, found 258.0734. Chiral HPLC 93% ee, Chiralpak IG column, heptane/isopropanol 95/05, flow 1.0 mL/min, tr(*S*) = 16.7 min, tr(*R*) = 27.5 min. $[\alpha]_D^{21}$ = -26 (c = 0.05, MeOH). R_f, m.p., NMR (^{1}H and ^{13}C) and IR were identical to the enantiomer (*S*)-**15**.

(*S*)-1-(8-(trifluoromethyl)quinolin-4-yl)ethane-1,2-diol 16. To a solution of AD-mix α (1.945 g) and $K_2[OsO_2(OH)_4]$ (66 mg, 0.2 mmol, 0.1 eq.) in *t*BuOH/H_2O (1/1) (48 mL) at 0 °C under argon, was added 8-(trifluoromethyl)-4-vinylquinoline **33** (400 mg, 1.8 mmol, 1 eq.) according to the procedure C. The product (*R*)-**16** was obtained as a white solid. Yield: 99%. R_f: 0.67 (DCM/MeOH 9.5/0.5). m.p. 229-231 °C. ^{1}H NMR (400 MHz, CD_3OD) δ 8.97 (d, ^3J = 4.5 Hz, 1H), 8.49 (d, ^3J = 8.2 Hz, 1H), 8.14 (d, ^3J = 7.2 Hz, 1H), 7.81 (d, ^3J = 4.5 Hz, 1H), 7.72 (t, ^3J = 7.9 Hz, 1H), 5.54 (dd, ^3J = 6.7 Hz, ^3J = 4.1 Hz, 1H), 3.86 (dd, ^2J = 11.5 Hz, ^3J = 4.1 Hz, 1H), 3.74 (dd, ^2J = 11.6 Hz, ^3J = 6.7 Hz, 1H) ppm. ^{13}C NMR (101 MHz, CD_3OD) δ 152.0,

150.4 145.5, 129.6, 129.1, 128.9 (q, ^3J = 5.0 Hz), 128.1, 126.1 (q, ^1J = 272.7 Hz), 126.4, 124.3, 120.8, 71.8, 68.0 ppm. IR √$_{max}$: 3309, 1600, 1344, 1119, 1087, 759 cm^{-1}. HRMS (ESI) *m/z* calculated for $C_{12}H_{10}F_3NO_2$ $[M+H]^+$ 258.0742, found 258.0743. Chiral HPLC 91% ee, Chiralpak IB column, heptane/isopropanol 90/10, flow 1.0 mL/min, tr(*R*) = 13.0 min, tr(*S*) = 17.6 min. $[\alpha]_D^{21}$ = +58 (c = 0.1, MeOH).

(*R*)-1-(8-(trifluoromethyl)quinolin-4-yl)ethane-1,2-diol 16. To a solution of AD-mix β (734 mg) and $K_2[OsO_2(OH)_4]$ (25 mg, 0.07 mmol, 0.1 eq.) in *t*BuOH/H_2O (1/1) (18 mL) at 0 °C under argon, was added 8-(trifluoromethyl)-4-vinylquinoline **33** (151 mg, 0.7 mmol, 1 eq.) according to the procedure C. The product (*R*)-**16** was obtained as a white solid. Yield: 99%. HRMS (ESI) *m/z* calculated for $C_{12}H_{10}F_3NO_2$ $[M+H]^+$ 258.0742, found 258.0737. Chiral HPLC 96% ee, Chiralpak IB column, heptane/isopropanol 90/10, flow 1.0 mL/min, tr(*R*) = 13.0 min, tr(*S*) = 17.9 min. $[\alpha]_D^{21}$ = -50 (c = 0.1, MeOH). R_f, m.p., NMR (^{1}H and ^{13}C) and IR were identical to the enantiomer (*S*)-**16**.

(*S*)-1-(2,8-*bis*(trifluoromethyl)quinolin-4-yl)ethane-1,2-diol 17. To the solution of AD-mix α (2.01 g) and $K_2[OsO_2(OH)_4]$ (52 mg, 0.14 mmol, 0.1 eq.) in *t*BuOH/H_2O (1/1) (50 mL) at 0 °C under argon was added 2,8-*bis*(trifluoromethyl)-4-vinylquinoline **34** (414 mg, 1.42 mmol, 1 eq.) according to the procedure C. The product (*S*)-**17** was obtained as a white solid. Yield: 99%. *R*$_f$: 0.23 (DCM/EtOAc 3/2). m.p. 144-145 °C. ^{1}H NMR (400 MHz, CD_3OD) δ 8.56 (d, ^3J = 8.6 Hz, 1H), 8.25 (d, ^3J = 7.3 Hz, 1H), 8.15 (s, 1H), 7.85 (t, ^3J = 8.0 Hz, 1H), 5.62 (dd, ^3J = 6.1 Hz, ^3J = 4.3 Hz, 1H), 3.88 (dd, ^2J = 11.6 Hz, ^3J = 4.3 Hz, 1H), 3.77 (dd, ^2J = 11.6 Hz, ^3J = 6.2 Hz, 1H) ppm. ^{13}C NMR (101 MHz, CD_3OD) δ 153.7, 149.2 (q, ^2J = 34.9 Hz), 144.8, 130.2 (q, ^2J = 30.4 Hz), 130.1 (q, ^3J = 5.6 Hz), 129.5, 128.5, 128.4, 125.1 (q, ^1J = 272.1 Hz), 122.9 (q, ^1J = 274.8 Hz), 116.3 (q, ^3J = 2.3 Hz), 71.6, 67.8 ppm. IR √$_{max}$: 2921, 2851, 1305, 1129, 1103, 774 cm^{-1}. MS (ESI) *m/z* 390 $[M+Na+CH_3CN]^+$. Chiral HPLC 91% ee, Chiralpak IG column, heptane/isopropanol 95/05, flow 1.0 mL/min, tr(*R*) = 8.9 min, tr(*S*) = 13.2 min. $[\alpha]_D^{20}$ = +31 (c = 0.1, MeOH).

(*R*)-1-(2,8-*bis*(trifluoromethyl)quinolin-4-yl)ethane-1,2-diol 17. To the solution of AD-mix β (2.01 g) and $K_2[OsO_2(OH)_4]$ (52 mg, 0.14 mmol, 0.1 eq.) in *t*BuOH/H_2O (1/1) (50 mL) at 0 °C under argon was added 2,8-*bis*(trifluoromethyl)-4-vinylquinoline **34** (414 mg, 1.42 mmol, 1 eq.) according to the procedure C. The product (*R*)-**17** was obtained as a white solid. Yield: 95%. MS (ESI) *m/z* 390 $[M+Na+CH_3CN]^+$. Chiral HPLC 91% ee, Chiralpak IG column, heptane/isopropanol 95/05, flow 1.0 mL/min, tr(*R*) = 8.8 min, tr(*S*) = 13.4 min. $[\alpha]_D^{20}$ = -37 (c = 0.1, MeOH). R_f, m.p., NMR (^{1}H and ^{13}C) and IR were identical to the enantiomer (*S*)-**17**.

Epoxydation (*S*/*R*)-10-13.

(*S*)-4-(oxiran-2-yl)quinoline 10. (*S*)-1-(quinolin-4-yl)ethane-1,2-diol **14** (273 mg, 1.4 mmol, 1 eq.) was suspended in anhydrous DCM (12 mL). PTSA.H_2O (14 mg, 0.05 mmol, 1 eq.) was added then trimethylorthoacetate (534 µL, 3.9 mmol, 2.8 eq.) was added dropwise according to the procedure D (step 1). TMSBr (534 µL, 3.9 mmol, 2.8 eq.) was added according to the procedure D (step 2). After concentration under reduced pressure, the solution was solubilized in anhydrous MeOH (12 mL) and K_2CO_3 (302 mg, 2.2 mmol, 1.6 eq.) was added according to the procedure D (step 3). The residue was purified by a chromatography on silica gel column (DCM/MeOH, 100/0 to 90/10) to afford the product (*S*)-**10** as a yellow oil. Yield: 55%. *R*$_f$: 0.4.(cyclohexane/EtOAc 3/2). ^{1}H NMR (400 MHz, $CDCl_3$) δ 8.90 (d, ^3J = 4.45 Hz, 1H), 8.16 (m, 1H), 8.11 (m, 1H), 7.78-7.73 (m, 1H), 7.64-7.59 (m, 1H), 7.37 (d, ^3J = 4.4 Hz,

1H), 4.51-4.49 (m, 1H), 3.35 (dd, ^2J = 5.8 Hz, ^3J = 4.2 Hz, 1H), 2.78 (dd, ^2J = 5.8 Hz, ^3J = 2.6 Hz, 1H) ppm. ^{13}C NMR (101 MHz, $CDCl_3$) δ 150.4, 147.7, 143.5, 130.2, 129.5, 126.9, 126.4, 122.6, 116.3, 50.6, 49.3 ppm. IR $ν_{max}$: 3262, 2596, 1737, 1598, 1509, 1379, 1221, 1115, 761 cm^{-1}. MS (ESI) *m/z* 172 $[M+H]^+$. Chiral HPLC: 50% ee, Chiralpak IG column, heptane/isopropanol 90/10, flow 1.0 mL/min, tr(*R*) = 16.02 min, tr(*S*) = 18.59 min. $[\alpha]_D^{20}$: +18 (c = 0.1, DCM).

(*R*)-4-(oxiran-2-yl)quinoline 10. (*R*)-1-(quinolin-4-yl)ethane-1,2-diol **14** (349 mg, 1.8 mmol, 1 eq.) was suspended in anhydrous DCM (16 mL). PTSA.H_2O (18 mg, 0.1 mmol, 0.05 eq.) was added then trimethylorthoacetate (681 µL, 5.0 mmol, 3 eq.) was added dropwise according to the procedure D (step 1). TMSBr (659 µL, 5.0 mmol, 3 eq.) was added according to the procedure D (step 2). After concentration under reduced pressure, the solution was solubilized in anhydrous MeOH (15 mL) and K_2CO_3 (373 mg, 2.7 mmol, 1.5 eq.) was added according to the procedure D (step 3). The residue was purified by a chromatography on silica gel column (cyclohexane/EtOAc, 100/0 to 60/40) to afford the product (*S*)-**10** as a yellow oil. Yield: 27%. MS (ESI) *m/z* 172 $[M+H]^+$. Chiral HPLC: 55% ee, Chiralpak IG column, heptane/isopropanol 90/10, flow 1.0 mL/min, tr(*R*) = 15.97 min, tr(*S*) = 18.58 min. $[\alpha]_D^{20}$: -23 (c = 0.1, DCM). R_f, NMR (^{1}H and ^{13}C) and IR were identical to the enantiomer (*S*)-**10**.

(*S*)-4-(oxiran-2-yl)-2-(trifluoromethyl)quinoline 11. (*S*)-1-(2-(trifluoromethyl)quinolin-4-yl)ethane-1,2-diol **15** (157 mg, 0.6 mmol, 1 eq.) was suspended in anhydrous DCM (16 mL). PTSA.H_2O (7 mg, 0.03 mmol, 0.05 eq.) was added then trimethylorthoacetate (234 µL, 1.8 mmol, 3 eq.) was added dropwise according to the procedure D (step 1). TMSBr (280 µL, 1.8 mmol, 3 eq.) was added according to the procedure D (step 2). After concentration under reduced pressure, the solution was solubilized in anhydrous MeOH (16 mL) and K_2CO_3 (135 mg, 1.0 mmol, 1.6 eq.) was added according to the procedure D (step 3). The residue was purified by a chromatography on silica gel column (DCM/MeOH, 100/0 to 99/01) to afford the product (*S*)-**11** as a yellow oil. Yield: 51%. R_f: 0.4.(cyclohexane/EtOAc 7/3). ^{1}H NMR (400 MHz, $CDCl_3$) δ 8.25 (d, ^3J = 8.6 Hz, 1H), 8.15 (d, ^3J = 8.4 Hz, 1H), 7.86-7.81 (m, 1H), 7.74-7.69 (m, 2H), 4.54-4.52 (m, 1H), 3.39 (dd, ^2J = 5.7 Hz, ^3J = 4.2 Hz, 1H), 2.81 (dd, ^2J = 5.8 Hz, ^3J = 2.6 Hz, 1H) ppm. ^{13}C NMR (101 MHz, $CDCl_3$) δ 148.3 (q, ^2J = 34.8 Hz), 146.9, 146.5, 131.1, 130.9, 129.0, 127.2, 121.6 (q, ^1J = 277.2 Hz), 112.4 (q, ^3J = 2.4 Hz), 51.0, 49.4 ppm. IR $ν_{max}$: 3070, 2359, 1723, 1596, 1514, 1353, 1215, 1122, 755 cm^{-1}. HRMS (ESI) *m/z* calculated for $C_{12}H_8F_3NO$ $[M+H]^+$ 240.0636, found 240.0637. Chiral HPLC: 86% ee, Chiralpak IB column, heptane/isopropanol/EDA 99/01/0.1, flow 1.0 mL/min, tr(*S*) = 9.3 min, tr(*R*) = 10.4 min. $[\alpha]_D^{20}$: +57 (c = 0.1, DCM).

(*R*)-4-(oxiran-2-yl)-2-(trifluoromethyl)quinoline 11. (*R*)-1-(2-(trifluoromethyl)quinolin-4-yl)ethane-1,2-diol **15** (231 mg, 0.9 mmol, 1 eq.) was suspended in anhydrous DCM (24 mL). PTSA.H_2O (10 mg, 0.04 mmol, 0.05 eq.) was added then trimethylorthoacetate (344 µL, 2.7 mmol, 3 eq.) was added dropwise according to the procedure D (step 1). TMSBr (412 µL, 2.7 mmol, 3 eq.) was added according to the procedure D (step 2). After concentration under reduced pressure, the solution was solubilized in anhydrous MeOH (24 mL) and K_2CO_3 (198 mg, 1.4 mmol, 1.6 eq.) was added according to the procedure D (step 3). The residue was purified by a chromatography on silica gel column (DCM/MeOH, 100/0 to 99/01) to afford the product (*R*)-**11** as a yellow oil. Yield: 54%. HRMS (ESI) *m/z* calculated for $C_{12}H_8F_3NO$ $[M+H]^+$ 240.0636, found 240.0639. Chiral HPLC: 80% ee, Chiralpak IB column, heptane/isopropanol/EDA 99/01/0.1, flow 1.0 mL/min, tr(*S*) = 9.8 min, tr(*R*) = 11.1 min. $[\alpha]_D^{20}$: -57 (c = 0.1, DCM). R_f, NMR (^{1}H and ^{13}C) and IR were identical to the enantiomer (*S*)-**11**.

4-(oxiran-2-yl)-8-(trifluoromethyl)quinoline 12a. (*S*)-1-(8-(trifluoromethyl)quinolin-4-yl)ethane-1,2-diol **16** (457 mg, 1.8 mmol, 1 eq.) was suspended in anhydrous DCM (46 mL). PTSA.H_2O (16 mg, 0.09 mmol, 0.05 eq.) was added then trimethylorthoacetate (678 µL, 5.3 mmol, 3 eq.) was added dropwise according to the procedure D (step 1). TMSBr (814 µL, 5.3 mmol, 3 eq.) was added according to the procedure D (step 2). After concentration under reduced pressure, the solution was solubilized in anhydrous MeOH (46 mL) and K_2CO_3 (772 mg, 5.6 mmol, 3.2 eq.) was added according to the procedure D (step 3). The organic layers were brought together, dried over Na_2SO_4 and concentrated. The residue was purified by a chromatography on silica gel column (DCM/MeOH, 100/0 to 98/02) to afford the product **12a** as a white solid. Yield: 29%. R_f: 0.5 (cyclohexane/EtOAc 7/3). m.p. 99-101 °C. ^{1}H NMR (400 MHz, $CDCl_3$) δ 9.06 (d, ^3J = 4.4 Hz, 1H), 8.34 (d, ^3J = 7.6 Hz, 1H), 8.13 (d, ^3J = 7.3 Hz, 1H), 7.68 (t, ^3J = 7.9 Hz, 1H), 7.49 (d, ^3J = 4.4 Hz, 1H), 4.51-4.49 (m, 1H), 3.38 (dd, ^2J = 5.8 Hz, ^3J = 4.2 Hz, 1H), 2.80 (dd, ^2J = 5.8 Hz, ^3J = 2.6 Hz, 1H) ppm. ^{13}C NMR (101 MHz, $CDCl_3$) δ 151.5, 144.5, 144.0, 128.8 (q, ^3J = 29.8 Hz), 128.1 (q, ^3J = 5.7 Hz), 127.4, 127.1, 125.7, 124.2 (q, ^1J = 273.4 Hz), 117.4, 50.7, 49.5 ppm. IR ν_{max}: 2924, 1600, 1515, 1312, 1211, 1122, 761 cm^{-1}. HRMS (ESI) *m/z* calculated for $C_{12}H_8F_3NO$ [M+H]$^+$ 240.0636, found 240.0636. Chiral HPLC: 1% ee, Chiralpak IB column, heptane/isopropanol 90/10, flow 1.0 mL/min, tr = 9.7 min, tr = 10.3 min. $[\alpha]_D^{20}$: +3 (c = 0.1, DCM).

4-(oxiran-2-yl)-8-(trifluoromethyl)quinoline 12b. (*R*)-1-(8-(trifluoromethyl)quinolin-4-yl)ethane-1,2-diol **16** (172 mg, 0.7 mmol, 1 eq.) was suspended in anhydrous DCM (18 mL). PTSA.H_2O (7 mg, 0.04 mmol, 0.05 eq.) was added then trimethylorthoacetate (256 µL, 2.0 mmol, 3 eq.) was added dropwise according to the procedure D (step 1). TMSBr (307 µL, 2.0 mmol, 3 eq.) was stirred at 25 °C for 18 h under argon according to the procedure D (step 2). After concentration under reduced pressure, the solution was solubilized in anhydrous MeOH (18 mL) and K_2CO_3 (300 mg, 2.2 mmol, 3.1 eq.) was added according to the procedure D (step 3). The residue was purified by a chromatography on silica gel column (DCM/MeOH, 100/0 to 98/02) to afford the product **12b** as a white solid. Yield: 55%. MS (ESI) *m/z* 240 [M+H]$^+$. Chiral HPLC: 4% ee, Chiralpak IB column, heptane/isopropanol 90/10, flow 1.0 mL/min, tr(*S*) = 9.7 min, tr = 10.3 min. $[\alpha]_D^{20}$: +2 (c = 0.1, DCM). R_f, NMR (^{1}H and ^{13}C) and IR were identical to the enantiomer **12a**.

(*S*)-4-(oxiran-2-yl)-2,8-*bis*(trifluoromethyl)quinoline 13. (*S*)-1-(2,8-*bis*(trifluoromethyl)quinolin-4-yl)ethane-1,2-diol **17** (456 mg, 1.4 mmol, 1 eq.) was suspended in anhydrous DCM (20 mL). PTSA.H_2O (14 mg, 0.07 mmol, 0.05 eq.) was added then, trimethylorthoacetate (536 µL, 4.2 mmol, 3 eq.) was added dropwise according to the procedure D (step 1). TMSBr (554 µL, 4.2 mmol, 3 eq.) was added according to the procedure D (step 2). After concentration under reduced pressure, the solution was solubilized in anhydrous MeOH (20 mL) and K_2CO_3 (290 mg, 2.1 mmol, 1.5 eq.) was added according to the procedure D (step 3). The residue was purified by a chromatography on silica gel column (cyclohexane/Et_2O, 100/0 to 50/50) to afford the product (*S*)-**13** as a white solid. Yield: 51%. R_f: 0.28 (cyclohexane/Et_2O 1/1). m.p. 111-112 °C. ^{1}H NMR (400 MHz, $CDCl_3$) δ 8.38 (d, ^3J = 9.8 Hz, 1H), 8.22 (d, ^3J = 7.2 Hz, 1H), 7.82-7.78 (m, 2H), 4.56-4.54 (m, 1H), 3.42 (dd, ^2J = 5.7 Hz, ^3J = 4.1 Hz, 1H), 2.83 (dd, ^2J = 5.7 Hz, ^3J = 2.6 Hz, 1H) ppm. ^{13}C NMR (101 MHz, $CDCl_3$) δ 148.9 (q, ^2J = 30.7 Hz), 146.8, 143.5, 129.7 (q, ^2J = 30.1 Hz), 129.2 (q, ^3J = 5.5 Hz), 127.7, 127.6, 127.1, 123.6 (q, ^1J = 274.8 Hz), 121.2 (q, ^1J = 276.5 Hz), 113.5 (q, ^3J = 1.7 Hz), 51.0, 49.4 ppm. IR ν_{max}: 2957, 2949, 1606, 1474, 1329, 1307, 1213, 1121, 1103, 772 cm^{-1}. MS (ESI) *m/z* 308 [M+H]$^+$. Chiral HPLC 91% ee, Chiralpak IB column, heptane/isopropanol 95/05, flow 1.0 mL/min, tr(*S*) = 13.5 min, tr(*R*) = 15.9 min. $[\alpha]_D^{20}$ = +46 (c = 0.1, DCM).

(*R*)-4-(oxiran-2-yl)-2,8-*bis*(trifluoromethyl)quinoline 13. (*R*)-1-(2,8-*bis*(trifluoromethyl)quinolin-4-yl)ethane-1,2-diol **17** (438 mg, 1.35 mmol, 1 eq.) was suspended in anhydrous DCM (20 mL). PTSA.H_2O (13 mg, 0.06 mmol, 0.05 eq.) was added then trimethylorthoacetate (512 µL, 3.76 mmol, 2.7 eq.) was added dropwise according to the procedure D (step 1). TMSBr (535 µL, 4.05 mmol, 3 eq.) was added according to the procedure D (step 2). After concentration under reduced pressure, the solution was solubilized in anhydrous MeOH (20 mL) and K_2CO_3 (281 mg, 2.04 mmol, 1.5 eq.) was added according to the procedure D (step 3). The residue was purified by a chromatography on silica gel column (cyclohexane/Et_2O, 100/0 to 50/50) to afford the product (*R*)-**13** as a white solid. Yield: 54%. MS (ESI) *m/z* 308 $[M+H]^+$. Chiral HPLC 93% ee, Chiralpak IB column, heptane/isopropanol 95/05, flow 1.0 mL/min, tr(*S*) = 13.8 min, tr(*R*) = 15.7 min. $[\alpha]_D^{21}$ = -49 (c = 0.1, DCM). R_f, m.p., NMR (^1H and ^{13}C) and IR were identical to the enantiomer (*S*)-**13**.

Synthesis of *N*-alkylpiperazines 5-9.

The synthesis of amino-alcohol-quinolines with an *N*-alkylpiperazine moiety required the alkylation of commercial *tert*-butyl piperazine carboxylate (step a) according to procedure E described here after (Scheme 1). Next, the *tert*-butyl carboxylate group was deprotected in the presence of trifluoroacetic acid (TFA) (step b) according the procedure F and treated with NaOH 1N according the procedure G (step c).

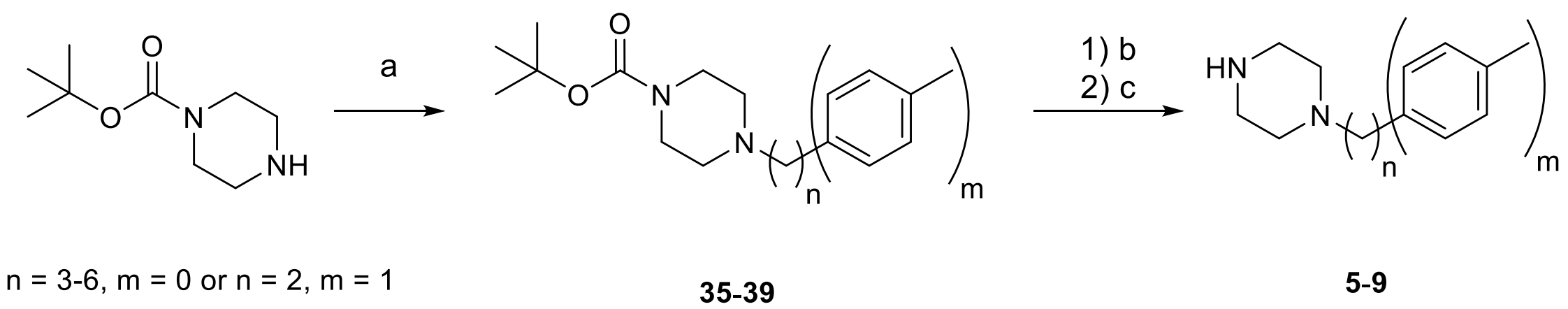

Scheme 1. Synthetic route for *N*-alkylpiperazines **5**-**9**. Reagents and conditions: (a) halogenoalkane derivative, DIPEA, CH_3CN, 0-25 °C. (b) TFA (0.1 M), CH_2Cl_2, 18 °C. (c) NaOH (1N), CH_2Cl_2/CH_3OH (3/1, v/v).

Alkylation of piperazine 35-39.

***Tert*-butyl 4-butylpiperazine-1-carboxylate 35.** To a solution, under argon, of *tert*-butyl piperazine-1-carboxylate (411 mg, 2.2 mmol, 1 eq.) in anhydrous CH_3CN (10 mL) at 0 °C was successively added 1-iodobutane (502 µL, 4.4 mmol, 2 eq.) and DIPEA (600 µL, 3.5 mmol, 1.5 eq.) according to the procedure E. The product was purified by a chromatography on silica gel column (DCM/MeOH, 100/0 to 90/10) to afford the product **35** as a yellow oil. Yield: 42%. R_f: 0.44 (DCM/MeOH 9/1). ^1H NMR (400 MHz, $CDCl_3$) δ 3.61 (t, ^3J = 5.1 Hz, 4H), 2.62 (m, 4H), 2.62-2.52 (m, 2H), 1.66-1.58 (m, 2H), 1.45 (s, 9H), 1.35 (m, 2H), 0.93 (t, ^3J = 7.4 Hz, 3H) ppm. ^{13}C NMR (101 MHz, $CDCl_3$) δ 153.9, 81.6, 57.6 (2C), 51.8 (2C), 29.2, 28.4 (3C), 25.4, 20.2, 13.6 ppm. IR ν_{max}: 2962, 2930, 2875, 1693, 1363, 1269, 959, 865, 765 cm^{-1}. MS (ESI) *m/z* 243 $[M+H]^+$.

***Tert*-butyl 4-pentylpiperazine-1-carboxylate 36.** To a solution, under argon, of *tert*-butyl piperazine-1-carboxylate (605 mg, 3.2 mmol, 1 eq.) in anhydrous CH_3CN (20 mL) at 0 °C was successively added 1-bromopentane (1.07 mL, 5.8 mmol, 1.8 eq.) and DIPEA (838 µL, 3.5 mmol, 1.5 eq.) according to the

procedure E. The product was purified by a chromatography on silica gel column (DCM/MeOH 100/0 to 95/05) to afford the product **36** as a yellow oil. Yield: 39%. R_f: 0.30 (cyclohexane/EtOAc 4/1). ^{1}H NMR (400 MHz, $CDCl_3$) δ 3.43 (t, ^3J = 5.0 Hz, 4H), 2.37 (t, ^3J = 5.1 Hz, 4H), 2.33-2.29 (m, 2H), 1.52-1.45 (m, 2H), 1.45 (s, 9H), 1.30-1.26 (m, 4H), 0.89 (t, ^3J = 7.0 Hz, 3H) ppm. ^{13}C NMR (101 MHz, $CDCl_3$) δ 154.9, 79.7, 59.0 (3C), 53.2 (2C), 29.9, 28.6 (3C), 26.6, 22.7, 14.2 ppm. IR $ν_{max}$: 2955, 2930, 2860, 1695, 1168, 866, 767 cm^{-1}. MS (ESI) *m/z* 257 $[M+H]^+$.

***Tert*-butyl 4-hexylpiperazine-1-carboxylate 37.** To a solution, under argon, of *tert*-butyl piperazine-1-carboxylate (600 mg, 3.2 mmol, 1 eq.) in anhydrous CH_3CN (50 mL) at 0 °C were successively added 1-bromohexane (898 µL, 6.4 mmol, 2 eq.) and DIPEA (800 µL, 4.7 mmol, 1.5 eq.) according to the procedure E. The product was purified by a chromatography on silica gel column (cyclohexane/EtOAc 100/0 to 80/20) to afford the product **37** as a white solid. Yield: 68%. R_f: 0.26 (cyclohexane/EtOAc 4/1). m.p. 197-199°C. ^{1}H NMR (400 MHz, $CDCl_3$) δ 4.20-4.12 (m, 2H), 3.78-3.64 (m, 2H), 3.51-3.48 (m, 2H), 2.96-2.92 (m, 2H), 2.75-2.66 (m, 2H), 1.92-1.84 (m, 2H), 1.43 (s, 9H), 1.36-1.26 (m, 6H), 0.84-0.87 (m, 3H) ppm. ^{13}C NMR (101 MHz, $CDCl_3$) δ 153.9, 81.5, 57.9 (2C), 51.9 (3C), 31.2, 28.4 (3C), 26.6, 23.4, 22.5, 14.0 ppm. IR $ν_{max}$: 2954, 2929, 2858, 1695, 1364, 1168, 866, 767 cm^{-1}. MS (ESI) *m/z* 271 $[M+H]^+$.

***Tert*-butyl 4-heptylpiperazine-1-carboxylate 38.** To a solution, under argon, of *tert*-butyl piperazine-1-carboxylate (401 mg, 2.1 mmol, 1 eq.) in anhydrous CH_3CN (10 mL) at 0 °C were successively added 1-bromoheptane (675 µL, 4.3 mmol, 2 eq.) and DIPEA (550 µL, 3.2 mmol, 1.5 eq.) according to the procedure E. The product was purified by a chromatography on silica gel column (cyclohexane/EtOAc 100/0 to 70/30) to afford the product **38** as a white solid. Yield: 71%. R_f: 0.51 (cyclohexane/EtOAc 4/1). m.p. 200-202°C. ^{1}H NMR (400 MHz, CD_3OD) δ 3.78-3.58 (m, 3H), 3.24-3.12 (m, 3H), 3.08-3.04 (m, 2H), 1.76-1.72 (m, 2H), 1.48 (s, 9H), 1.40-1.32 (m, 8H), 0.93-0.90 (m, 3H) ppm. ^{13}C NMR (101 MHz, CD_3OD) δ 155.5, 82.3, 58.4, 52.8 (4C), 32.7, 29.9, 28.5 (3C), 27.7, 25.3, 23.6, 14.4 ppm. IR $ν_{max}$: 2923, 2855, 1692, 1363, 1279, 967, 866, 765 cm^{-1}. MS (ESI) *m/z* 285 $[M+H]^+$.

***Tert*-butyl 4-(4-methylphenethyl)piperazine-1-carboxylate 39.** To a solution, under argon, of *tert*-butyl piperazine-1-carboxylate (600 mg, 3.2 mmol, 1 eq.) in anhydrous CH_3CN (38 mL) were successively added 1-(2-bromoethyl)-4-methylbenzene (1.08 mL, 7.1 mmol, 2 eq.) and DIPEA (800 µL, 4.7 mmol, 1.5 eq.) according to the procedure E. The product was purified by a chromatography on silica gel column (cyclohexane/EtOAc 100/0 to 60/40) to afford the product **39** as a yellow oil. Yield: 41%. R_f: 0.34 (cyclohexane/EtOAc 1/1). ^{1}H NMR (400 MHz, $CDCl_3$) δ 7.26 (s, 4H), 3.64 (t, ^3J = 5.1 Hz, 4H), 2.95-2.91 (m, 2H), 2.76-2.72 (m, 2H), 2.61 (t, ^3J = 5.0 Hz, 4H), 2.49 (s, 3H), 1.67 (s, 9H) ppm. ^{13}C NMR (101 MHz, $CDCl_3$) δ 154.1, 135.8, 134.5, 128.0 (2C), 127.4 (2C), 79.1, 59.4, 51.8 (2C), 46.9 (2C), 31.5, 26.6 (3C), 19.1 ppm. IR $ν_{max}$: 3006, 2930, 1682, 1363, 1247, 1168, 1120 cm^{-1}. HRMS (ESI) *m/z* calculated for $C_{18}H_{29}N_2O_2$ $[M+H]^+$ 305.2229, found 305.2226.

Deprotection of the boc group 40-44.

4-butylpiperazin-1-ium 2,2,2-trifluoroacetate salt 40. To a solution of *tert*-butyl 4-butylpiperazine-1-carboxylate **35** (208 mg, 0.86 mmol, 1 eq.) in anhydrous DCM (7 mL) was added TFA (1.5 mL) according to the procedure F. The TFA was removed under reduced pressure to afford the product **40** as a yellow solid. Yield: 97%. R_f: 0.67 (DCM/MeOH 9/1 + 0,2 NH_3). m.p. 129-131°C. ^{1}H NMR (400 MHz, CD_3OD) δ 3.55-3.52 (m, 4H), 3.48-3.40 (m, 4H), 3.14-3.10 (m, 2H), 1.76-1.68 (m, 2H), 1.46-1.41 (m, 2H), 1.38-1.35 (m, 2H), 1.00 (t, ^3J = 7.4 Hz, 3H) ppm. ^{13}C NMR (101 MHz, CD_3OD) δ 58.3, 49.9 (2C), 42.5 (2C), 27.3, 20.9, 13.9 ppm. IR ν_{max}: 2972, 2942, 2469, 1665, 1182, 1163, 1133, 719 cm^{-1}. MS (ESI) *m/z* 143 [M+H]$^+$ (corresponding to the base).

4-pentylpiperazin-1-ium 2,2,2-trifluoroacetate salt 41. To a solution of *tert*-butyl 4-pentylpiperazine-1-carboxylate **36** (88 mg, 0.34 mmol, 1 eq.) in anhydrous DCM (3 mL) was added TFA (1 mL) according to the procedure F. The TFA was removed under reduced pressure to afford the product **41** as a white solid. Yield: quantitative. R_f: 0.78 (DCM/MeOH 98/2). m.p. 129-131°C. ^{1}H NMR (400 MHz, CD_3OD) δ 3.51 (t, ^3J = 5.3 Hz, 4H), 3.44-3.36 (m, 4H), 3.09-3.05 (m, 2H), 1.76-1.68 (m, 2H), 1.43-1.35 (m, 4H), 0.95 (t, ^3J = 6.8 Hz, 3H) ppm. ^{13}C NMR (101 MHz, CD_3OD) δ 58.5, 50.0 (2C), 42.6 (2C), 29.7, 25.1, 23.2, 14.1 ppm. IR ν_{max}: 2963, 2942, 2875, 1661, 1184, 1161, 1135, 717 cm^{-1}. MS (ESI) *m/z* 157 [M+H]$^+$ (corresponding to the base).

4-hexylpiperazin-1-ium 2,2,2-trifluoroacetate salt 42. To a solution of *tert*-butyl 4-hexylpiperazine-1-carboxylate **37** (108 mg, 0.4 mmol, 1 eq.) in anhydrous DCM (4 mL) was added TFA (1 mL) according to the procedure F. The TFA was removed under reduced pressure to afford the product **42** as a white solid. Yield: quantitative. R_f: 0.67 (DCM/MeOH 9/1 + 0.2 NH_3). m.p. 131-133°C. ^{1}H NMR (400 MHz, CD_3OD): 3.62-3.55 (m, 7H), 3.24-3.20 (m, 2H), 1.89-1.86 (m, 1H), 1.77-1.72 (m, 2H), 1.42-1.34 (m, 6H), 0.94-0.91 (m, 3H) ppm. IR ν_{max}: 3419, 1667, 1455, 1434, 1175, 1132, 722 cm^{-1}. MS (ESI) *m/z* 171 [M+H]$^+$ (corresponding to the base).

4-heptylpiperazin-1-ium 2,2,2-trifluoroacetate salt 43. To a solution of *tert*-butyl 4-heptylpiperazine-1-carboxylate **38** (171 mg, 0.6 mmol, 1 eq.) in anhydrous DCM (5 mL) was added TFA (1 mL) according to the procedure F. The TFA was removed under reduced pressure to afford the product **43** as a white solid. Yield: 99%. R_f: 0.5 (DCM/MeOH 9/1 + 0.2 NH_3). m.p. 130-132 °C. ^{1}H NMR (400 MHz, CD_3OD) δ 3.51 (t, ^3J = 5.3 Hz, 4H), 3.66-3.56 (m, 4H), 3.09-3.05 (m, 2H), 1.74-1.70 (m, 2H), 1.41-1.31 (m, 8H), 0.91 (t, ^3J = 6.8 Hz, 3H) ppm. ^{13}C NMR (101 MHz, CD_3OD) δ 58.3, 49.6 (2C), 41.9 (2C), 32.6, 29.7, 27.3, 24.7, 23.5, 14.3 ppm. IR ν_{max}: 2955, 2931, 2861, 1661, 1460, 1428, 1413, 718 cm^{-1}. MS (ESI) *m/z* 185 [M+H]$^+$ (corresponding to the base).

1-(4-methylphenethyl)piperazine, 2,2,2-trifluoroacetate salt 44. To a solution of *tert*-butyl 4-(4-methylphenethyl)piperazine-1-carboxylate **39** (691 mg, 2.3 mmol, 1 eq.) in anhydrous DCM (15 mL) was added TFA (3 mL) according to the procedure F. The TFA was removed under reduced pressure to afford the product **44** as a white solid. Yield: quantitative. R_f: 0.36 (DCM/MeOH 9/1 + 0.2 NH_3). m.p. 165-167 °C. ^{1}H NMR (400 MHz, CD_3OD) δ 7.19-7.14 (m, 4H), 3.60 (m, 8H), 3.43-3.39 (m, 2H), 3.09-3.01 (m, 2H), 2.31 (s, 3H) ppm. ^{13}C NMR (101 MHz, CD_3OD) δ 138.2, 134.1, 130.6 (2C), 129.7 (2C), 59.3, 49.8 (2C), 42.1 (2C), 30.8, 21.1 ppm. IR ν_{max}: 3033, 1665, 1388, 1169, 1123, 719 cm^{-1}. MS (ESI) *m/z* 205 [M+H]$^+$ (corresponding to the base).

Basic treatment 5-9.

1-butylpiperazine 5. To a solution of 4-butylpiperazin-1-ium 2,2,2-trifluoroacetate salt **40** (214 mg, 0.84 mmol, 1 eq.) in mixture of DCM/MeOH 3/1 was added NaOH 1N until a pH of 12-14 according to the procedure G. The product was obtained as an orange oil. Yield: 30%. R_f: 0.67 (DCM/MeOH 9/1 + 0.2 NH_3). ^1H NMR (400 MHz, $CDCl_3$) δ 2.89 (t, ^3J = 4.8 Hz, 4H), 2.45-2.35 (m, 4H), 2.30-2.26 (m, 2H), 1.47-1.40 (m, 2H), 1.30-1.22 (m, 2H), 0.88 (t, ^3J = 7.3 Hz, 3H) ppm. ^{13}C NMR (101 MHz, $CDCl_3$) δ 59.0, 54.0 (2C), 45.7 (2C), 28.8, 20.8, 14.1 ppm. IR ν_{max}: 2972, 2942, 2469, 1665, 1182, 1163, 1133, 719 cm^{-1}. MS (ESI) *m/z* 143 $[M+H]^+$.

1-pentylpiperazine 6. To a solution of 4-pentylpiperazin-1-ium 2,2,2-trifluoroacetate salt **41** (136 mg, 0.34 mmol, 1 eq.) in mixture of DCM/MeOH 3/1 was added NaOH 1N until a pH of 12-14 according to the procedure G. The product was obtained as an orange oil. Yield: 75%. R_f: 0.78 (DCM/ MeOH 98/2). ^1H NMR (400 MHz, $CDCl_3$) δ 2.89 (t, ^3J = 4.9 Hz, 4H), 2.40-2.37 (m, 5H), 2.30-2.27 (m, 2H), 1.47 (ddd, ^3J = 15.1 Hz, ^3J = 8.0 Hz, ^3J = 6.3 Hz, 2H), 1.32-1.22 (m, 5H), 0.87 (t, ^3J = 7.1 Hz, 3H) ppm. ^{13}C NMR (101 MHz, $CDCl_3$) δ 58.7, 52.9 (2C), 44.5 (2C), 29.7, 26.4, 22.6, 14.0. IR ν_{max}: 2963, 2942, 2875, 1661, 1184, 1161, 1135, 717 cm^{-1}. MS (ESI) *m/z* 157 $[M+H]^+$.

1-hexylpiperazine 7. To a solution of 4-hexylpiperazin-1-ium 2,2,2-trifluoroacetate salt **42** (222 mg, 0.78 mmol, 1 eq.) in mixture of DCM/MeOH 3/1 was added NaOH 1N until a pH of 12-14 according to the procedure G. The product was obtained as a yellow oil. Yield: 96%. R_f: 0.67 (DCM/MeOH 9/1 + 0.2 NH_3). ^1H NMR (400 MHz, $CDCl_3$) δ 2.87 (t, ^3J = 4.9 Hz, 4H), 2.40-2.30 (m, 4H), 2.29-2.25 (m, 2H), 1.96 (s, 1H, NH), 1.49-1.41 (m, 2H), 1.28-1.23 (m, 6H), 0.85 (t, ^3J = 6.0 Hz, 3H) ppm. ^{13}C NMR (101 MHz, $CDCl_3$) δ 59.4, 53.9 (2C), 45.7 (2C), 31.9, 29.9, 27.4, 26.6, 22.7, 14.2 ppm. IR ν_{max}: 3419, 1667, 1455, 1434, 1175, 1132, 722 cm^{-1}. MS (ESI) *m/z* 170 $[M+H]^+$.

1-heptylpiperazine 8. To a solution of 4-heptylpiperazin-1-ium 2,2,2-trifluoroacetate salt **43** (177 mg, 0.59 mmol, 1 eq.) in mixture of DCM/MeOH 3/1 was added NaOH 1N until a pH of 12-14 according to the procedure G. The product was obtained as an orange oil. Yield: 46%. R_f: 0.50 (DCM/MeOH 9/1 + 0.2 NH_3). ^1H NMR (400 MHz, $CDCl_3$) δ 2.83 (t, ^3J = 4.9 Hz, 4H), 2.40-2.37 (m, 4H), 2.27-2.23 (m, 2H), 1.44-1.40 (m, 2H), 1.24-1.20 (m, 8H), 0.79 (t, ^3J = 7.2 Hz, 3H) ppm. ^{13}C NMR (101 MHz, $CDCl_3$) δ 59.51, 54.35 (2C), 45.92 (2C), 31.93, 29.37, 27.70, 26.73, 22.74, 14.21 ppm. IR ν_{max}: 2955, 2931, 2861, 1661, 1460, 1428, 1413, 718 cm^{-1}. MS (ESI) *m/z* 185 $[M+H]^+$.

1-(4-methylphenethyl)piperazine 9. 1-(4-methylphenethyl)piperazine, 2,2,2-trifluoroacetate salt **44** (810 mg, 2.3 mmol, 1 eq.) in mixture of DCM/H_2O 3/1 (16 mL) was added NaOH 1N until a pH of 12-14 according to the procedure G. The product was obtained as an orange solid. Yield: 99%. R_f: 0.52 (DCM/MeOH 9.8/0.2). m.p. 125-127 °C. ^1H NMR (400 MHz, $CDCl_3$) δ 7.04 (s, 4H), 2.86 (t, ^3J = 4.9 Hz, 4H), 2.73-2.69 (m, 2H), 2.53-2.48 (m, 2H), 2.44-2.40 (m, 5H), 2.26 (s, 3H) ppm. ^{13}C NMR (101 MHz, $CDCl_3$) δ 137.1, 135.2, 128.9 (2C), 128.4 (2C), 61.1, 54.2 (2C), 45.8 (2C), 32.7, 20.8 ppm. IR ν_{max}: 3414, 2932, 2801, 1514, 1252, 1122, 991, 793 cm^{-1}. HRMS (ESI) *m/z* calculated for $C_{13}H_{21}N_2$ $[M+H]^+$ 205.1705, found 205.1707.

Final products (*S*/*R*)-1-4a-f.

(*S*)-2-(pentylamino)-1-(quinolin-4-yl)ethan-1-ol 1. To a solution of pentan-1-amine (92 µL, 0.8 mmol, 4 eq.) in EtOH (1 mL) was added (*S*)-4-(oxiran-2-yl)quinoline **10** (32 mg, 0.2 mmol, 1 eq.) according to the procedure H. The product was concentrated *in vacuo* and purified by a chromatography on silica gel column (DCM/methanol 100/0 to 90/10) to afford the product (*S*)-**1** as a yellow oil. Yield: 25%. R_f: 0.24 (cyclohexane/EtOAc 3/2). ^{1}H NMR (400 MHz, $CDCl_3$) δ 8.91 (d, ^3J = 4.4 Hz, 1H), 8.14 (d, ^3J = 8.4 Hz, 1H), 7.97 (d, ^3J = 8.4 Hz, 1H), 7.71 (ddd, ^3J = 8.4 Hz, ^3J = 6.8 Hz, ^4J = 1.4 Hz, 1H), 7.67-7.66 (m, 1H), 7.55 (ddd, ^3J = 8.4 Hz, ^3J = 6.8 Hz, ^4J = 1.3 Hz, 1H), 5.48 (dd, ^3J = 8.5 Hz, ^3J = 3.6 Hz, 1H), 3.17 (dd, ^2J = 12.5 Hz, ^3J = 3.7 Hz, 1H), 2.71 (m, 3H), 1.50 (m, 2H), 1.32 (m, 4H), 0.90 (m, 3H) ppm. ^{13}C NMR (101 MHz, $CDCl_3$) δ 150.5, 148.2 (2C), 130.4, 129.0, 126.5, 125.6, 122.7, 117.6, 67.4, 55.6, 49.5, 29.9, 29.4, 22.6, 14.0 ppm. IR ν_{max}: 2925, 2854, 1591, 1343, 1113, 1094, 756 cm^{-1}. HRMS (ESI) *m/z* calculated for $C_{16}H_{23}N_2O$ $[M+H]^+$ 259.1810, found 259.1802. Chiral HPLC: ee not applicable. $[\alpha]_D^{T°C}$: not applicable.

(*R*)-2-(pentylamino)-1-(quinolin-4-yl)ethan-1-ol 1. To a solution of pentan-1-amine (155 µL, 1.3 mmol, 4 eq.) in EtOH (1 mL) was added (*R*)-4-(oxiran-2-yl)quinoline **10** (54 mg, 0.3 mmol, 1 eq.) according to the procedure H. The product was concentrated *in vacuo* and purified by a chromatography on silica gel column (DCM/methanol 100/0 to 90/10) to afford the product (*R*)-**1** as a yellow oil. Corrected yield: 17%. MS (ESI) *m/z* 259 $[M+H]^+$. Chiral HPLC: ee not applicable. $[\alpha]_D^{T°C}$: not applicable. R_f, NMR (^{1}H and ^{13}C) and IR were identical to the enantiomer (*S*)-**1**.

(*S*)-2-(pentylamino)-1-(2-(trifluoromethyl)quinolin-4-yl)ethan-1-ol 2. To a solution of pentan-1-amine (110 µL, 1.0 mmol, 3 eq.) in EtOH (2 mL) was added (*S*)-4-(oxiran-2-yl)-2-(trifluoromethyl)quinoline **11** (77 mg, 0.3 mmol, 1 eq.) according to the procedure H. The product was concentrated *in vacuo* and purified by a chromatography on silica gel column (cyclohexane/EtOAc 100/0 to 0/100) to afford the product (*S*)-**2** as a white solid. Yield: 63%. R_f: 0.32 (cyclohexane/EtOAc 3/2). m.p. 121-123 °C. ^{1}H NMR (400 MHz, $CDCl_3$) δ 8.23 (d, ^3J = 8.5 Hz, 1H), 8.04 (s, 1H), 7.98 (d, ^3J = 8.5 Hz, 1H), 7.80-7.76 (m, 1H), 7.66-7.62 (m, 1H), 5.50 (dd, ^3J = 8.9 Hz, ^3J = 3.5 Hz, 1H), 3.17 (dd, ^2J = 12.5 Hz, ^3J = 3.6 Hz, 1H), 2.76-2.70 (m, 2H), 2.66-2.60 (m, 2H), 1.52-1.49 (m, 2H), 1.33-1.29 (m, 4H), 0.91-0.88 (m, 3H) ppm. ^{13}C NMR (101 MHz, $CDCl_3$) δ 151.3, 148.5 (q, ^2J = 35.2 Hz), 147.34, 131.3, 130.4, 128.6, 126.3, 122.8, 121.8 (q, ^1J = 275.6 Hz), 113.6, 67.3, 55.6, 49.6, 29.9, 29.5, 22.7, 14.2 ppm. IR ν_{max}: 2927, 2862, 1598, 1363, 1128, 1094, 760 cm^{-1}. HRMS (ESI) *m/z* calculated for $C_{17}H_{22}F_3N_2O$ $[M+H]^+$ 327.1684, found 327.1686. Chiral HPLC 84% ee, Chiralpak IG column, heptane/isopropanol/EDA 99/01/0.1, flow 1.0 mL/min, tr(*S*) = 7.1 min, tr(*R*) = 12.0 min. $[\alpha]_D^{22}$: +133 (c = 0.1, DCM).

(*R*)-2-(pentylamino)-1-(2-(trifluoromethyl)quinolin-4-yl)ethan-1-ol 2. To a solution of pentan-1-amine (110 µL, 1.0 mmol, 3 eq.) in EtOH (2 mL) was added (*R*)-4-(oxiran-2-yl)-2-(trifluoromethyl)quinoline **11** (76 mg, 0.3 mmol, 1 eq.) according to the procedure H. The product was concentrated *in vacuo* and purified by a chromatography on silica gel column (cyclohexane/EtOAc 100/0 to 0/100) to afford the product (*R*)-**2** as a white solid. Yield: 56%. HRMS (ESI) *m/z* calculated for $C_{17}H_{22}F_3N_2O$ $[M+H]^+$ 327.1684, found 327.1682. Chiral HPLC 93% ee, Chiralpak IG column, heptane/isopropanol/EDA 99/01/0.1, flow 1.0 mL/min, tr(*S*) = 7.4 min, tr(*R*) = 12.6 min. $[\alpha]_D^{22}$: -125 (c = 0.1, DCM). R_f, m.p., NMR (^{1}H and ^{13}C) and IR were identical to the enantiomer (*S*)-**2**.

2-(pentylamino)-1-(8-(trifluoromethyl)quinolin-4-yl)ethan-1-ol 3a. To a solution of pentan-1-amine (117 µL, 1.0 mmol, 3 eq.) in MeOH (2 mL) was added 4-(oxiran-2-yl)-8-(trifluoromethyl)quinoline **12a** (80 mg, 0.3 mmol, 1 eq.) according to the procedure H. The product was concentrated *in vacuo* and

purified by a chromatography on silica gel column (cyclohexane/EtOAc 100/0 to 0/100) to afford the product **3a** as a white solid. Yield: 69%. R_f: 0.40 (DCM/MeOH 9.5/0.5). m.p. 95-97 °C. ^{1}H NMR (400 MHz, $CDCl_3$) δ 9.07 (d, ^3J = 4.5 Hz, 1H), 8.19 (d, ^3J = 8.8 Hz, 1H), 8.08 (d, ^3J = 6.8 Hz, 1H), 7.78 (d, ^3J = 4.3 Hz, 1H), 7.59 (t, ^3J = 7.9 Hz, 1H), 5.50 (dd, ^3J = 8.9 Hz, ^3J = 3.5 Hz, 1H), 3.11 (dd, ^2J = 12.4 Hz, ^3J = 3.5 Hz, 1H), 1.51-1.48 (m, 2H), 1.35-1.22 (m, 5H), 0.91-0.85 (m, 3H) ppm. ^{13}C NMR (101 MHz, $CDCl_3$) δ 151.2, 148.7, 144.7, 128.5 (q, ^2J = 29.0 Hz), 127.6 (q, ^3J = 5.7 Hz), 127.2, 126.1, 125.1, 124.1 (q, ^1J = 273.4 Hz), 118.5, 67.5, 55.7, 49.5, 29.7, 29.3, 22.5, 14.0 ppm. IR ν_{max}: 2927, 2862, 1598, 1363, 1128, 1094, 760 cm^{-1}. HRMS (ESI) *m/z* calculated for $C_{17}H_{22}F_3N_2O$ $[M+H]^+$ 327.1684, found 327.1687. Chiral HPLC 8% ee, Chiralpak IG column, heptane/isopropanol/EDA 95/05/0.1, flow 1.0 mL/min, tr = 14.0 min, tr = 21.6 min. $[\alpha]_D^{21}$: +2 (c = 0.1, DCM).

2-(pentylamino)-1-(8-(trifluoromethyl)quinolin-4-yl)ethan-1-ol 3b. To a solution of pentan-1-amine (117 µL, 1.0 mmol, 3 eq.) in MeOH (2 mL) was added 4-(oxiran-2-yl)-8-(trifluoromethyl)quinoline **12b** (81 mg, 0.3 mmol, 1 eq.) according to the procedure H. The product was concentrated *in vacuo* and purified by a chromatography on silica gel column (cyclohexane/EtOAc 100/0 to 0/100) to afford the product **3b** as a white solid. Yield: 55%. HRMS (ESI) *m/z* calculated for $C_{17}H_{22}F_3N_2O$ $[M+H]^+$ 327.1684, found 327.1683. Chiral HPLC 1% ee, Chiralpak IG column, heptane/isopropanol/EDA 95/05/0.1, flow 1.0 mL/min, tr = 12.3 min, tr = 23.4 min. $[\alpha]_D^{21}$: -1 (c = 0.1, DCM). R_f, m.p., NMR (^{1}H and ^{13}C) and IR were identical to the enantiomer **3a**.

(*S*)-1-(2,8-*bis*(trifluoromethyl)quinolin-4-yl)-2-(4-butylpiperazin-1-yl)ethan-1-ol 4a. To a solution of (*S*)-4-(oxiran-2-yl)-2,8-*bis*(trifluoromethyl)quinoline **13** (31 mg, 0.09 mmol, 1 eq.) in EtOH (1 mL) was added 1-butylpiperazine **5** (42 mg, 0.3 mmol, 3 eq.) according to the procedure H. The product was concentrated *in vacuo* and purified by a chromatography on silica gel column (DCM/MeOH 100/0 to 70/30) to afford the product **4a** as a yellow oil. Yield: 50%. R_f: 0.29 (DCM/MeOH 9/1). ^{1}H NMR (400 MHz, $CDCl_3$) δ 8.19-8.15 (m, 3H), 7.72 (t, ^3J = 7.7 Hz, 1H), 5.55 (dd, ^3J = 10.6 Hz, ^3J = 2.6 Hz, 1H), 2.89 (s, 1H, OH), 2.85 (dd, ^2J = 12.7 Hz, ^3J = 3.4 Hz, 2H), 2.62-2.58 (m, 4H), 2.49 (dd, ^2J = 12.7 Hz, ^3J = 10.6 Hz, 2H), 2.43-2.38 (m, 2H), 1.54-1.47 (m, 2H), 1.39-1.30 (m, 2H), 0.90 (t, ^3J = 7.3 Hz, 3H) ppm. ^{13}C NMR (101 MHz, $CDCl_3$) δ 151.1, 148.7 (q, ^2J = 35.2 Hz), 143.6, 129.6 (q, ^2J = 29.9 Hz), 128.7 (q, ^3J = 5.5 Hz), 127.1, 126.8, 126.6, 123.5 (q, ^1J = 273.8 Hz), 121.2 (q, ^1J = 275.8 Hz), 114.5 (q, ^3J = 2.1 Hz), 65.1, 64.5, 58.3, 53.1 (4C), 28.9, 20.7, 14.0 ppm. IR ν_{max}: 2822, 1428, 1373, 1306, 1137, 1105, 767 cm^{-1}. HRMS (ESI) *m/z* calculated for $C_{21}H_{26}F_6N_3O$ $[M+H]^+$ 450.1980, found 450.1989. Chiral HPLC 91% ee, Chiralpak IG column, heptane/isopropanol 99/01, flow 1.0 mL/min, tr(*S*) = 15.9 min, tr(*R*) = 23.0 min. $[\alpha]_D^{21}$ = +57 (c = 0.1, DCM).

(*R*)-1-(2,8-*bis*(trifluoromethyl)quinolin-4-yl)-2-(4-butylpiperazin-1-yl)ethan-1-ol 4a. To a solution of (*R*)-4-(oxiran-2-yl)-2,8-*bis*(trifluoromethyl)quinoline **13** (60 mg, 0.2 mmol, 1 eq.) in EtOH (1 mL) was added 1-butylpiperazine **5** (49 mg, 0.3 mmol, 1.5 eq.) according to the procedure H. The product was concentrated *in vacuo* and purified by a chromatography on silica gel column (DCM/MeOH 100/0 to 70/30) to afford the product **4a** as a white oil. Yield: 40%. HRMS (ESI) *m/z* calculated for $C_{21}H_{26}F_6N_3O$ $[M+H]^+$ 450.1980, found 450.1983. Chiral HPLC 89% ee, Chiralpak IG column, heptane/isopropanol 99/01, flow 1.0 mL/min, tr(*S*) = 17.1 min, tr(*R*) = 20.4 min. $[\alpha]_D^{21}$ = -63 (c = 0.1, DCM). R_f, NMR (^{1}H and ^{13}C) and IR were identical to the enantiomer (*S*)-**4a**.

(*S*)-1-(2,8-*bis*(trifluoromethyl)quinolin-4-yl)-2-(4-pentylpiperazin-1-yl)ethan-1-ol 4b. To a solution of (*S*)-4-(oxiran-2-yl)-2,8-*bis*(trifluoromethyl)quinoline **13** (60 mg, 0.2 mmol, 1 eq.) in EtOH (1 mL) was added 1-pentylpiperazine **6** (94 mg, 0.6 mmol, 3 eq.) according to the procedure H. The product was concentrated *in vacuo* and purified by a chromatography on silica gel column (DCM/MeOH 100/0 to

70/30) to afford the product **4b** as a yellow oil. Yield: 80%. *R*f: 0.65 (DCM/MeOH 9.8/0.2). ^{1}H NMR (400 MHz, $CDCl_3$) δ 8.19-8.14 (m, 3H), 7.72 (t, ^{3}J = 7.9 Hz, 1H), 5.55 (dd, ^{3}J = 10.5 Hz, ^{3}J = 3.3 Hz, 1H), 2.88 (s, 1H, OH), 2.85 (dd, ^{2}J = 12.7 Hz, ^{3}J = 3.4 Hz, 2H), 2.60-2.58 (m, 5H), 2.49 (dd, ^{2}J = 12.7 Hz, ^{3}J = 10.6 Hz, 2H), 2.39-2.35 (m, 2H), 1.54-1.47 (m, 2H), 1.37-1.24 (m, 4H), 0.89 (t, ^{3}J = 7.3 Hz, 3H) ppm. ^{13}C NMR (101 MHz, $CDCl_3$) δ 151.3, 149.0 (q, ^{2}J = 35.2 Hz), 143.6, 129.6 (q, ^{2}J = 29.9 Hz), 128.7 (q, ^{3}J = 5.5 Hz), 127.1, 126.8, 126.6, 123.5 (q, ^{1}J = 273.8 Hz), 121.2 (q, ^{1}J = 275.8 Hz), 114.5 (q, ^{3}J = 2.1 Hz), 65.1, 64.5, 58.3, 53.1 (4C), 28.9, 20.7, 14.0 ppm. IR ν_{max}: 2961, 2814, 1379, 1302, 1147, 1107, 771 cm^{-1}. HRMS (ESI) *m/z* calculated for $C_{22}H_{28}F_6N_3O$ $[M+H]^+$ 464.2137, found 464.2147. Chiral HPLC 87% ee, Chiralpak IG column, heptane/isopropanol 99/01, flow 1.0 mL/min, tr(*S*) = 15.1 min, tr(*R*) = 19.8 min. $[\alpha]_D^{21}$ = +69 (c = 0.1, DCM).

(*R*)-1-(2,8-*bis*(trifluoromethyl)quinolin-4-yl)-2-(4-pentylpiperazin-1-yl)ethan-1-ol 4b. To a solution of (*R*)-4-(oxiran-2-yl)-2,8-*bis*(trifluoromethyl)quinoline **13** (30 mg, 0.1 mmol, 1 eq.) in EtOH (1.5 mL) was added 1-pentylpiperazine **6** (39 mg, 0.25 mmol, 2.5 eq.) according to the procedure H. The product was concentrated *in vacuo* and purified by a chromatography on silica gel column (DCM/MeOH 100/0 to 70/30) to afford the product **4b** as a yellow oil. Yield: 67%. HRMS (ESI) *m/z* calculated for $C_{22}H_{28}F_6N_3O$ $[M+H]^+$ 464.2137, found 464.2140. Chiral HPLC 86% ee, Chiralpak IG column, heptane/isopropanol 99/01, flow 1.0 mL/min, tr(*S*) = 15.9 min, tr(*R*) = 18.8 min. $[\alpha]_D^{21}$ = -48 (c = 0.1, DCM). R_f, NMR (^{1}H and ^{13}C) and IR were identical to the enantiomer (*S*)-**4b**.

(*S*)-1-(2,8-*bis*(trifluoromethyl)quinolin-4-yl)-2-(4-hexylpiperazin-1-yl)ethan-1-ol 4c. To a solution of (*S*)-4-(oxiran-2-yl)-2,8-*bis*(trifluoromethyl)quinoline **26** (89 mg, 0.3 mmol, 1 eq.) in EtOH (3.2 mL) was added 1-hexylpiperazine **7** (120 mg, 0.7 mmol, 2.4 eq.) according to the procedure H. The product was concentrated *in vacuo* and purified by a chromatography on silica gel column (DCM/MeOH 100/0 to 70/30) to afford the product **4c** as a pink oil. Yield: 59%. *R*f: 0.71 (DCM/MeOH 9.8/0.2). ^{1}H NMR (400 MHz, $CDCl_3$) δ 8.19-8.14 (m, 3H), 7.72 (t, ^{3}J = 7.3 Hz, 1H), 5.55 (dd, ^{3}J = 10.4 Hz, ^{3}J = 3.1 Hz, 1H), 2.88-2.82 (m, 3H), 2.60-2.46 (m, 7H), 2.39-2.35 (m, 2H), 1.52-1.48 (m, 2H), 1.33-1.29 (m, 6H), 0.91-0.87 (m, 3H) ppm. ^{13}C NMR (101 MHz, $CDCl_3$) δ 151.3, 148.8 (q, ^{2}J = 34.9 Hz), 143.8, 129.7 (q, ^{2}J = 30.1 Hz), 128.8 (q, ^{3}J = 5.4 Hz), 127.2, 126.9, 126.7, 123.6 (q, ^{1}J = 273.6 Hz), 121.4 (q, ^{1}J = 272.3 Hz), 114.6 (q, ^{3}J = 2.3 Hz), 65.2, 64.7, 58.8 (4C), 53.3, 31.9, 27.4, 27.0, 22.7, 14.2 ppm. IR ν_{max}: 2833, 1674, 1308, 1138, 1106, 768 cm^{-1}. HRMS (ESI) *m/z* calculated for $C_{23}H_{30}F_6N_3O$ $[M+H]^+$ 478.2293, found 478.2294. Chiral HPLC = 97% ee, Chiralpak IG column, heptane/isopropanol 99/01, flow 1.0 mL/min, tr(*S*) = 15.9 min. tr(*R*) = 37.7 min. $[\alpha]_D^{20}$ = +79 (c = 0.1, DCM).

(*R*)-1-(2,8-*bis*(trifluoromethyl)quinolin-4-yl)-2-(4-hexylpiperazin-1-yl)ethan-1-ol 4c. To a solution of (*R*)-4-(oxiran-2-yl)-2,8-*bis*(trifluoromethyl)quinoline **13** (234 mg, 0.75 mmol, 1 eq.) in EtOH (12 mL) was added 1-hexylpiperazine **7** (346 mg, 2.0 mmol, 2.7 eq.) according to the procedure H. The product was concentrated *in vacuo* and purified by a chromatography on silica gel column (DCM/MeOH 100/0 to 70/30) to afford the product **4c** as a yellow oil. Yield: 63%. HRMS (ESI) *m/z* calculated for $C_{23}H_{30}F_6N_3O$ $[M+H]^+$ 478.2293, found 478.2291. Chiral HPLC 93% ee, Chiralpak IG column, heptane/isopropanol 99/01, flow 1.0 mL/min, tr(*S*) = 15.4 min, tr(*R*) = 32.8 min. $[\alpha]_D^{21}$ = -75 (c = 0.1, DCM). R_f, NMR (^{1}H and ^{13}C) and IR were identical to the enantiomer (*R*)-**4c**.

(*S*)-1-(2,8-*bis*(trifluoromethyl)quinolin-4-yl)-2-(4-heptylpiperazin-1-yl)ethan-1-ol 4d. To a solution of (*S*)-4-(oxiran-2-yl)-2,8-*bis*(trifluoromethyl)quinoline **13** (31 mg, 0.1 mmol, 1 eq.) in EtOH (1 mL) was added 1-heptylpiperazine **8** (42 mg, 0.2 mmol, 2 eq.) according to the procedure H. The product was concentrated *in vacuo* and purified by a chromatography on silica gel column (DCM/MeOH 100/0 to

70/30) to afford the product **4d** as a yellow oil. Yield: 63%. R_f: 0.6 (DCM/MeOH 9.8/0.2). ^{1}H NMR (400 MHz, $CDCl_3$) δ 8.19-8.14 (m, 3H), 7.71 (m, 1H), 5.54 (dd, ^3J = 10.5 Hz, ^3J = 3.4 Hz, 1H), 2.86-2.82 (m, 3.4 Hz, 3H), 2.62-2.46 (m, 7H), 2.39-2.35 (m, 2H), 1.52-1.46 (m, 2H), 1.31-1.25 (m, 8H), 0.90-0.87 (m, 3H) ppm. ^{13}C NMR (101 MHz, $CDCl_3$) δ 151.3, 148.8 (q, ^2J = 35.2 Hz), 143.8, 129.7 (q, ^2J = 30.0 Hz), 128.8 (q, ^3J = 5.4 Hz), 127.2, 126.9, 126.7, 123.6 (q, ^1J = 274.9 Hz), 121.4 (q, ^1J = 276.7 Hz), 114.6 (q, ^3J = 2.2 Hz), 65.2, 64.7, 58.8 (2C), 53.3 (2C), 31.9, 29.4, 27.0, 22.8, 14.2 ppm. IR ν_{max}: 2946, 2810, 1377, 1306, 1141, 1107, 769 cm^{-1}. HRMS (ESI) *m/z* calculated for $C_{24}H_{32}F_6N_3O$ [M+H]$^+$ 492.2450, found 492.2450. Chiral HPLC 85% ee, Chiralpak IG column, heptane/isopropanol 99/01, flow 1.0 mL/min, tr(*S*) = 14.4 min, tr(*R*) = 27.0 min. $[\alpha]_D^{21}$ = +59 (c = 0.1, DCM).

(*R*)-1-(2,8-*bis*(trifluoromethyl)quinolin-4-yl)-2-(4-heptylpiperazin-1-yl)ethan-1-ol 4d. To a solution of (*R*)-4-(oxiran-2-yl)-2,8-*bis*(trifluoromethyl)quinoline **13** (30 mg, 0.09 mmol, 1eq.) in EtOH (1 mL) was added 1-heptylpiperazine **8** (55 mg, 0.3 mmol, 3 eq.) according to the procedure H. The product was concentrated *in vacuo* and purified by a chromatography on silica gel column (DCM/MeOH 100/0 to 70/30) to afford the product **4d** as a yellow oil. Yield: 75%. HRMS (ESI) *m/z* calculated for $C_{24}H_{32}F_6N_3O$ [M+H]$^+$ 492.2450, found 492.2467. Chiral HPLC 85% ee, Chiralpak IG column, heptane/isopropanol 99/01, flow 1.0 mL/min, tr(*S*) = 14.2 min, tr(*R*) = 24.5 min. $[\alpha]_D^{21}$ = -66 (c = 0.1, DCM). R_f, NMR (^{1}H and ^{13}C) and IR were identical to the enantiomer (*S*)-**4d**.

(*S*)-1-(2,8-*bis*(trifluoromethyl)quinolin-4-yl)-2-(4-(4-methylphenethyl)piperazin-1-yl)ethan-1-ol 4e. To a solution of (*S*)-4-(oxiran-2-yl)-2,8-*bis*(trifluoromethyl)quinoline **13** (71 mg, 0.2 mmol, 1 eq.) in EtOH (1.7 mL) was added 1-(4-methylphenethyl)piperazine **9** (100 mg, 0.5 mmol, 3 eq.) according to the procedure H. The product was concentrated *in vacuo* and purified by a chromatography on silica gel column (DCM/MeOH 100/0 to 60/40) to afford the product **4e** as a pink solid. Yield: 44%. R_f: 0.36 (DCM/MeOH 9.9/0.1). m.p. 143-146 °C. ^{1}H NMR (400 MHz, $CDCl_3$) δ 8.18 (t, ^3J = 8.8 Hz, 3H), 7.73 (t, ^3J = 7.9 Hz, 1H), 7.11 (s, 4H), 5.57 (dd, ^3J = 11.0 Hz, ^3J = 2.9 Hz, 1H), 2.91 (s, 1H), 2.86 (dd, ^2J = 12.7 Hz, ^3J = 3.4 Hz, 1H), 2.88-2.78 (m, 2H), 2.67-2.62 (m, 8H), 2.51 (dd, ^2J = 12.7 Hz, ^3J = 10.5 Hz, 1H), 2.33 (s, 3H) ppm. ^{13}C NMR (101 MHz, $CDCl_3$) δ 151.2, 148.9 (q, ^2J = 35.2 Hz), 143.80, 137.0, 135.8, 129.8 (q, ^2J = 30.2 Hz), 129.3 (2C), 128.8 (q, ^3J = 5.5 Hz), 128.7 (2C), 127.2, 126.9, 126.7, 123.6 (q, ^1J = 271.9 Hz), 121.4 (q, ^1J = 276.9 Hz), 114.7 (q, ^3J = 2.0 Hz), 65.2, 64.7, 60.6, 53.3 (2C), 33.2, 21.2 ppm. IR ν_{max}: 2936, 1515, 1307, 1133 1107, 768 cm^{-1}. HRMS (ESI) *m/z* calculated for $C_{26}H_{28}F_6N_3O$ [M+H]$^+$ 512.2137, found 512.2153. Chiral HPLC 91% ee, Chiralpak IG column, heptane/isopropanol 95/05, flow 1.0 mL/min, tr(*S*) = 10.8 min, tr(*R*) = 14.0 min. $[\alpha]_D^{20}$ = +93 (c = 0.1, DCM).

(*R*)-1-(2,8-*bis*(trifluoromethyl)quinolin-4-yl)-2-(4-(4-methylphenethyl)piperazin-1-yl)ethan-1-ol 4e. To a solution of (*R*)-4-(oxiran-2-yl)-2,8-*bis*(trifluoromethyl)quinoline **13** (41 mg, 0.1 mmol, 1 eq.) in EtOH (1 mL) was added 1-(4-methylphenethyl)piperazine **9** (78 mg, 0.4 mmol, 3 eq.) according to the procedure H. The product was concentrated *in vacuo* and purified by a chromatography on silica gel column (DCM/MeOH 100/0 to 60/40) to afford the product **4e** as a pink solid. Yield: 77%. HRMS (ESI) *m/z* calculated for $C_{26}H_{28}F_6N_3O$ [M+H]$^+$ 512.2137, found 512.2156. Chiral HPLC 89% ee, Chiralpak IG column, heptane/isopropanol 95/05, flow 1.0 mL/min, tr(*S*) = 10.7 min, tr(*R*) = 12.9 min. $[\alpha]_D^{20}$ = -98 (c = 0.1, DCM). R_f, NMR (^{1}H and ^{13}C) and IR were identical to the enantiomer (*S*)-**4e**.

(*S*)-1-(2,8-*bis*(trifluoromethyl)quinolin-4-yl)-2-(4-(*p*-tolyl)piperazin-1-yl)ethan-1-ol 4f. To a solution of (*S*)-4-(oxiran-2-yl)-2,8-*bis*(trifluoromethyl)quinoline **13** (32 mg, 0.1 mmol, 1 eq.) in EtOH (1 mL) was added commercial 1-(*p*-tolyl)piperazine (53 mg, 0.3 mmol, 3 eq.) according to the procedure H. The product was concentrated *in vacuo* and purified by a chromatography on silica gel column

(DCM/MeOH 100/0 to 85/15) to afford the product **4f** as an orange oil. Yield: 79%. R_f: 0.17 (cyclohexane/EtOAc 4/1). ^{1}H NMR (400 MHz, $CDCl_3$) δ 8.22-8.16 (m, 3H), 7.76-7.72 (m, 1H), 7.11 (d, ^3J = 9.5 Hz, 2H), 6.88 (d, ^3J = 8.6 Hz, 2H), 5.61 (dd, ^3J = 10.5 Hz, ^3J = 2.5 Hz, 1H), 3.30-3.20 (m, 4H), 3.04-3.0 (m, 2H), 2.91 (dd, ^2J = 12.7 Hz, ^3J = 3.3 Hz, 1H), 2.74-2.69 (m, 2H), 2.57 (dd, ^2J = 12.7 Hz, ^3J = 10.5 Hz, 1H), 2.30 (s, 3H) ppm. ^{13}C NMR (101 MHz, $CDCl_3$) δ 151.1, 149.0 (q, ^2J = 35.1 Hz), 149.0, 143.8, 130.1, 130.0 (2C), 129.9, 128.9 (q, ^3J = 5.0 Hz), 127.3, 126.9, 126.7, 125.2 (q, ^1J = 275.2 Hz), 122.5 (q, ^1J = 276.0 Hz), 116.9 (2C), 114.8 (q, ^3J = 1.7 Hz), 65.3, 64.8, 53.4 (2C), 50.0 (2C), 20.7 ppm. IR ν_{max}: 2954, 1510, 1307, 1136, 1107, 819 cm^{-1}. HRMS (ESI) *m/z* calculated for $C_{24}H_{24}F_6N_3O$ $[M+H]^+$ 484.1824, found 484.1828. Chiral HPLC 92% ee, Chiralpak IB column, heptane/isopropanol 99/01, flow 1.0 mL/min, tr(*S*) = 33.0 min, tr(*R*) = 39.9 min. $[\alpha]_D^{21}$ = +53 (c = 0.1, DCM).

(*R*)-1-(2,8-*bis*(trifluoromethyl)quinolin-4-yl)-2-(4-(*p*-tolyl)piperazin-1-yl)ethan-1-ol 4f. To a solution of (*R*)-4-(oxiran-2-yl)-2,8-*bis*(trifluoromethyl)quinoline **13** (32 mg, 0.1 mmol, 1 eq.) in EtOH (1 mL) was added commercial 1-(*p*-tolyl)piperazine (53 mg, 0.3 mmol, 3 eq.) according to the procedure H. The product was concentrated *in vacuo* and purified by a chromatography on silica gel column (DCM/MeOH 100/0 to 85/15) to afford the product **4f** as an orange oil. Yield: 69%. HRMS (ESI) *m/z* calculated for $C_{24}H_{24}F_6N_3O$ $[M+H]^+$ 484.1824, found 484.1831. Chiral HPLC 94% ee, Chiralpak IB column, heptane/isopropanol 99/01, flow 1.0 mL/min, tr(*S*) = 34.3 min, tr(*R*) = 38.0 min. $[\alpha]_D^{21}$ = -51 (c = 0.1, DCM). R_f, NMR (^{1}H and ^{13}C) and IR were identical to the enantiomer (*S*)-**4f**.

S2. Mosher's esters synthesis and analysis.

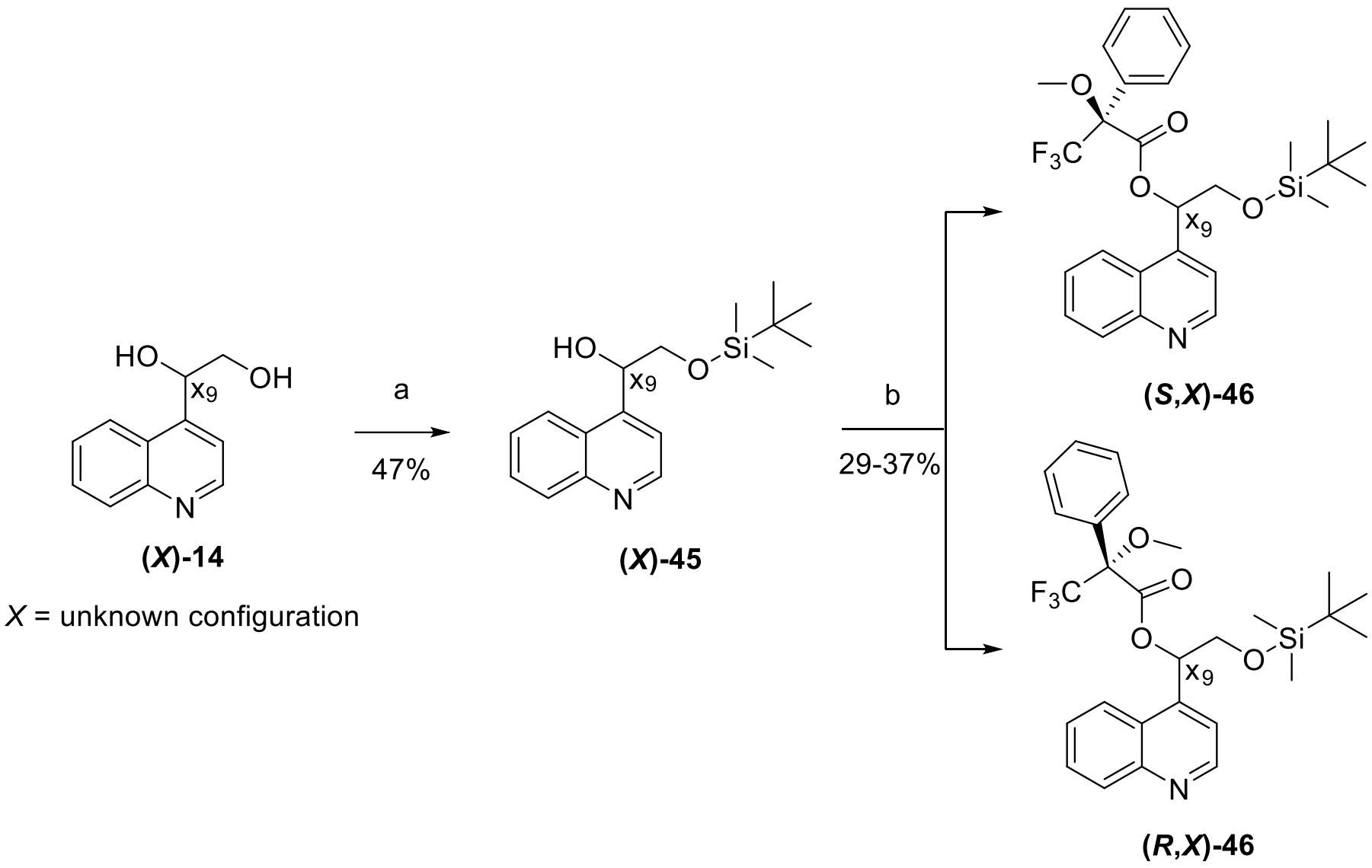

Scheme 2. Synthetic route for Mosher's esters **46**. Reagents and conditions: (a) TBDMSCl, Imidazole, NEt_3, anhydrous DMF, 16 °C, 78 h. (b) (*R*/*S*)-MTPA, EDCI, DMAP, CH_2Cl_2, 0-18 °C, 48 h.

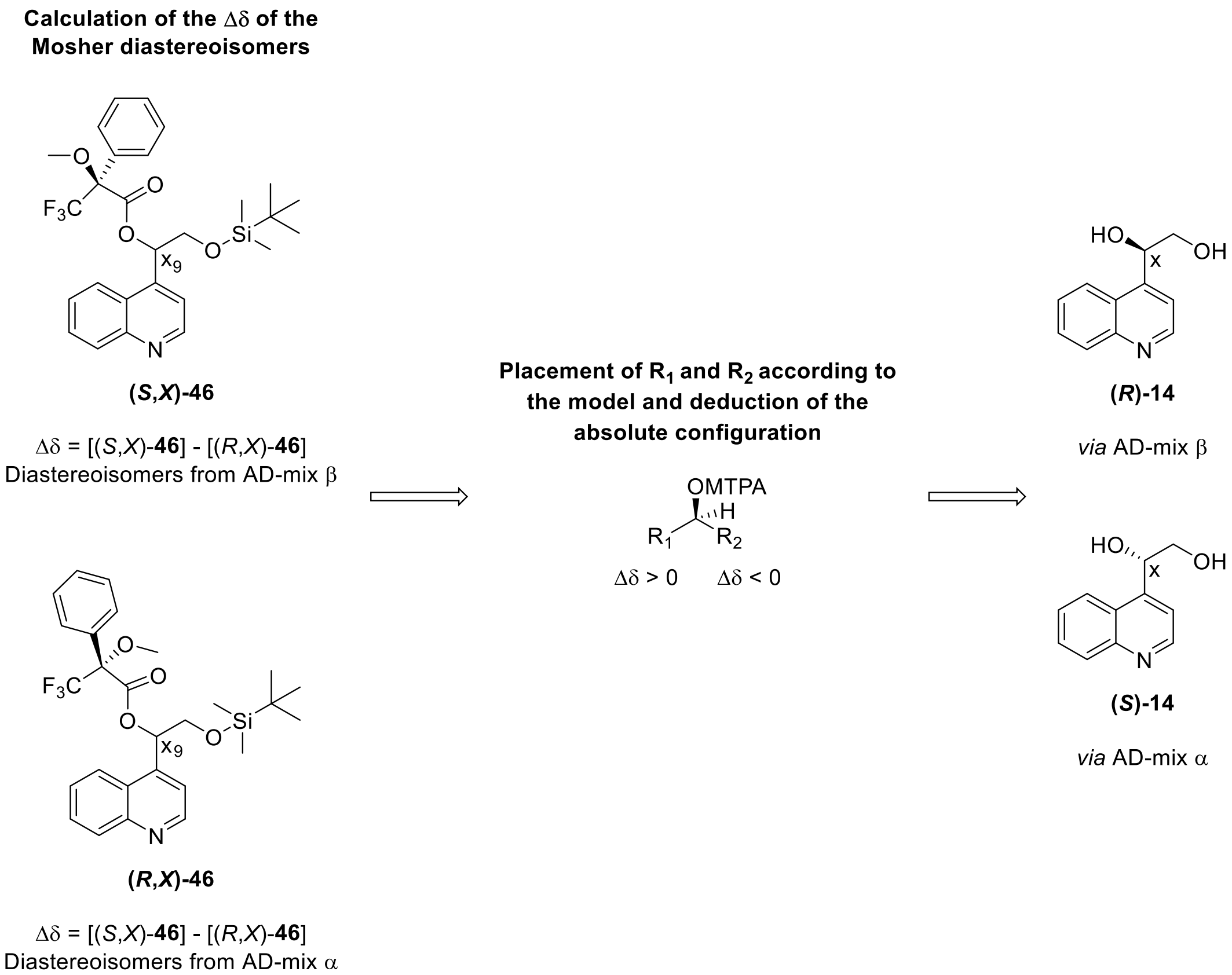

Figure S1. Mosher's interpretation.

S3. ^{1}H and ^{13}C-NMR spectra of compounds 1-4a-f.

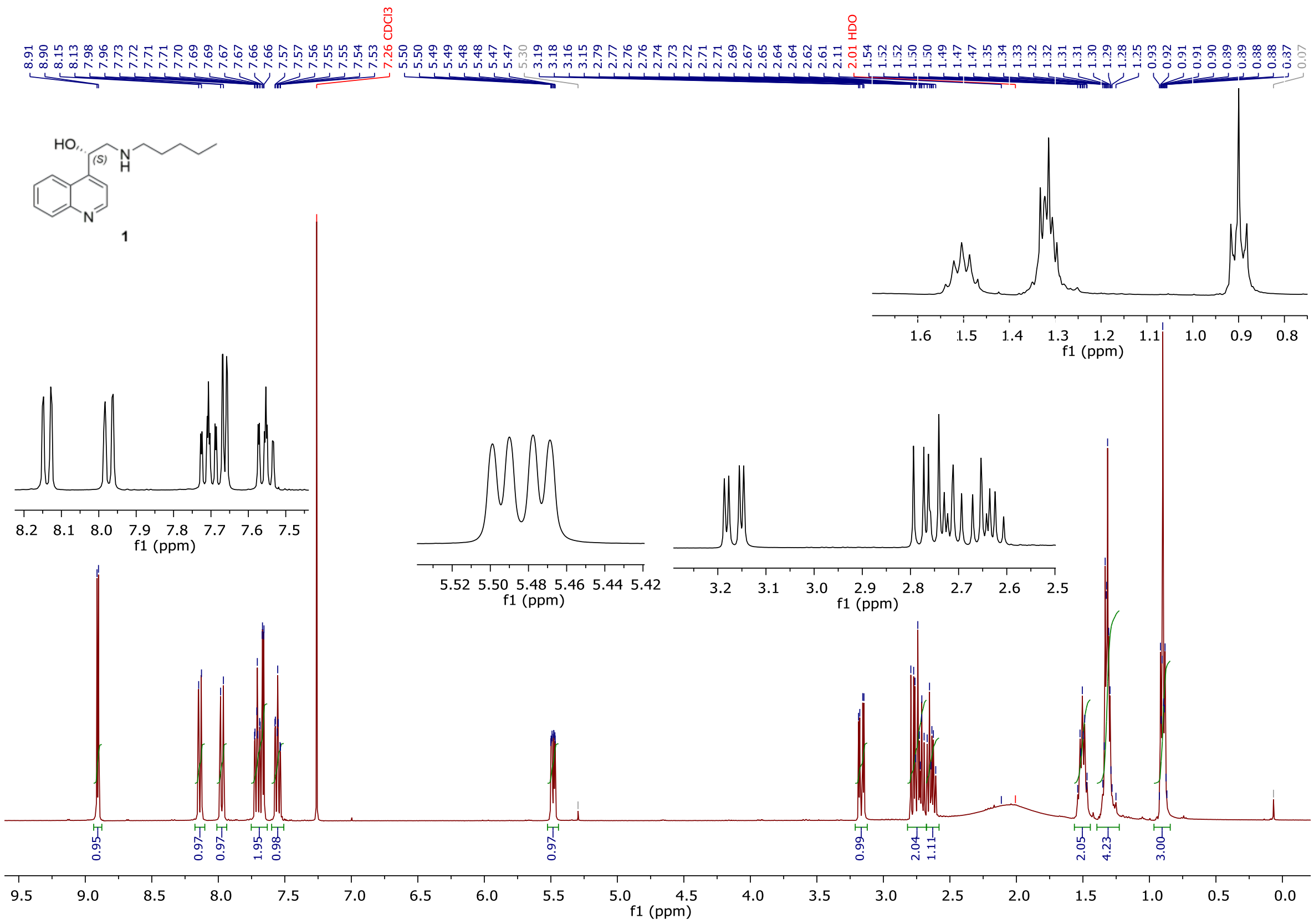

Figure S2. ^{1}H NMR Spectrum (400 MHz, $CDCl_3$) of compound (*S*)-**1**.

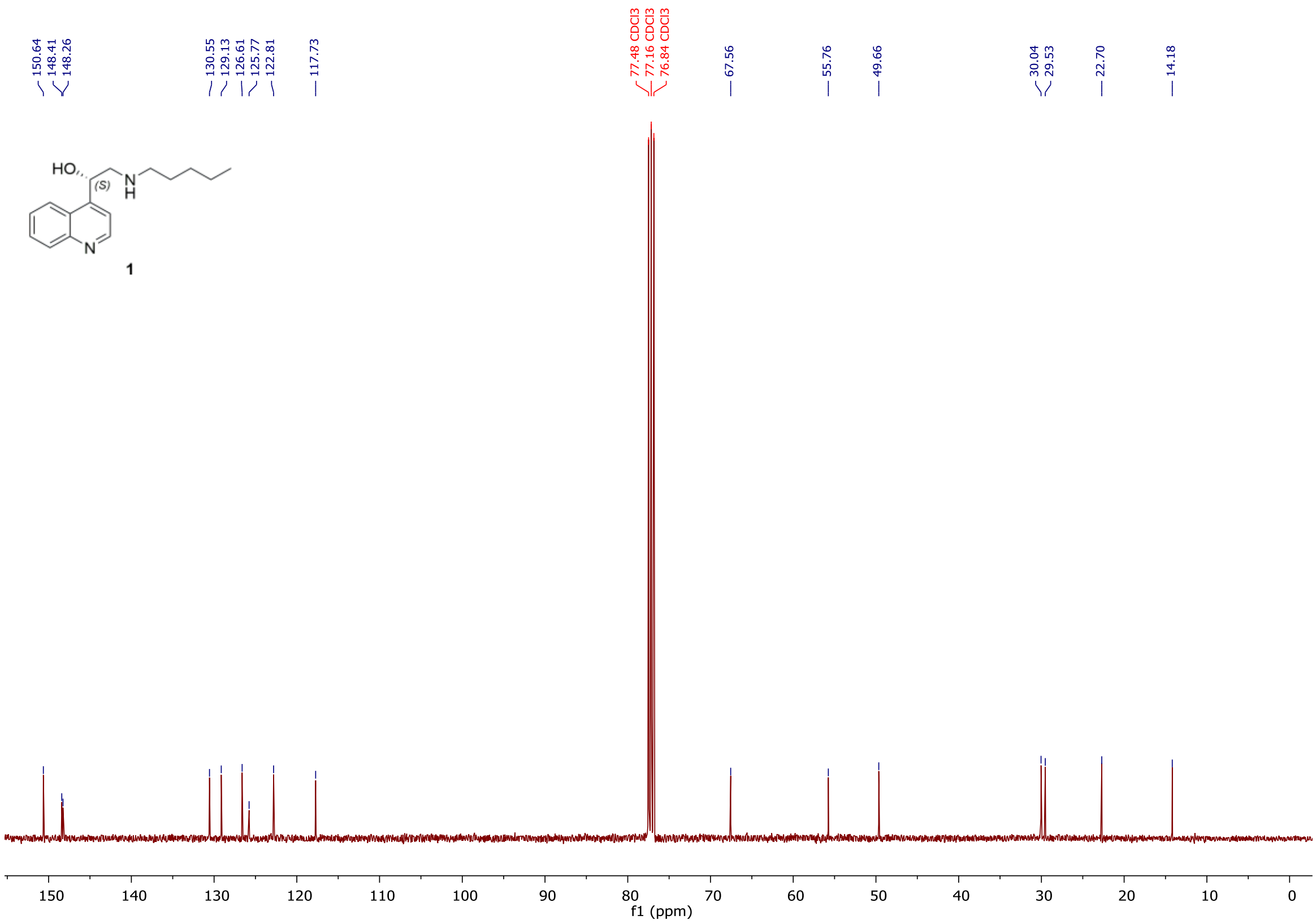

Figure S3. ^{13}C NMR Spectrum (101 MHz, $CDCl_3$) of compound (*S*)-**1**.

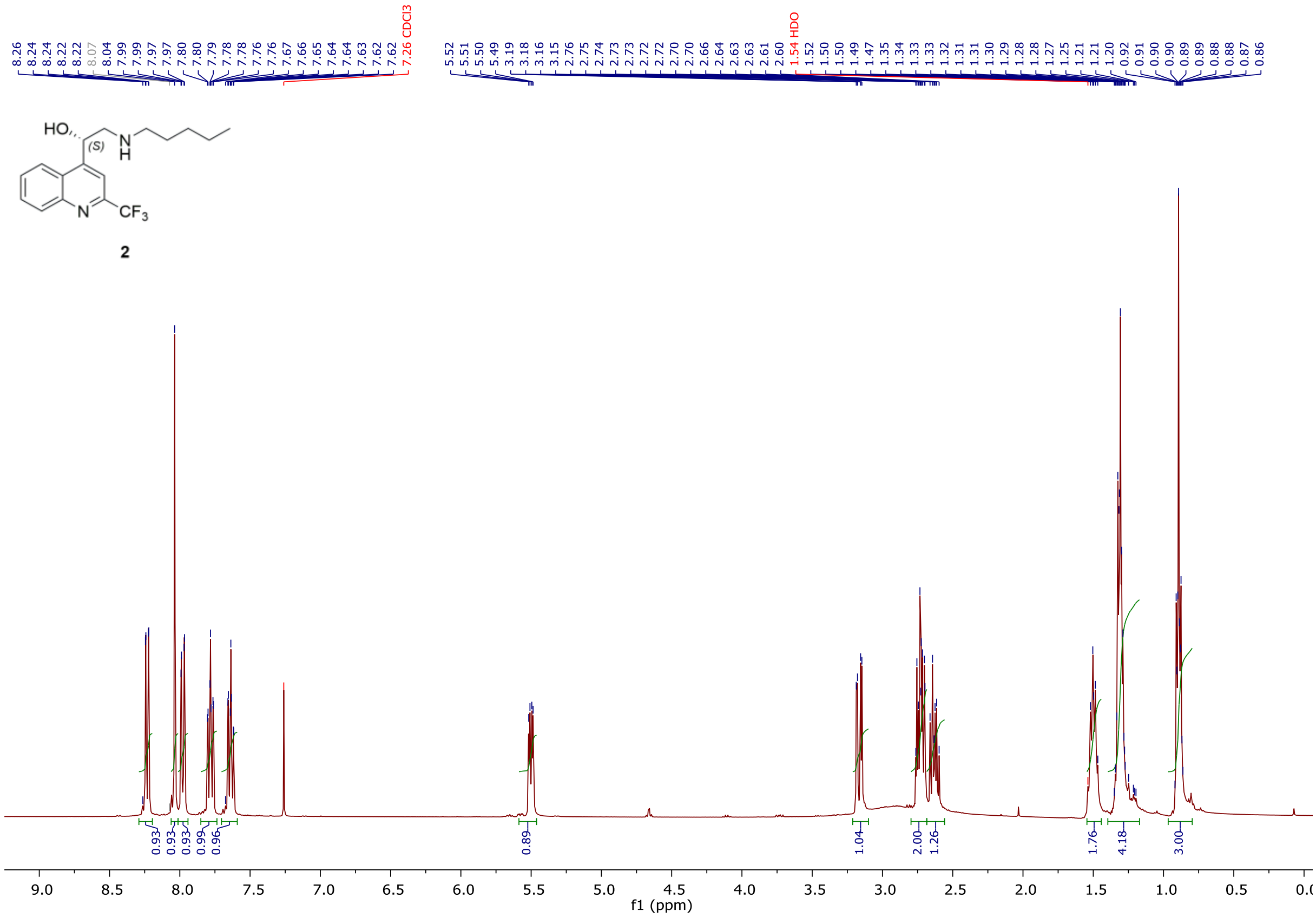

Figure S4. ^{1}H NMR Spectrum (400 MHz, $CDCl_3$) of compound (*S*)-**2**.

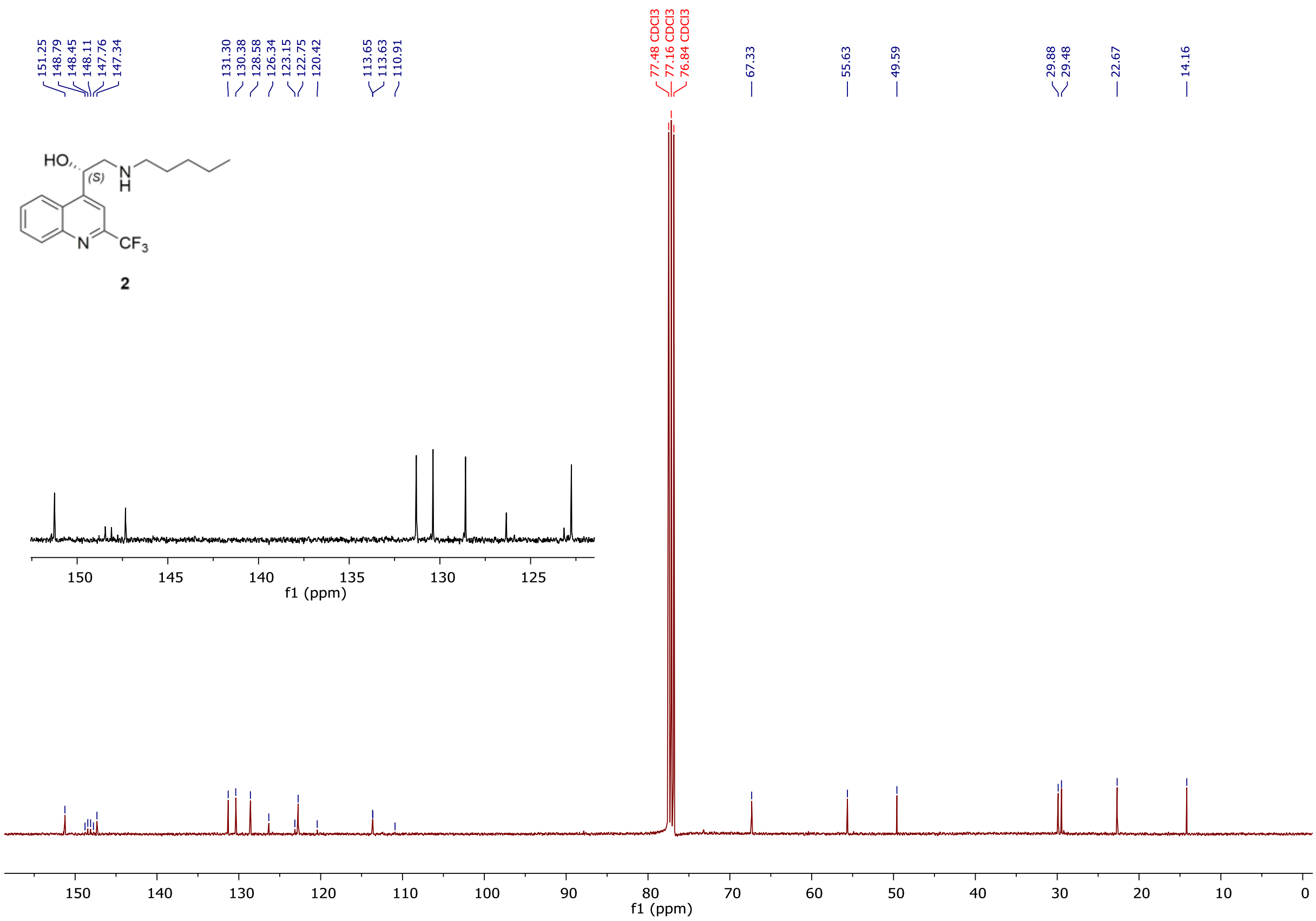

Figure S5. ^{13}C NMR Spectrum (101 MHz, $CDCl_3$) of compound (*S*)-**2**.

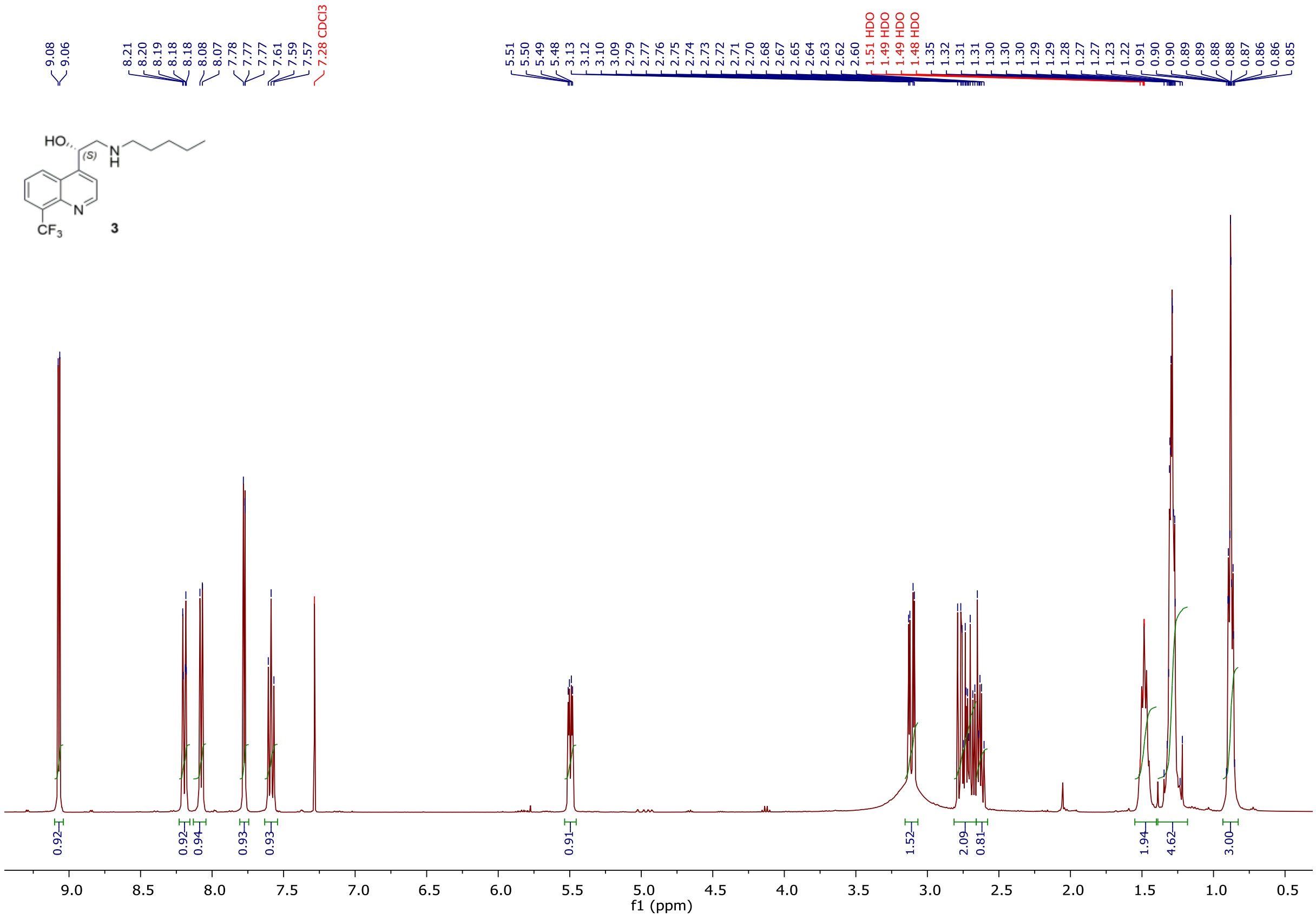

Figure S6. ^{1}H NMR Spectrum (400 MHz, $CDCl_3$) of compound **3a**.

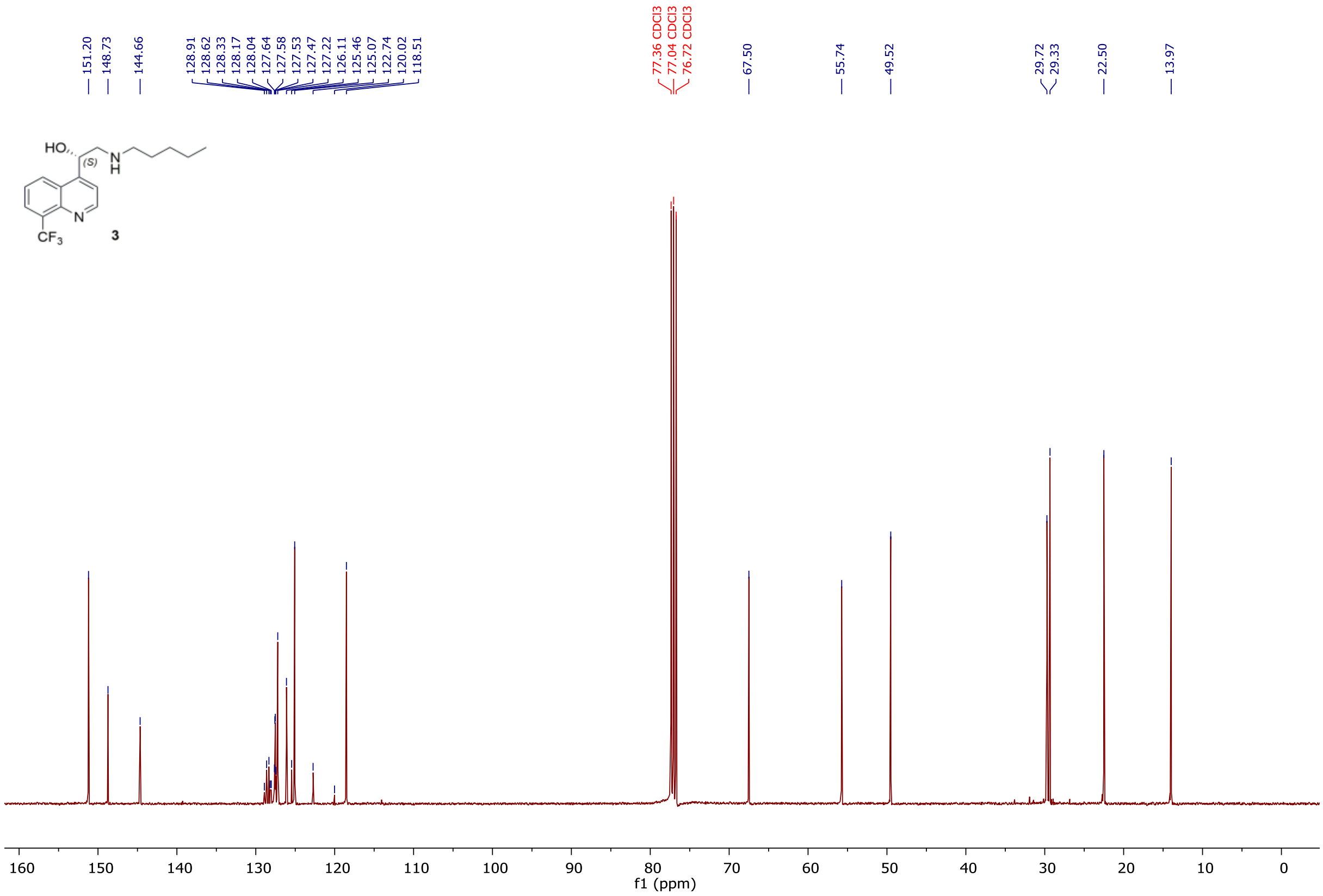

Figure S7. ^{13}C NMR Spectrum (101 MHz, $CDCl_3$) of compound **3b**.

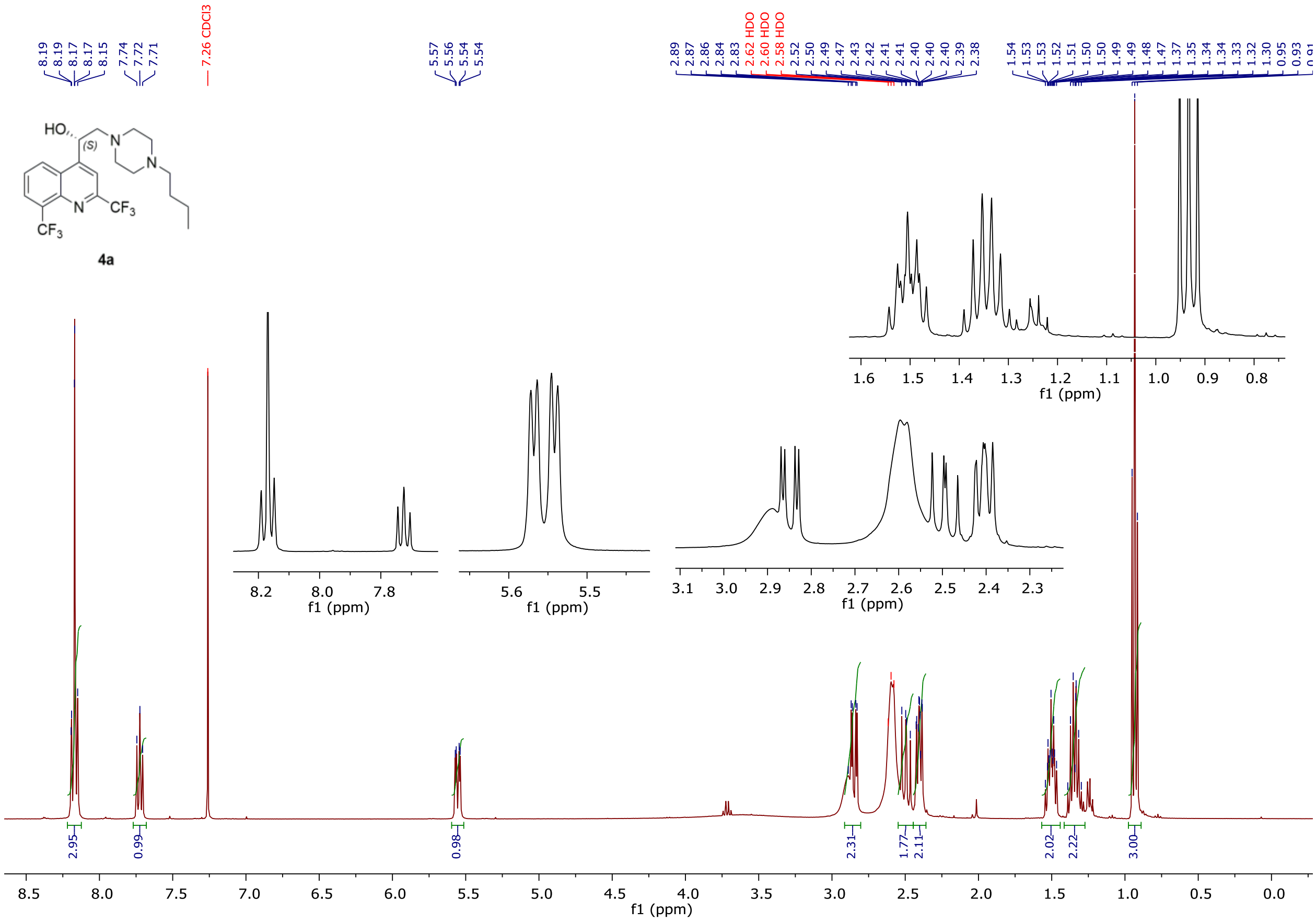

Figure S8. ^{1}H NMR Spectrum (400 MHz, $CDCl_3$) of compound (*S*)-**4a**.

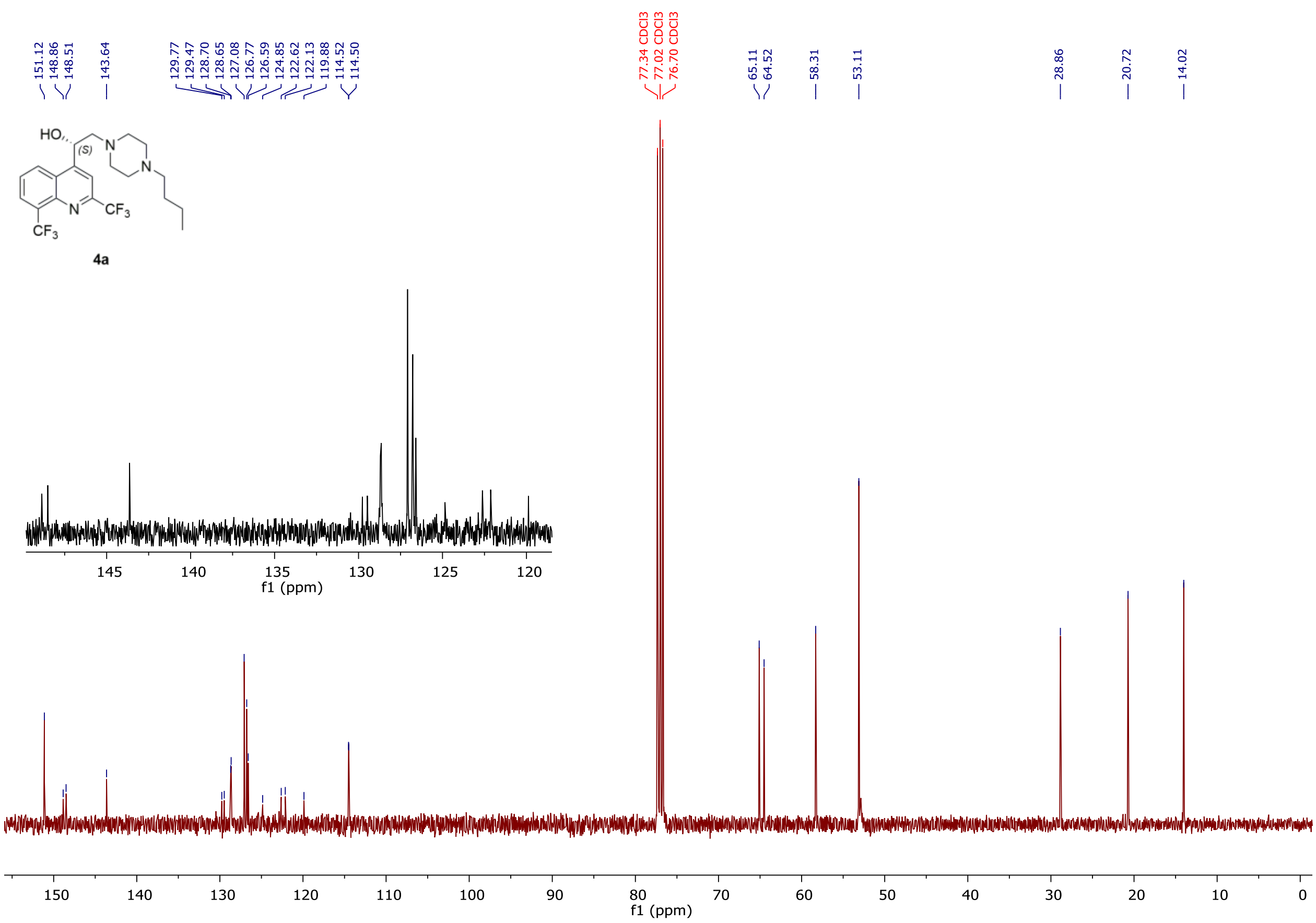

Figure S9. ^{13}C NMR Spectrum (101 MHz, $CDCl_3$) of compound (*S*)-**4a**.

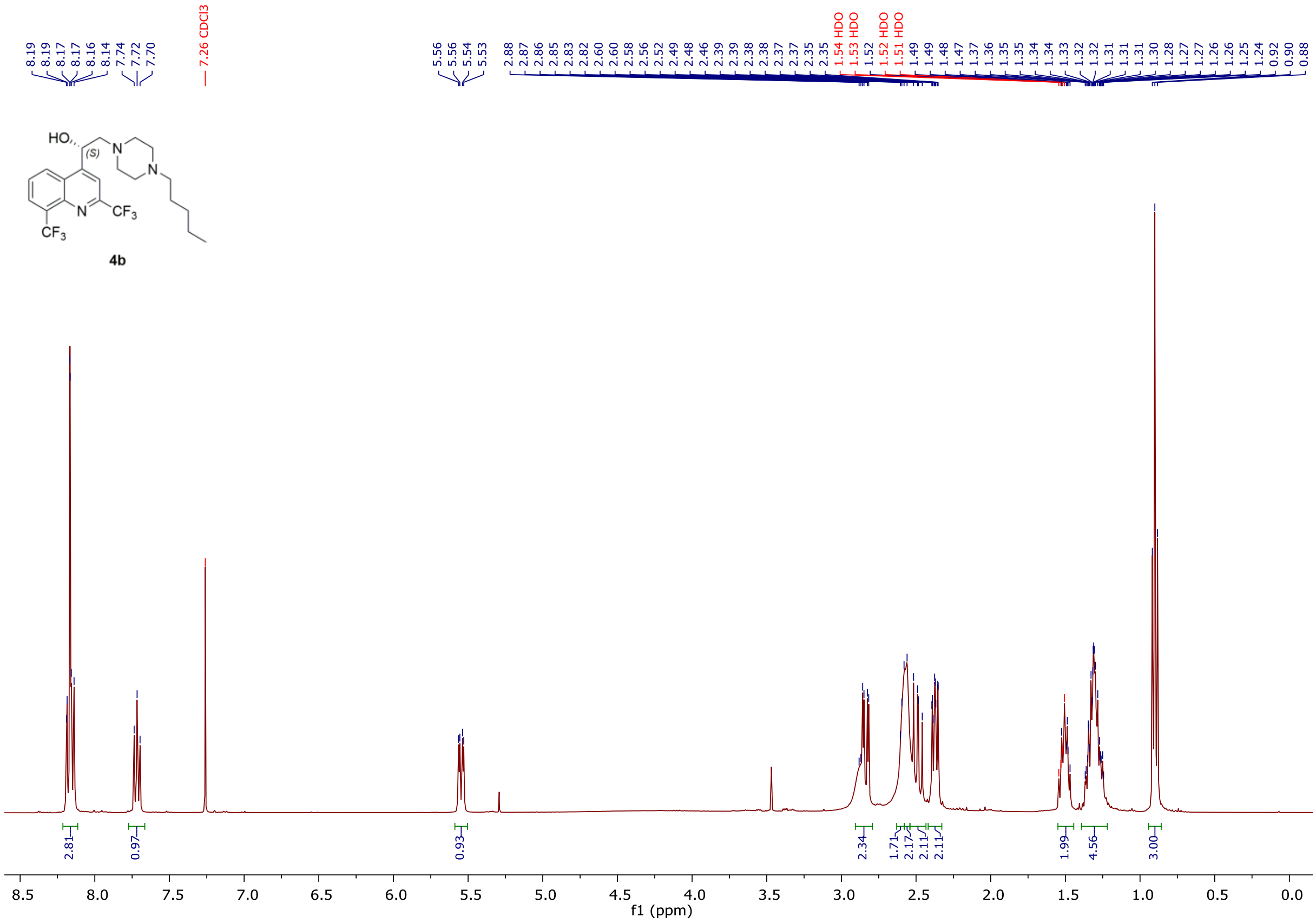

Figure S10. ^{1}H NMR Spectrum (400 MHz, $CDCl_3$) of compound (*S*)-**4b**.

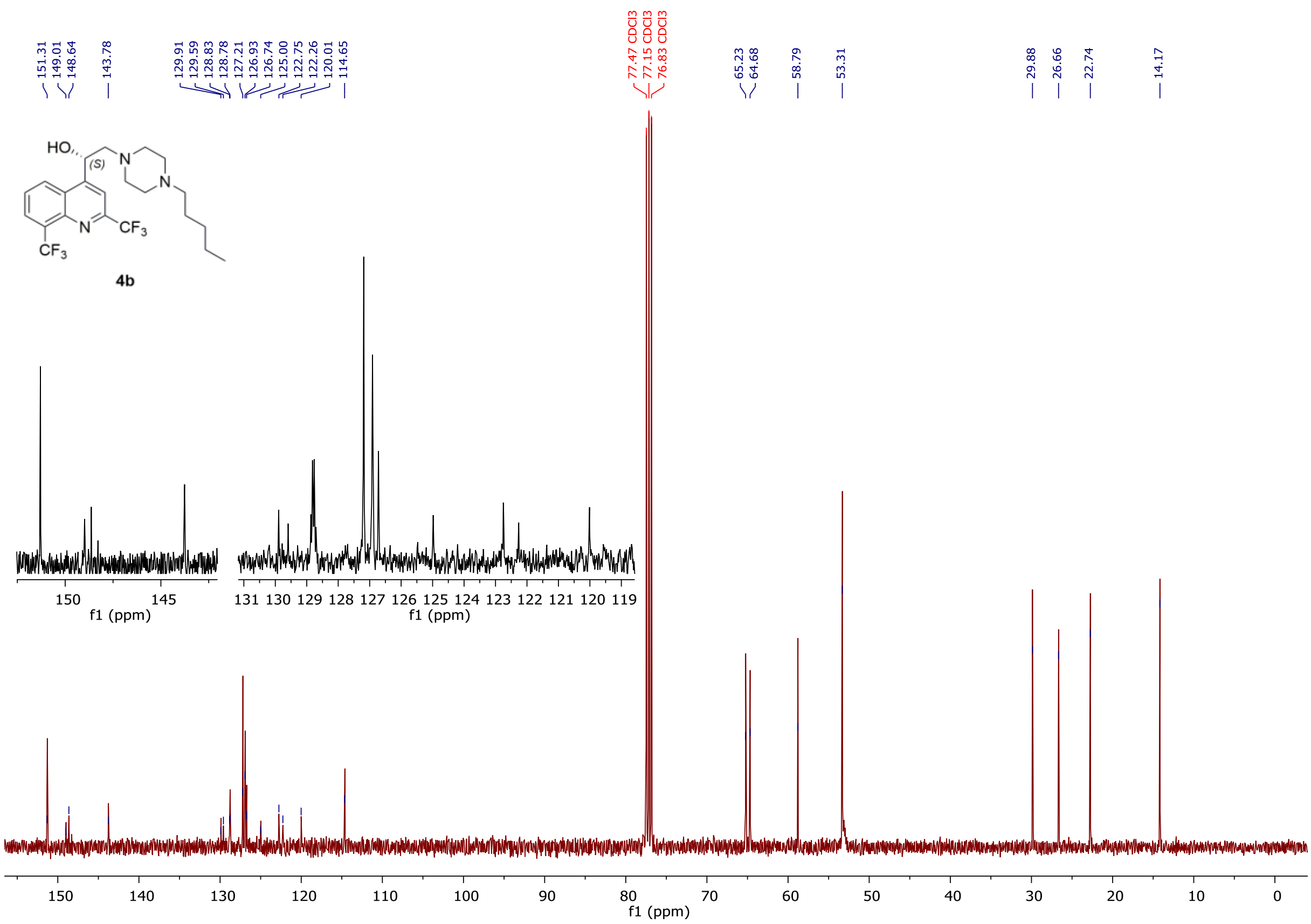

Figure S11. ^{13}C NMR Spectrum (101 MHz, $CDCl_3$) of compound (*S*)-**4b**.

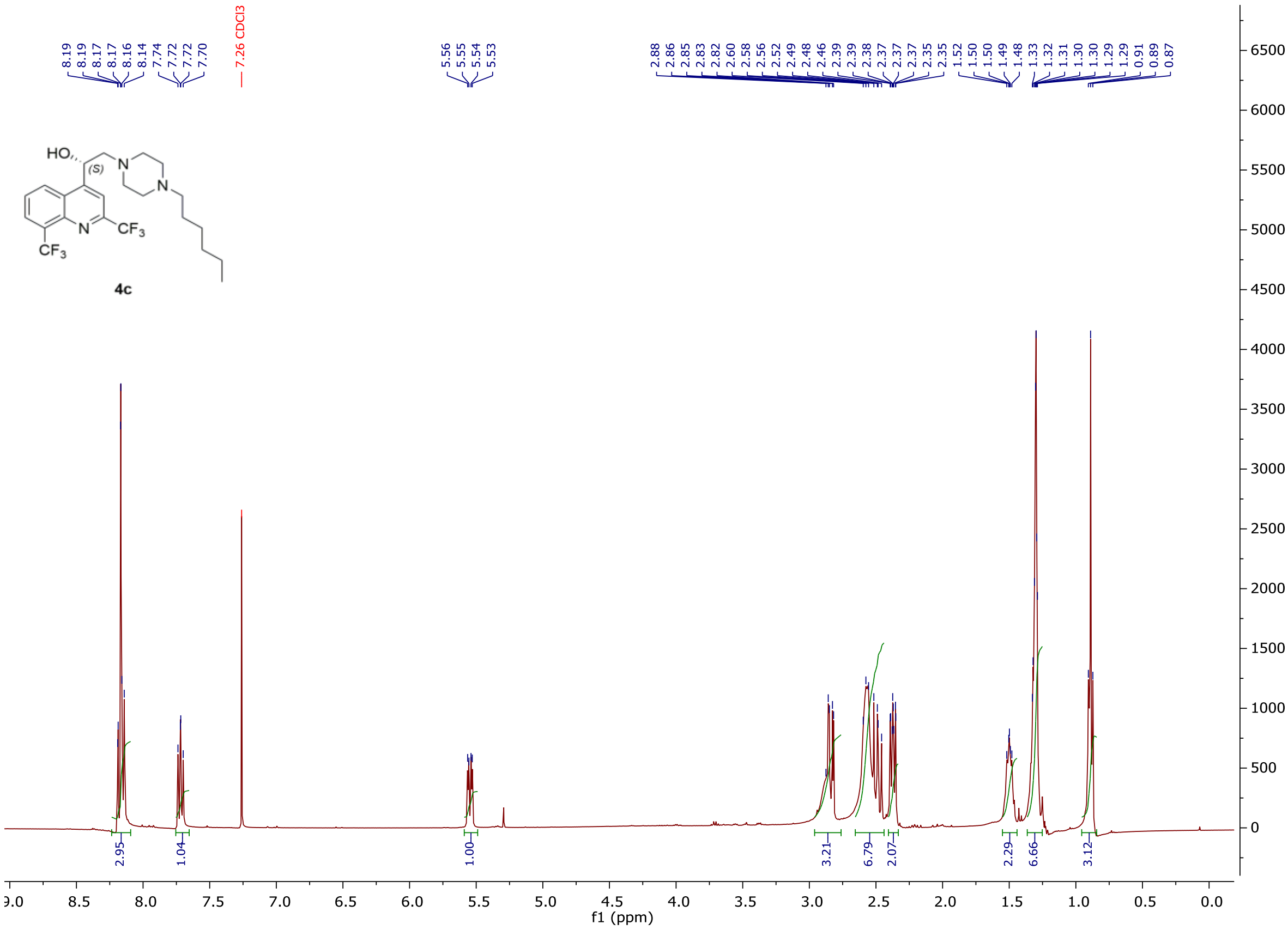

Figure S12. ^{1}H NMR Spectrum (400 MHz, $CDCl_3$) of compound (*S*)-**4c**.

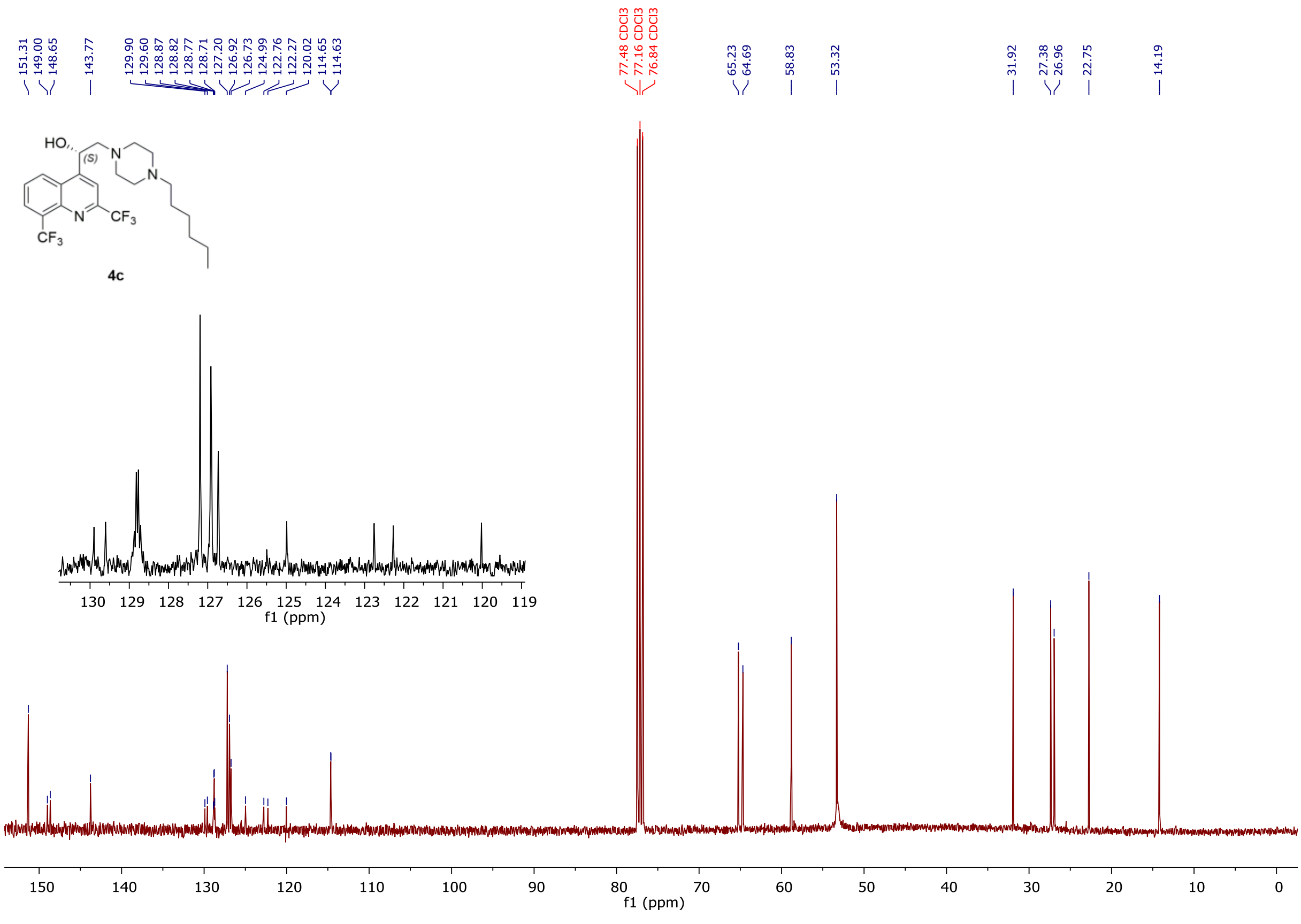

Figure S13. ^{13}C NMR Spectrum (101 MHz, $CDCl_3$) of compound (*S*)-**4c**.

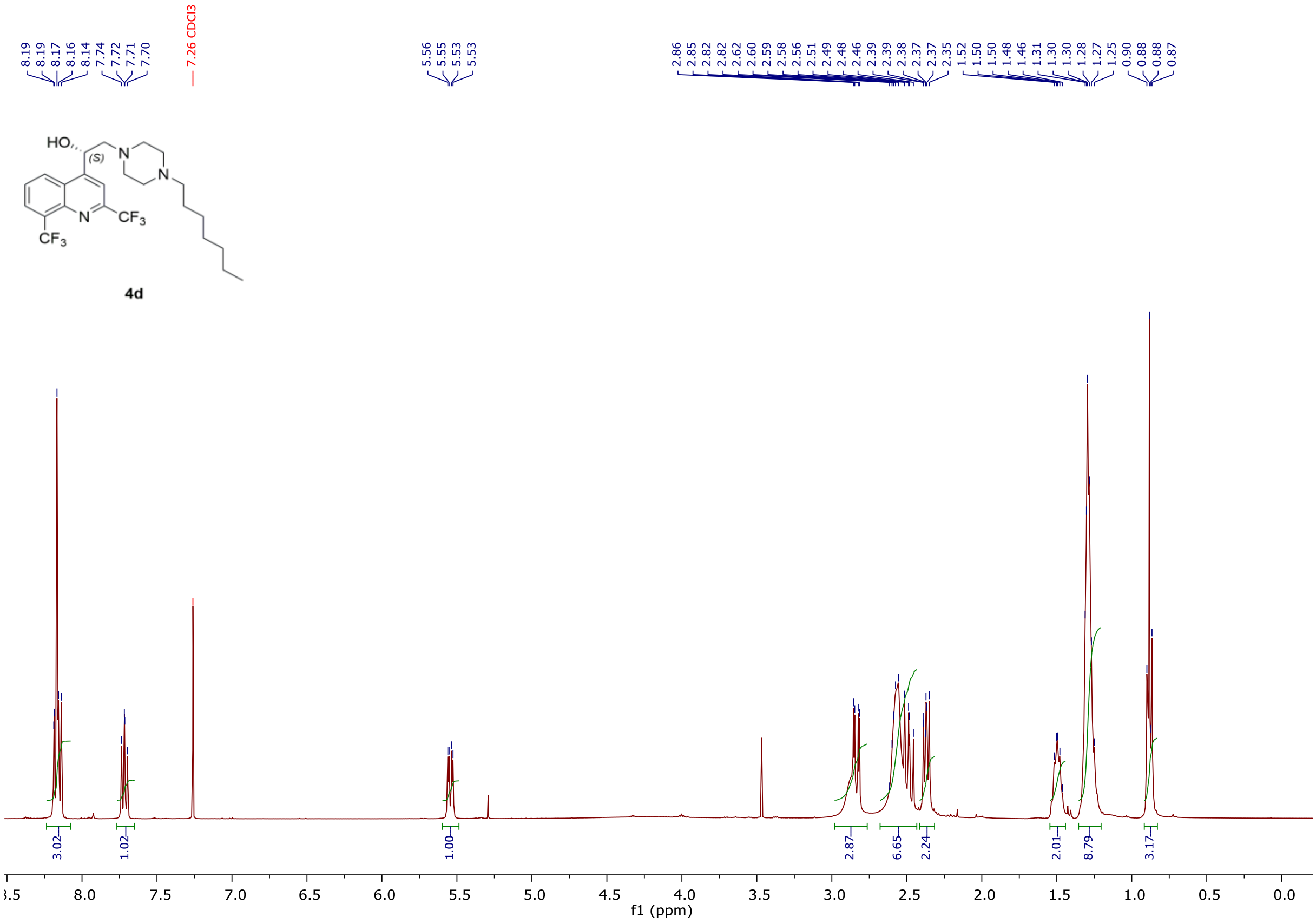

Figure S14. ^{1}H NMR Spectrum (400 MHz, $CDCl_3$) of compound (*S*)-**4d**.

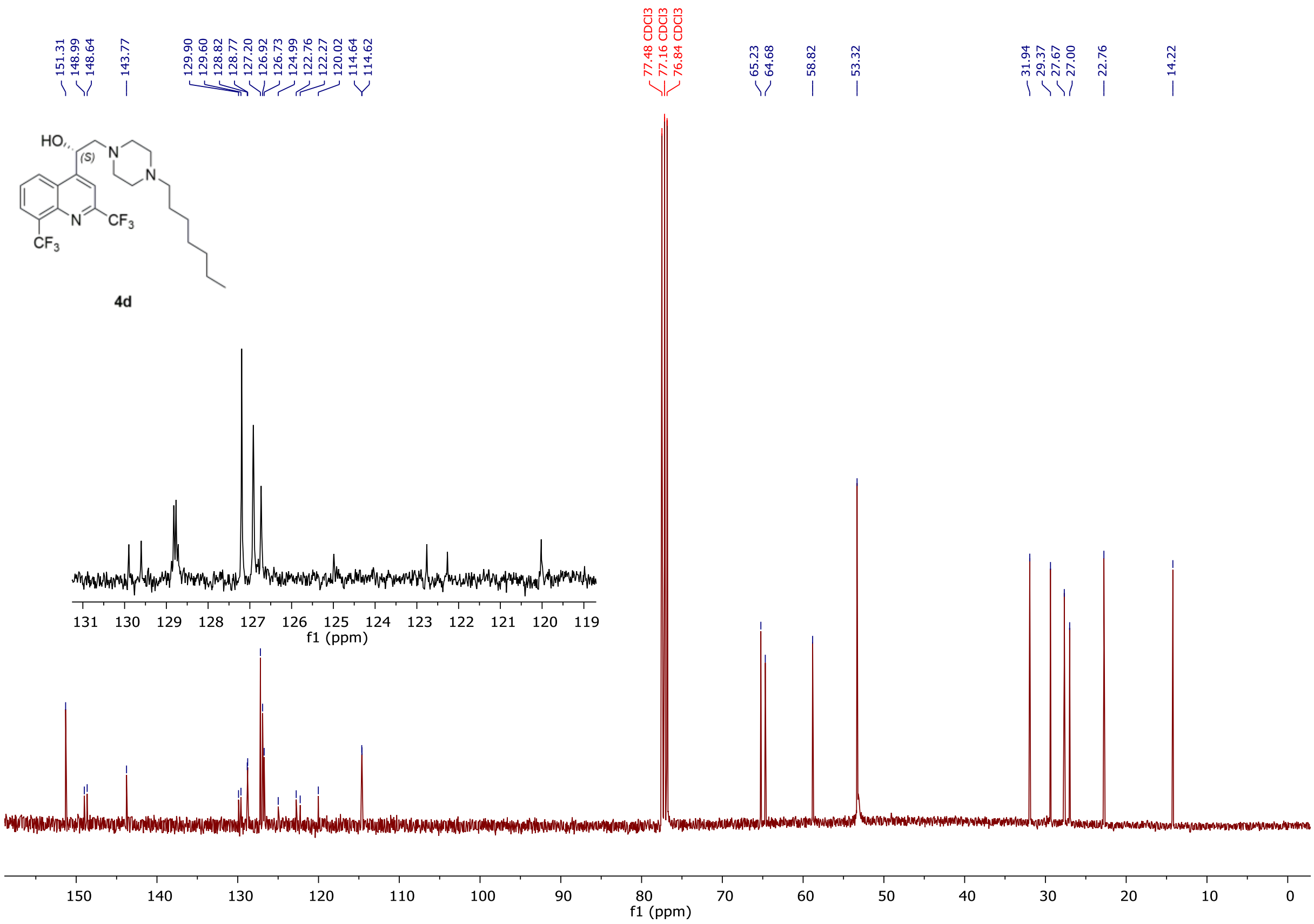

Figure S15. ^{13}C NMR Spectrum (101 MHz, $CDCl_3$) of compound (*S*)-**4d**.

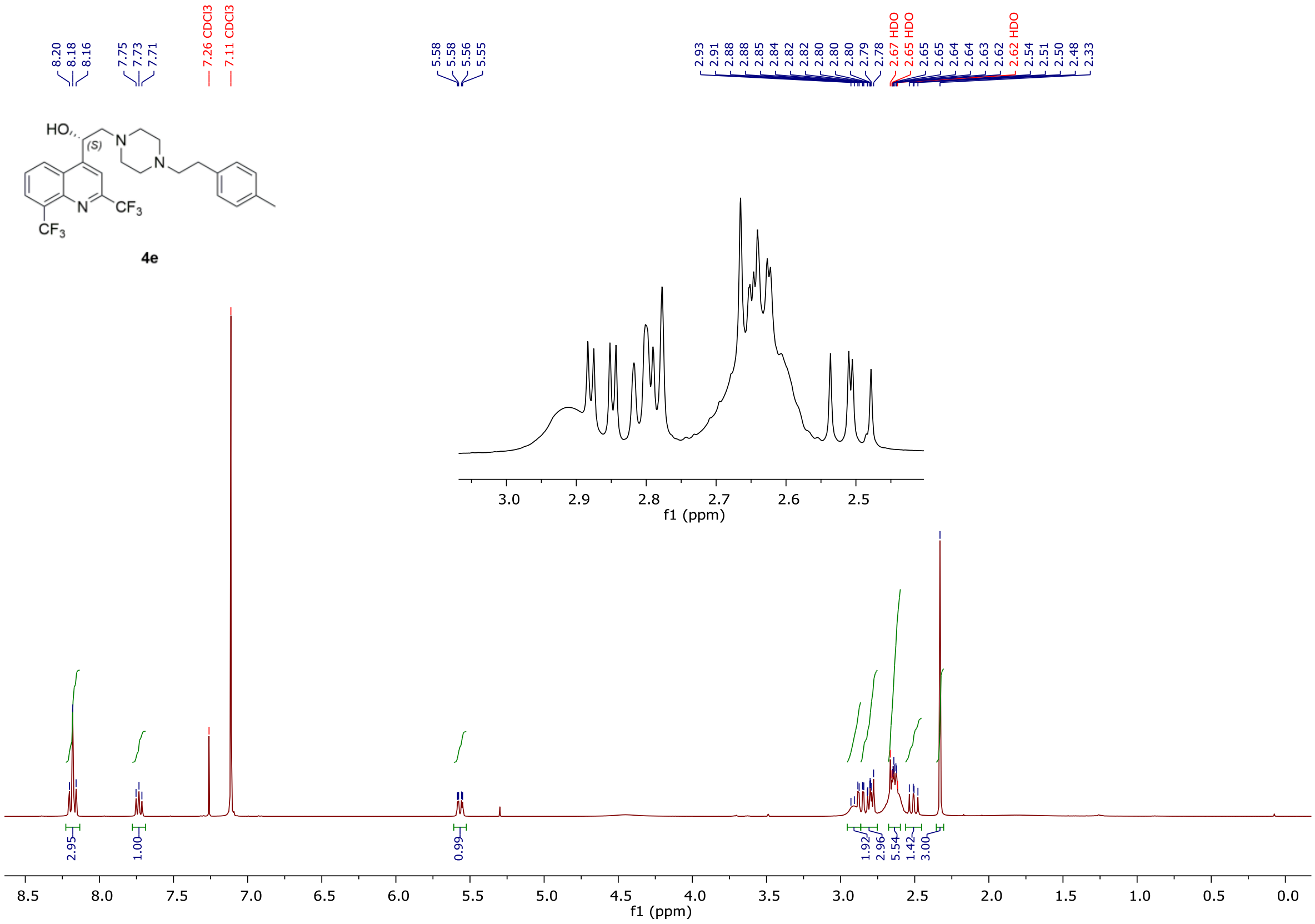

Figure S16. ^{1}H NMR Spectrum (400 MHz, $CDCl_3$) of compound (*S*)-**4e**.

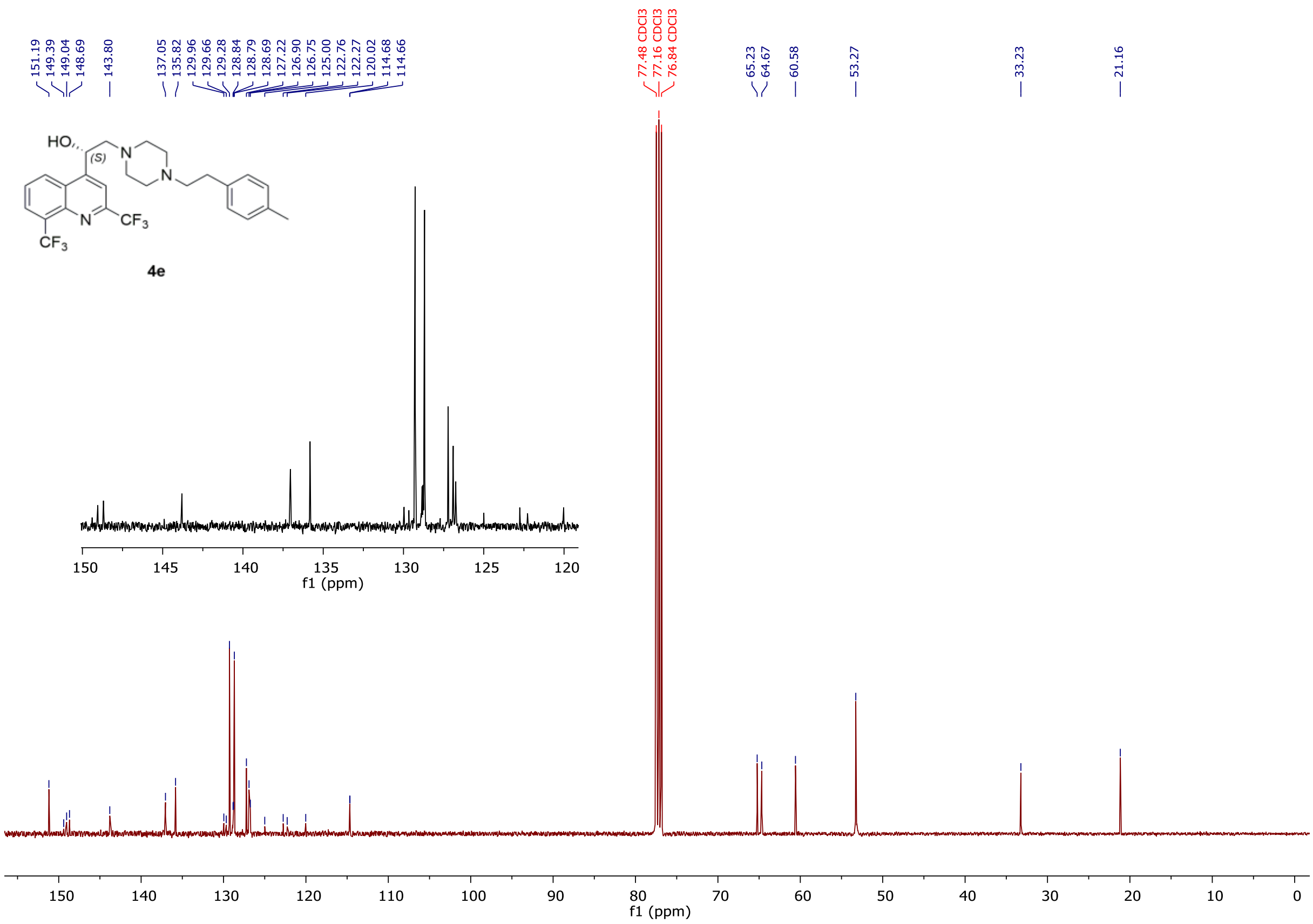

Figure S17. ^{13}C NMR Spectrum (101 MHz, $CDCl_3$) of compound (*S*)-**4e**.

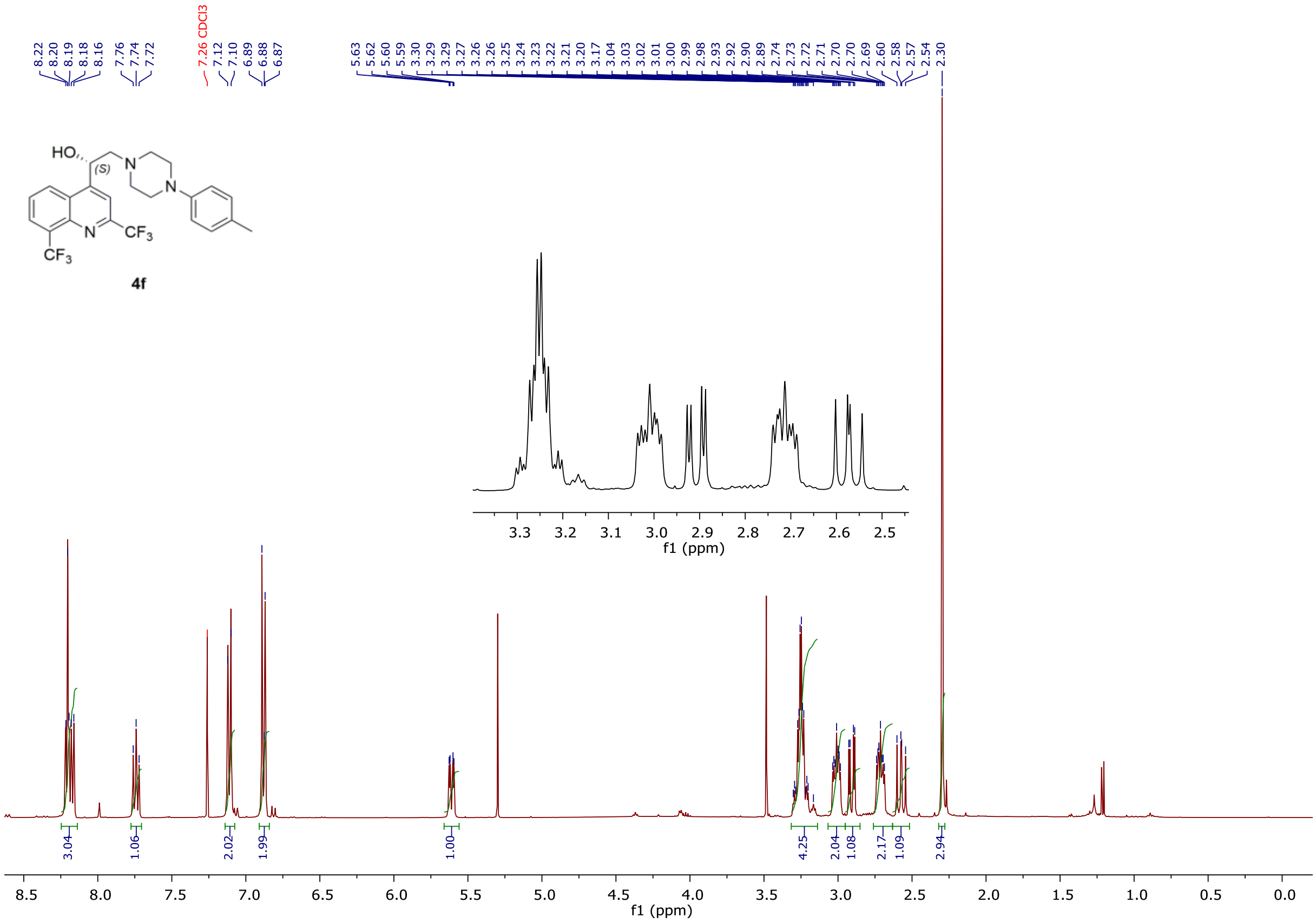

Figure S18. ^{1}H NMR Spectrum (400 MHz, $CDCl_3$) of compound (*S*)-**4f**.

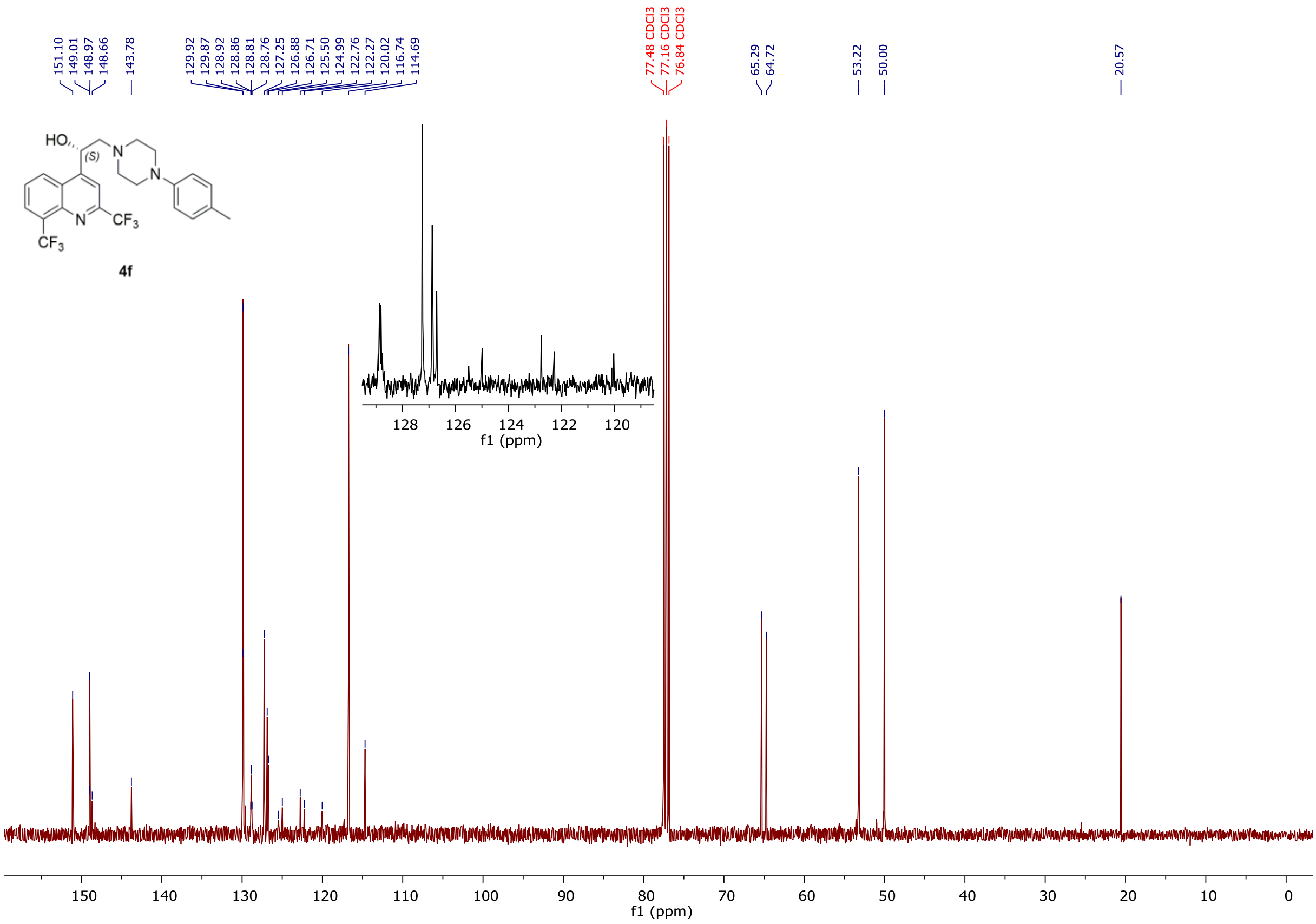

Figure S19. ^{13}C NMR Spectrum (101 MHz, $CDCl_3$) of compound (*S*)-**4f**.

S4. HRMS spectra of compounds 1-4a-f.

Monoisotopic Mass, Even Electron Ions
84 formula(e) evaluated with 1 results within limits (up to 50 closest results for each mass)
Elements Used:
C: 0-25 H: 0-25 N: 0-5 O: 0-5
SD-EC1-133 41 (0.180) Cm (41:53)
1: TOF MS ES+

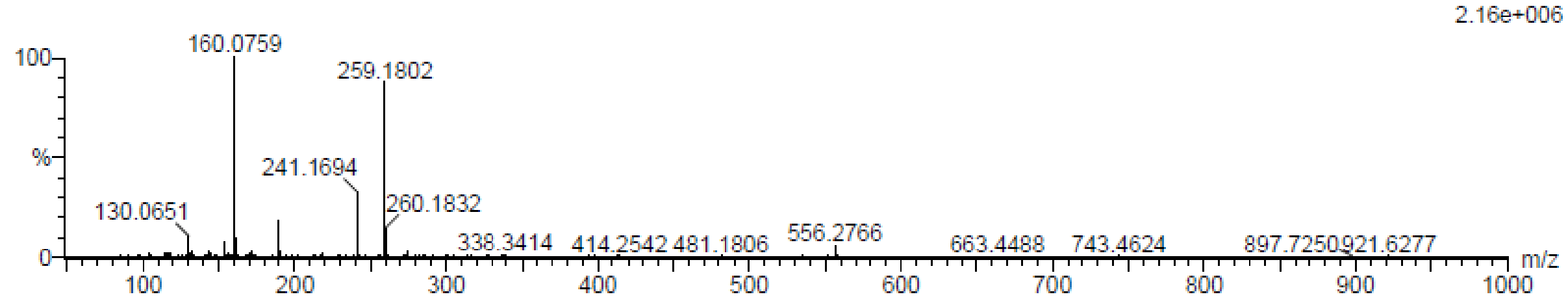

Minimum:				-1.5				
Maximum:		2.0	5.0	50.0				
Mass	Calc. Mass	mDa	PPM	DBE	i-FIT	Norm	Conf(%)	Formula
259.1802	259.1810	-0.8	-3.1	6.5	1469.0	n/a	n/a	C16 H23 N2 O

Figure S20. HRMS spectrum of (*S*)-**1**.

Monoisotopic Mass, Even Electron Ions
34 formula(e) evaluated with 1 results within limits (up to 50 closest results for each mass)
Elements Used:
C: 0-19 H: 0-30 N: 0-2 O: 0-2 F: 0-3
SD-EC3-405 121 (0.267) Cm (120:146)

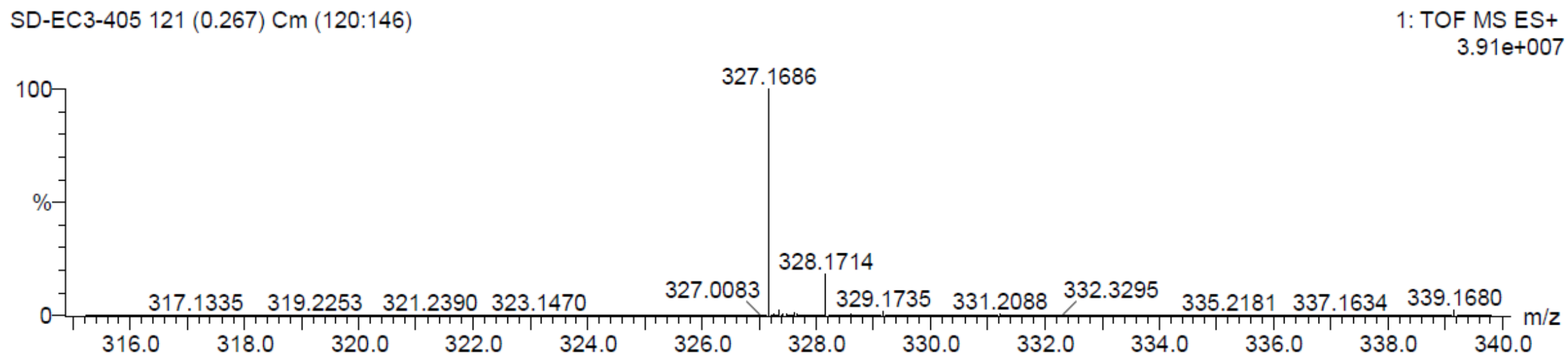

Minimum:				-5.0				
Maximum:		2.0	5.0	150.0				
Mass	Calc. Mass	mDa	PPM	DBE	i-FIT	Norm	Conf(%)	Formula
327.1686	327.1684	0.2	0.6	6.5	3180.0	n/a	n/a	C17 H22 N2 O F3

Figure S21. HRMS spectrum of (*S*)-**2**.

Monoisotopic Mass, Even Electron Ions
34 formula(e) evaluated with 1 results within limits (up to 50 closest results for each mass)
Elements Used:
C: 0-19 H: 0-30 N: 0-2 O: 0-2 F: 0-3
SD-EC3-403 138 (0.299) Cm (138:195)

1: TOF MS ES+
9.55e+006

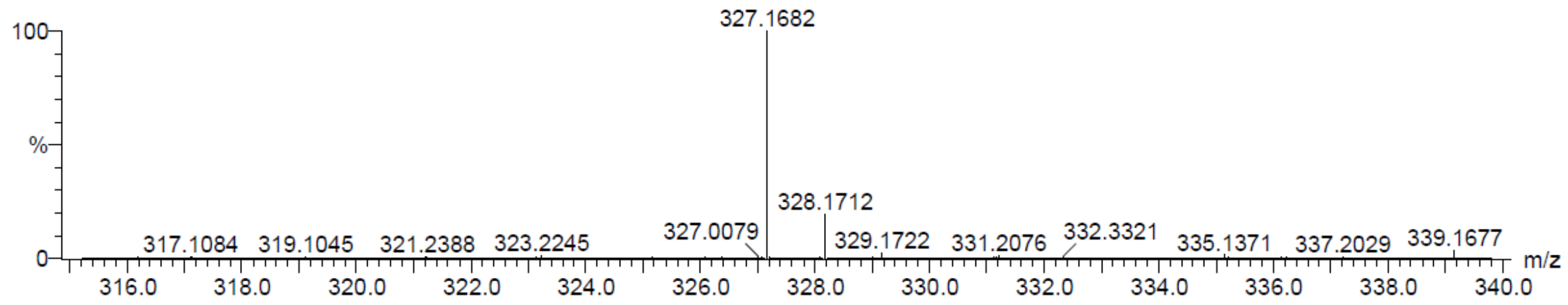

Minimum:				-5.0				
Maximum:		2.0	5.0	150.0				
Mass	Calc. Mass	mDa	PPM	DBE	i-FIT	Norm	Conf(%)	Formula
327.1682	327.1684	-0.2	-0.6	6.5	3851.4	n/a	n/a	C17 H22 N2 O F3

Figure S22. HRMS spectrum of (*R*)-**2**.

Monoisotopic Mass, Even Electron Ions
34 formula(e) evaluated with 1 results within limits (up to 50 closest results for each mass)
Elements Used:
C: 0-19 H: 0-30 N: 0-2 O: 0-2 F: 0-3
SD-EC3-435 174 (0.379) Cm (172:255)

1: TOF MS ES+
2.56e+007

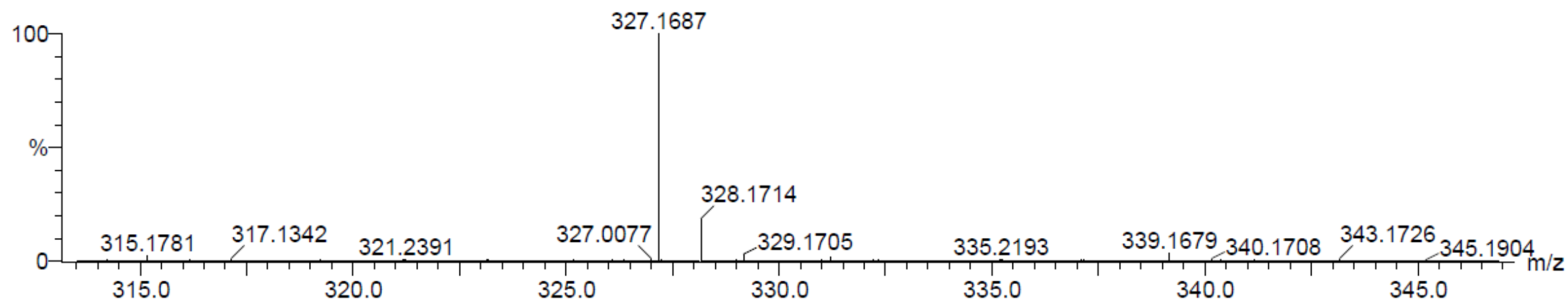

Minimum:				-5.0				
Maximum:		2.0	5.0	150.0				
Mass	Calc. Mass	mDa	PPM	DBE	i-FIT	Norm	Conf(%)	Formula
327.1687	327.1684	0.3	0.9	6.5	3910.3	n/a	n/a	C17 H22 N2 O F3

Figure S23. HRMS spectrum of **3a**.

Monoisotopic Mass, Even Electron Ions
34 formula(e) evaluated with 1 results within limits (up to 50 closest results for each mass)
Elements Used:
C: 0-19 H: 0-30 N: 0-2 O: 0-2 F: 0-3
SD-EC3-424 118 (0.261) Cm (118:154)
1: TOF MS ES+
2.79e+007

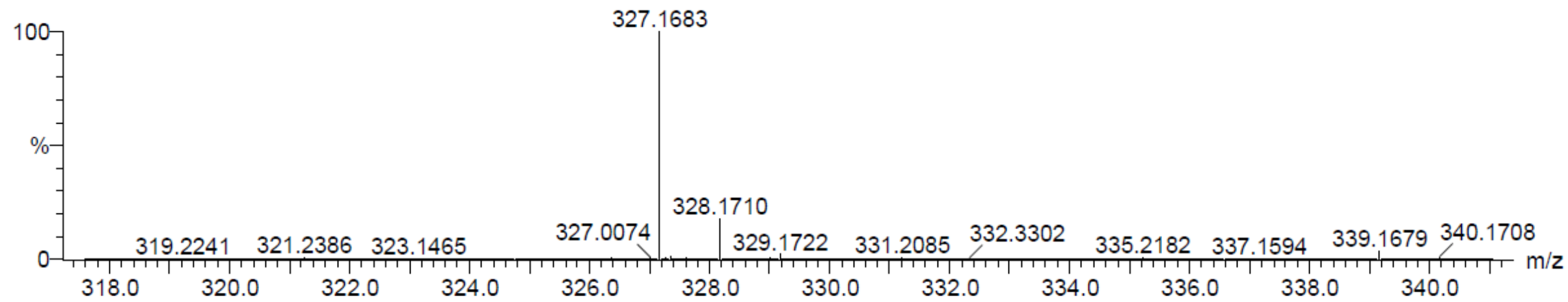

Minimum:				-5.0				
Maximum:		2.0	5.0	150.0				
Mass	Calc. Mass	mDa	PPM	DBE	i-FIT	Norm	Conf(%)	Formula
327.1683	327.1684	-0.1	-0.3	6.5	3458.7	n/a	n/a	C17 H22 N2 O F3

Figure S24. HRMS spectrum of **3b**.

Monoisotopic Mass, Even Electron Ions
412 formula(e) evaluated with 1 results within limits (all results (up to 1000) for each mass)
Elements Used:
C: 0-25 H: 0-35 N: 1-5 O: 0-5 F: 0-6
SD-EC1-085 70 (0.301) Cm (70:100)
1: TOF MS ES+
4.76e+006

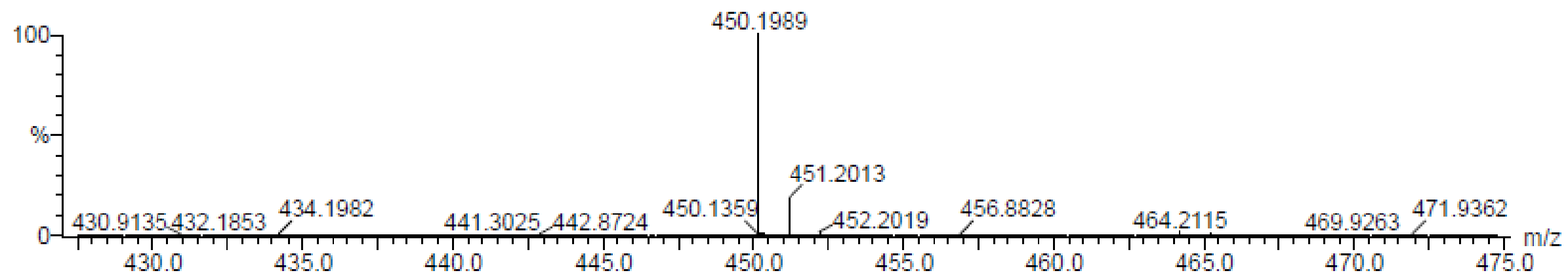

Minimum:				-1.5				
Maximum:		2.0	2.0	50.0				
Mass	Calc. Mass	mDa	PPM	DBE	i-FIT	Norm	Conf(%)	Formula
450.1989	450.1980	0.9	2.0	7.5	956.0	n/a	n/a	C21 H26 N3 O F6

Figure S25. HRMS spectrum of (*S*)-**4a**.

Monoisotopic Mass, Even Electron Ions
90 formula(e) evaluated with 1 results within limits (all results (up to 1000) for each mass)
Elements Used:
C: 1-30 H: 1-30 N: 0-5 O: 0-5 F: 6-6

SD-EC-050-F2 87 (0.376) Cm (85:106)
1: TOF MS ES+

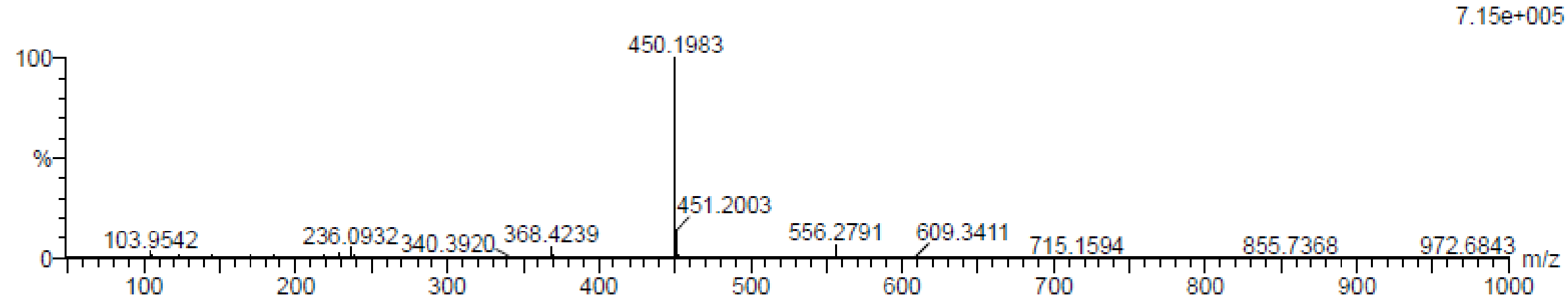

Minimum:				-1.5				
Maximum:		2.0	5.0	50.0				
Mass	Calc. Mass	mDa	PPM	DBE	i-FIT	Norm	Conf(%)	Formula
450.1983	450.1980	0.3	0.7	7.5	638.2	n/a	n/a	C21 H26 N3 O F6

Figure S26. HRMS spectrum of (*R*)-**4a**.

Monoisotopic Mass, Even Electron Ions
37 formula(e) evaluated with 1 results within limits (up to 50 closest results for each mass)
Elements Used:
C: 0-25 H: 0-30 N: 0-5 O: 0-2 F: 6-6
SD-EC1-064 87 (0.376) Cm (87:103)

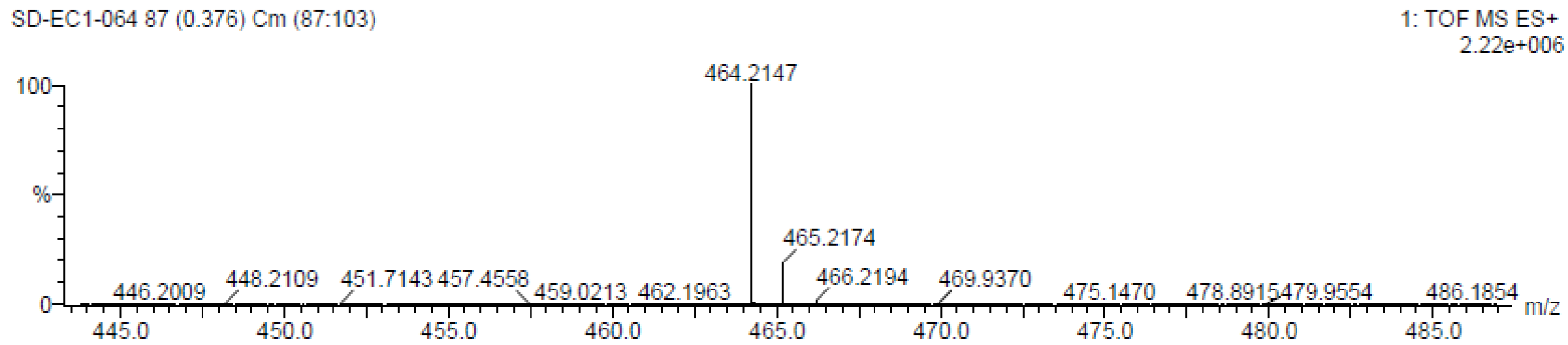

Minimum:				-5.0				
Maximum:		2.0	5.0	150.0				
Mass	Calc. Mass	mDa	PPM	DBE	i-FIT	Norm	Conf(%)	Formula
464.2147	464.2137	1.0	2.2	7.5	1809.8	n/a	n/a	C22 H28 N3 O F6

Figure S27. HRMS spectrum of (*S*)-**4b**.

Monoisotopic Mass, Even Electron Ions
205 formula(e) evaluated with 1 results within limits (all results (up to 1000) for each mass)
Elements Used:
C: 0-25 H: 0-35 N: 1-5 O: 0-3 F: 0-6
SD-EC1-084 69 (0.297) Cm (69:84)
1: TOF MS ES+

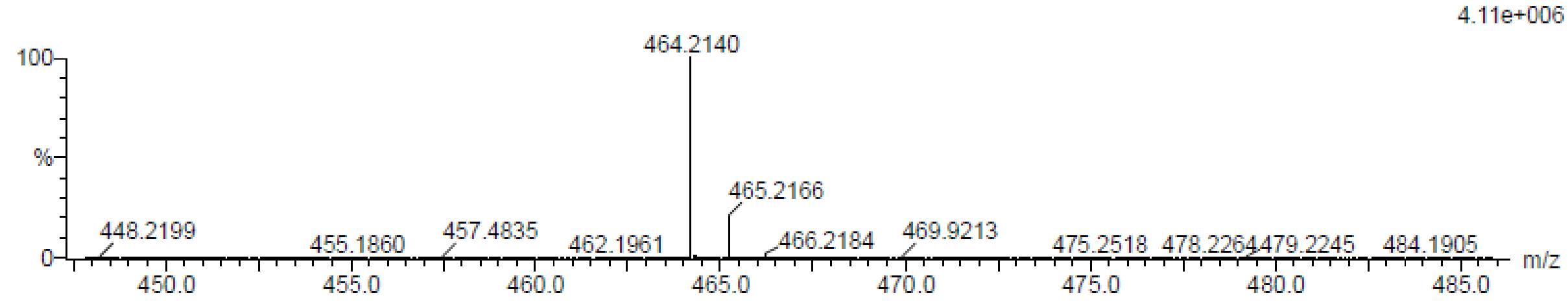

Minimum:				-1.5				
Maximum:		2.0	2.0	50.0				
Mass	Calc. Mass	mDa	PPM	DBE	i-FIT	Norm	Conf(%)	Formula
464.2140	464.2137	0.3	0.6	7.5	888.1	n/a	n/a	C22 H28 N3 O F6

Figure S28. HRMS spectrum of (*R*)-**4b**.

Monoisotopic Mass, Even Electron Ions
321 formula(e) evaluated with 1 results within limits (all results (up to 1000) for each mass)
Elements Used:
C: 0-25 H: 0-35 N: 1-5 O: 0-5 F: 0-6
SD-EC1-073 87 (0.376) Cm (79:89)
1: TOF MS ES+

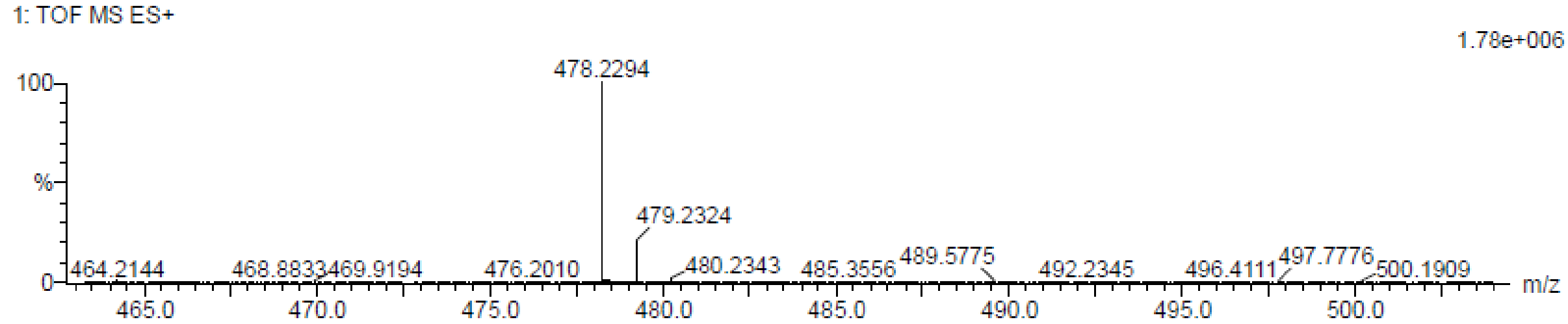

Minimum:				-1.5				
Maximum:		2.0	2.0	50.0				
Mass	Calc. Mass	mDa	PPM	DBE	i-FIT	Norm	Conf(%)	Formula
478.2294	478.2293	0.1	0.2	7.5	700.7	n/a	n/a	C23 H30 N3 O F6

Figure S29. HRMS spectrum of (*S*)-**4c**.

Monoisotopic Mass, Even Electron Ions
321 formula(e) evaluated with 1 results within limits (all results (up to 1000) for each mass)
Elements Used:
C: 0-25 H: 0-35 N: 1-5 O: 0-5 F: 0-6
SD-EC1-082 50 (0.225) Cm (49:67)
1: TOF MS ES+

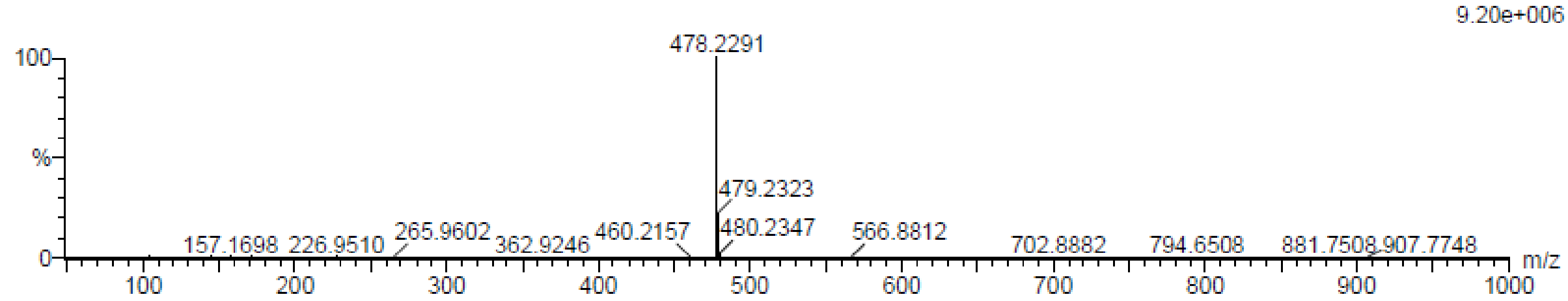

Minimum:				-1.5				
Maximum:		2.0	2.0	50.0				
Mass	Calc. Mass	mDa	PPM	DBE	i-FIT	Norm	Conf(%)	Formula
478.2291	478.2293	-0.2	-0.4	7.5	992.0	n/a	n/a	C23 H30 N3 O F6

Figure S30. HRMS spectrum of (*R*)-**4c**.

Monoisotopic Mass, Even Electron Ions
281 formula(e) evaluated with 1 results within limits (all results (up to 1000) for each mass)
Elements Used:
C: 0-25 H: 0-35 N: 1-5 O: 0-5 F: 0-6
SD-EC1-083 (0.029) Is (1.00,1.00) C24H32F6N3O
1: TOF MS ES+

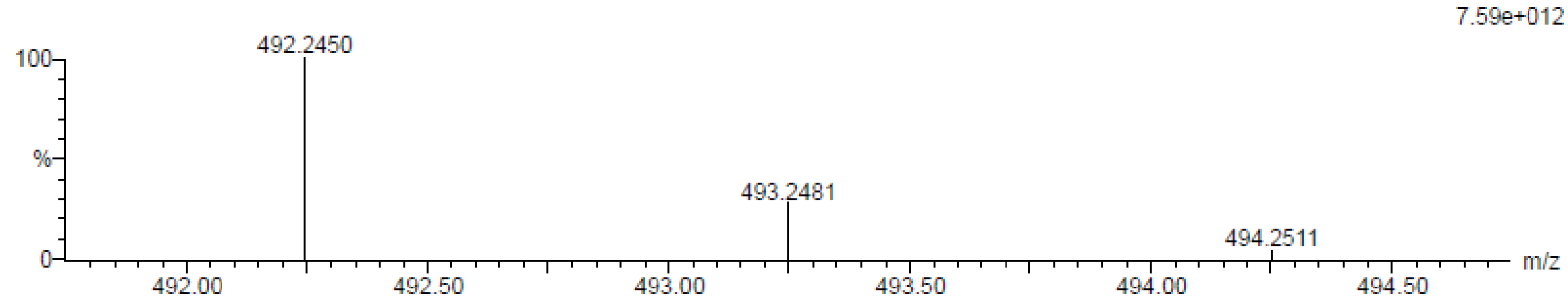

Minimum:				-1.5				
Maximum:		2.0	2.0	50.0				
Mass	Calc. Mass	mDa	PPM	DBE	i-FIT	Norm	Conf(%)	Formula
492.2450	492.2450	0.0	0.0	7.5	43.8	n/a	n/a	C24 H32 N3 O F6

Figure S31. HRMS spectrum of (*S*)-**4d**.

Monoisotopic Mass, Even Electron Ions
156 formula(e) evaluated with 1 results within limits (all results (up to 1000) for each mass)
Elements Used:
C: 0-25 H: 0-35 N: 1-5 O: 0-3 F: 0-6
SD-EC1-086 71 (0.305) Cm (70:83)
1: TOF MS ES+

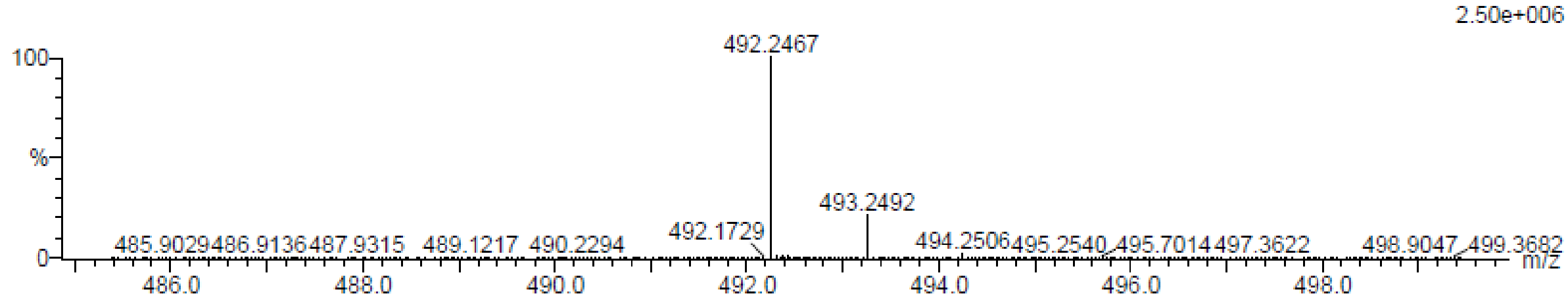

Minimum:				-1.5				
Maximum:		2.0	5.0	50.0				
Mass	Calc. Mass	mDa	PPM	DBE	i-FIT	Norm	Conf(%)	Formula
492.2467	492.2450	1.7	3.5	7.5	768.0	n/a	n/a	C24 H32 N3 O F6

Figure S32. HRMS spectrum of (*R*)-**4d**.

Monoisotopic Mass, Even Electron Ions
67 formula(e) evaluated with 1 results within limits (up to 50 closest results for each mass)
Elements Used:
C: 0-30 H: 0-30 N: 1-5 O: 1-5 F: 6-6
SD-EC-2-196B 130 (0.550) Cm (112:144)
1: TOF MS ES+

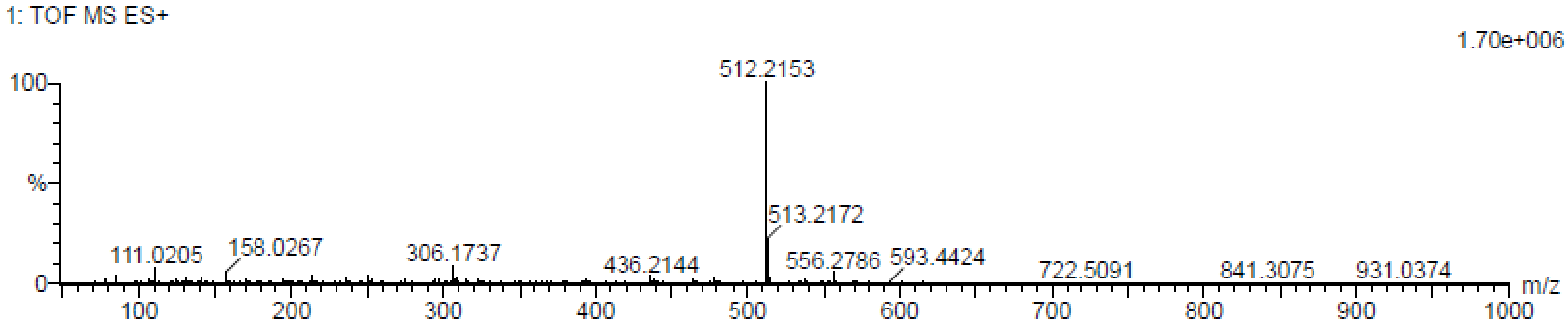

Minimum:				-1.5				
Maximum:		2.0	4.0	50.0				
Mass	Calc. Mass	mDa	PPM	DBE	i-FIT	Norm	Conf(%)	Formula
512.2153	512.2137	1.6	3.1	11.5	940.2	n/a	n/a	C26 H28 N3 O F6

Figure S33. HRMS spectrum of (*S*)-**4e**.

Monoisotopic Mass, Even Electron Ions
170 formula(e) evaluated with 1 results within limits (up to 50 closest results for each mass)
Elements Used:
C: 0-30 H: 0-35 N: 0-5 O: 0-2 F: 0-6
SD-EC1-182 54 (0.240) Cm (54:67)
1: TOF MS ES+

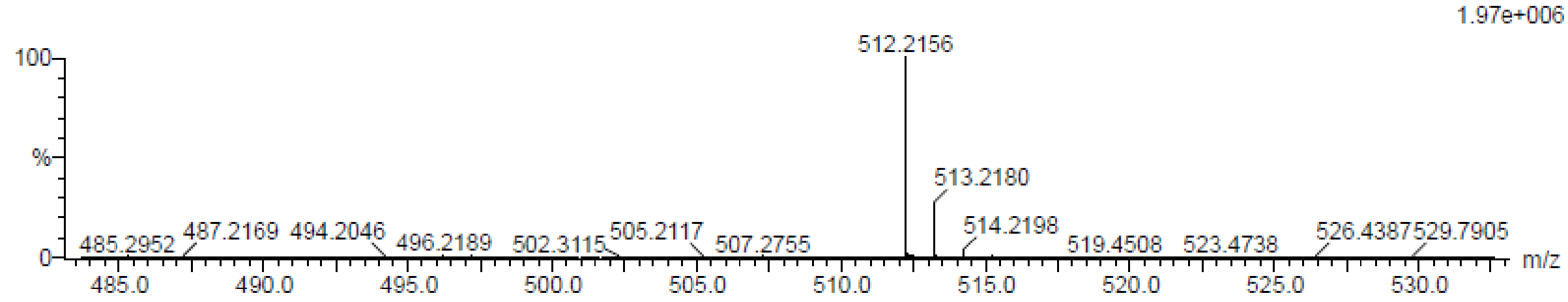

Minimum:				-1.5				
Maximum:		2.0	5.0	50.0				
Mass	Calc. Mass	mDa	PPM	DBE	i-FIT	Norm	Conf(%)	Formula
512.2156	512.2137	1.9	3.7	11.5	864.3	n/a	n/a	C26 H28 N3 O F6

Figure S34. HRMS spectrum of (*R*)-**4e**.

Monoisotopic Mass, Even Electron Ions
122 formula(e) evaluated with 1 results within limits (up to 50 closest results for each mass)
Elements Used:
C: 0-25 H: 0-30 N: 0-5 O: 0-2 F: 0-6
SD-EC1-103 88 (0.204) Cm (87:129)
1: TOF MS ES+

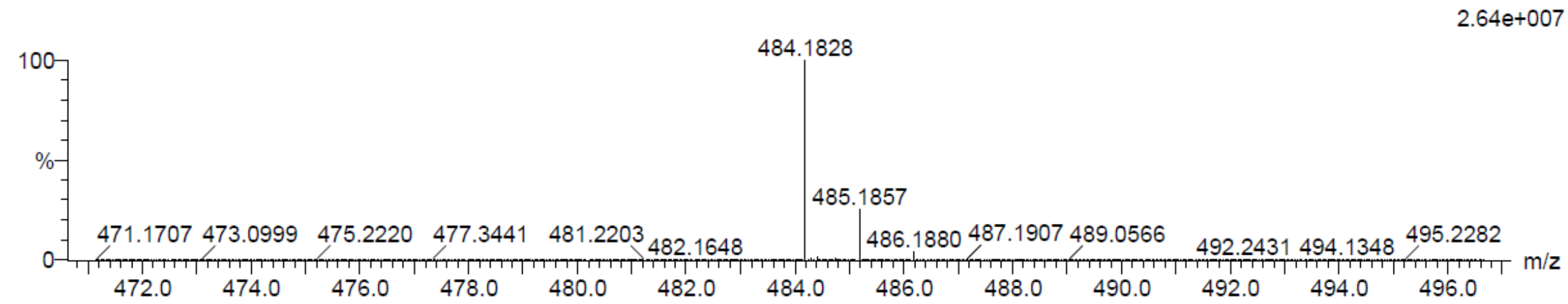

Minimum:				-1.5				
Maximum:		1.0	5.0	50.0				
Mass	Calc. Mass	mDa	PPM	DBE	i-FIT	Norm	Conf(%)	Formula
484.1828	484.1824	0.4	0.8	11.5	1159.5	n/a	n/a	C24 H24 N3 O F6

Figure S35. HRMS spectrum of (*S*)-**4f**.

Monoisotopic Mass, Even Electron Ions
168 formula(e) evaluated with 1 results within limits (all results (up to 1000) for each mass)
Elements Used:
C: 0-25 H: 0-35 N: 1-5 O: 0-3 F: 0-6
SD-EC1-115 112 (0.472) Cm (108:129)
1: TOF MS ES+

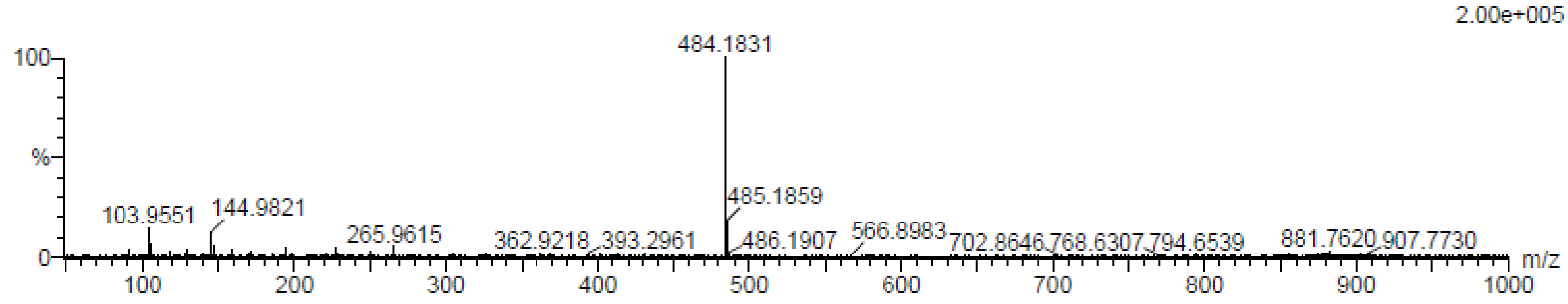

Minimum:				-1.5				
Maximum:		2.0	2.0	50.0				
Mass	Calc. Mass	mDa	PPM	DBE	i-FIT	Norm	Conf(%)	Formula
484.1831	484.1824	0.7	1.4	11.5	615.0	n/a	n/a	C24 H24 N3 O F6

Figure S36. HRMS spectrum of (*R*)-**4f**.

S5. HPLC chromatograms of compounds 1-4a-f.

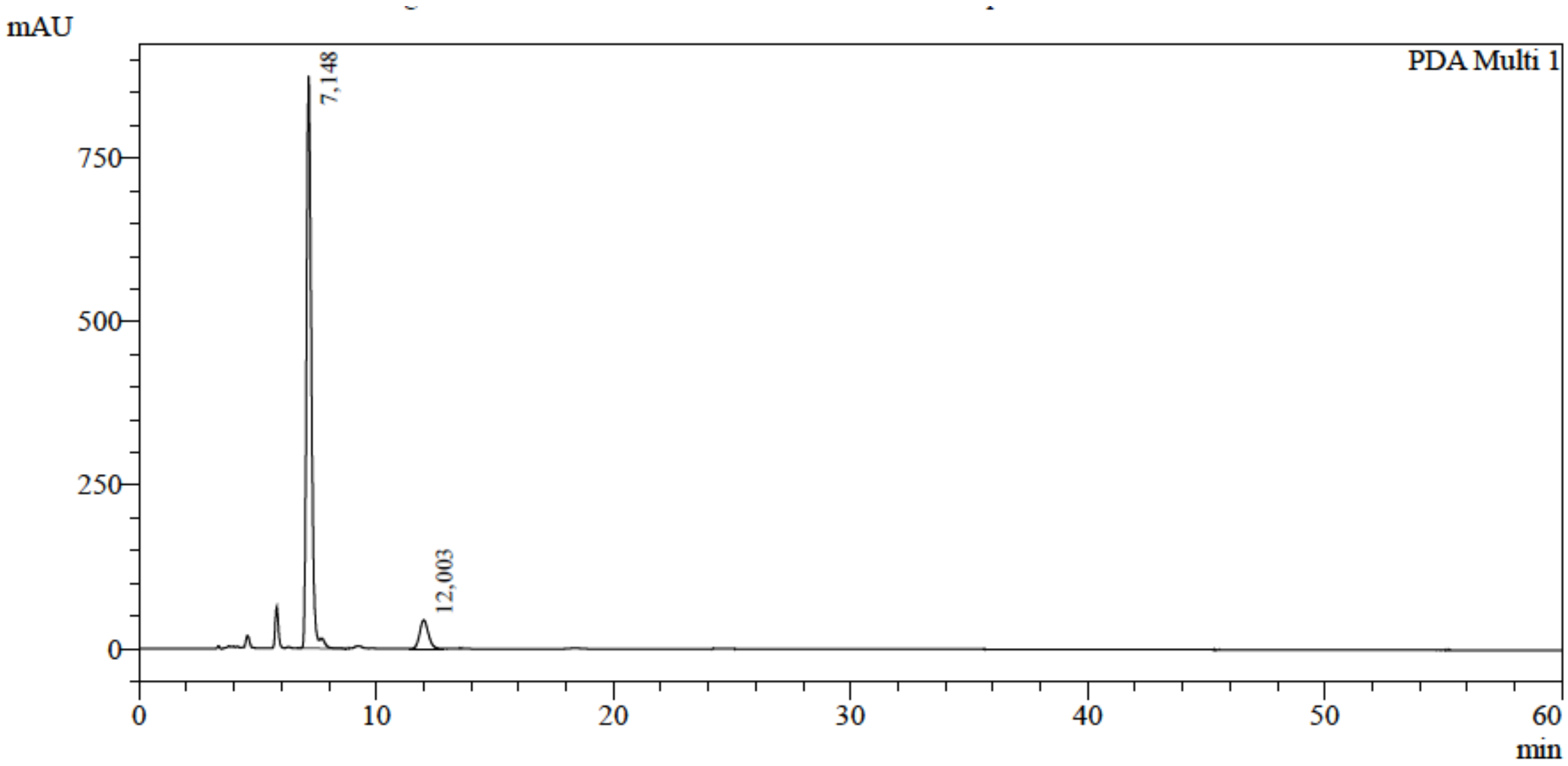

Peak#	Ret. Time	Area	Area %
1	7,148	12509364	91,882
2	12,003	1105278	8,118
Total		13614643	100,000

Figure S37. HPLC chromatogram of (*S*)-**2**.

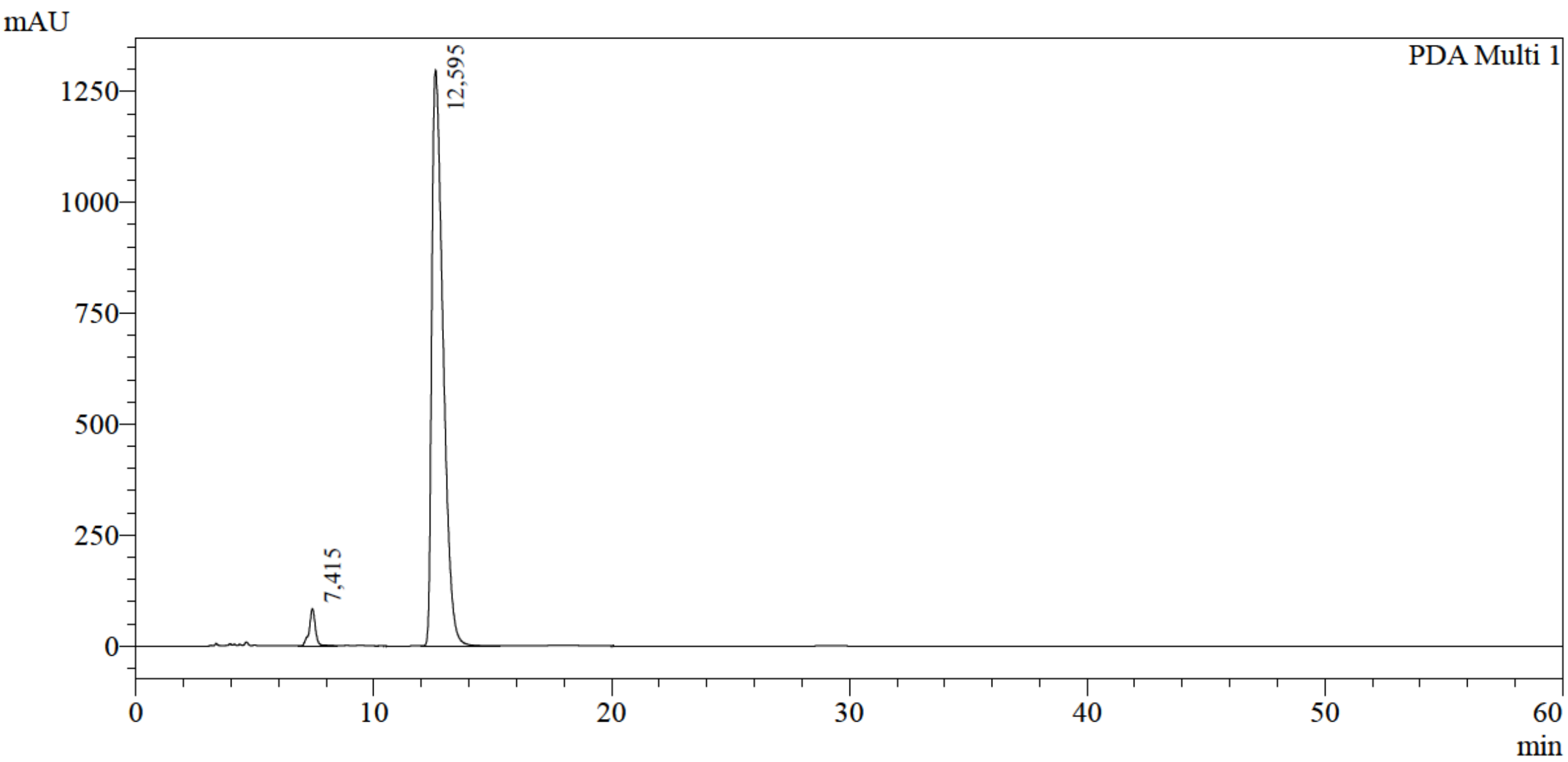

Peak#	Ret. Time	Area	Area %
1	7,415	1524830	3,436
2	12,595	42847590	96,564
Total		44372420	100,000

Figure S38. HPLC chromatogram of (*R*)-**2**.

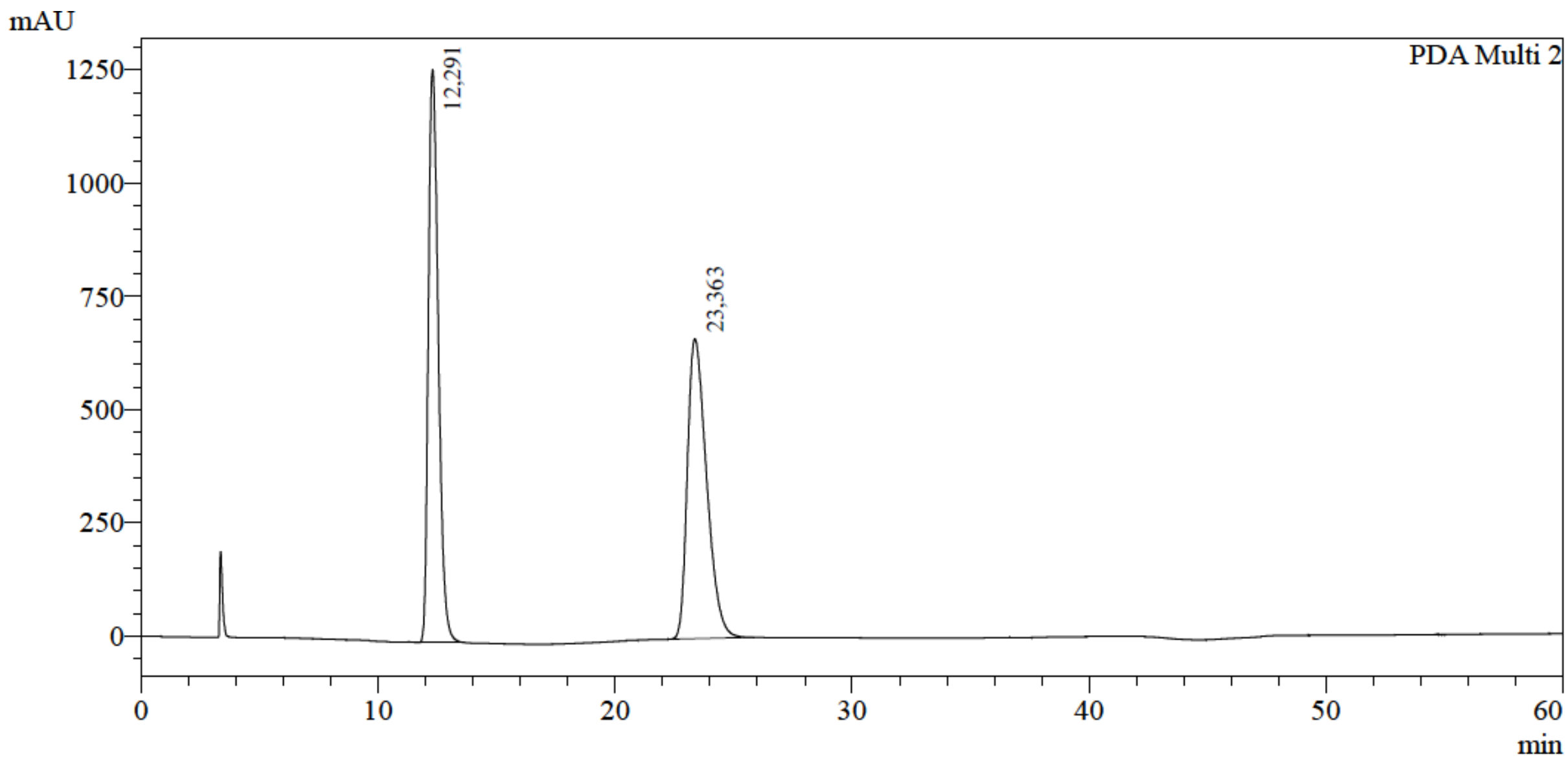

Peak#	Ret. Time	Area	Area %
1	12,291	37018072	49,797
2	23,363	37320296	50,203
Total		74338368	100,000

Figure S39. HPLC chromatogram of (*S*)-**3**.

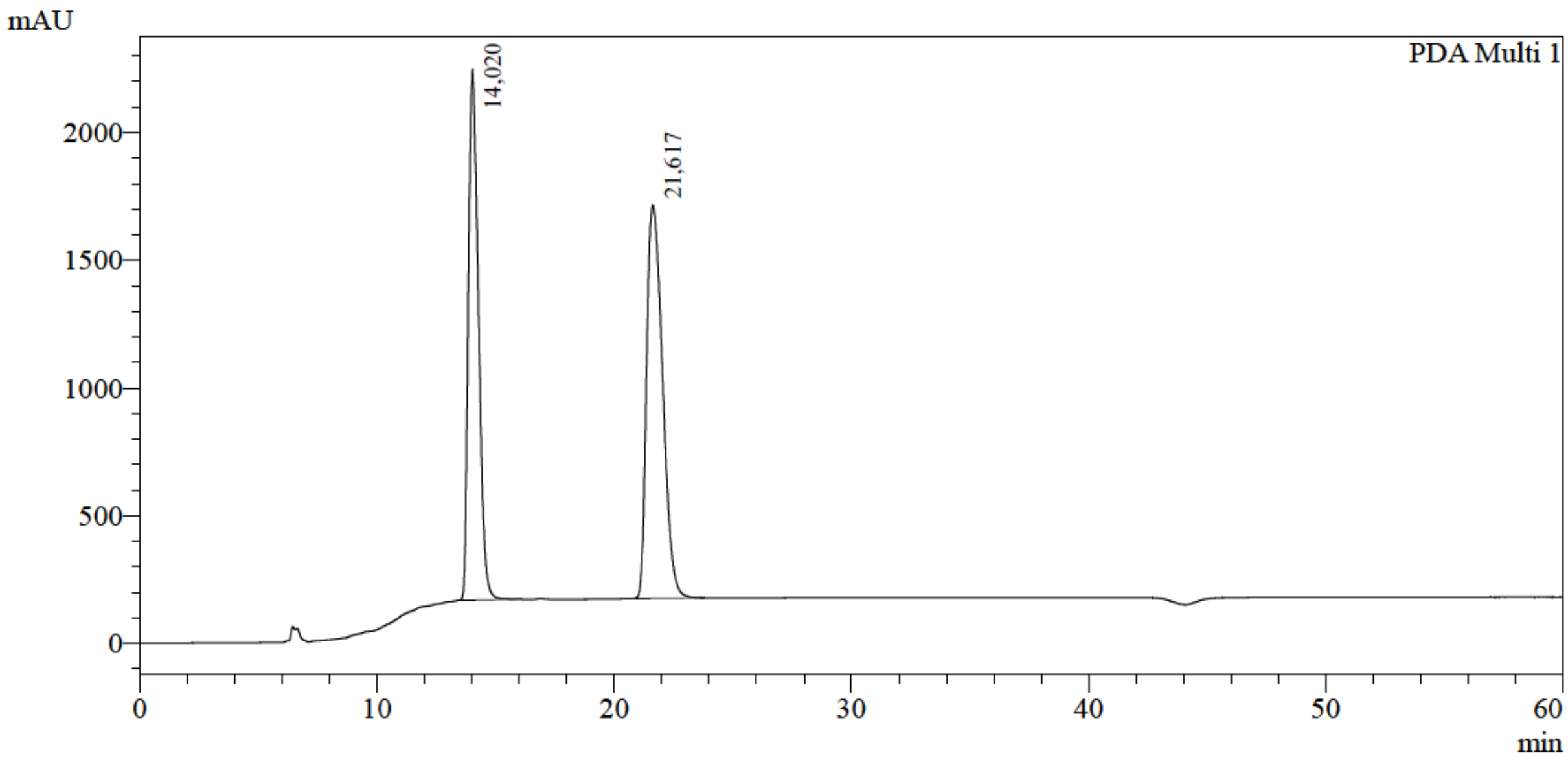

Peak#	Ret. Time	Area	Area %
1	14,020	61841610	46,174
2	21,617	72089510	53,826
Total		133931120	100,000

Figure S40. HPLC chromatogram of (*R*)-**3**.

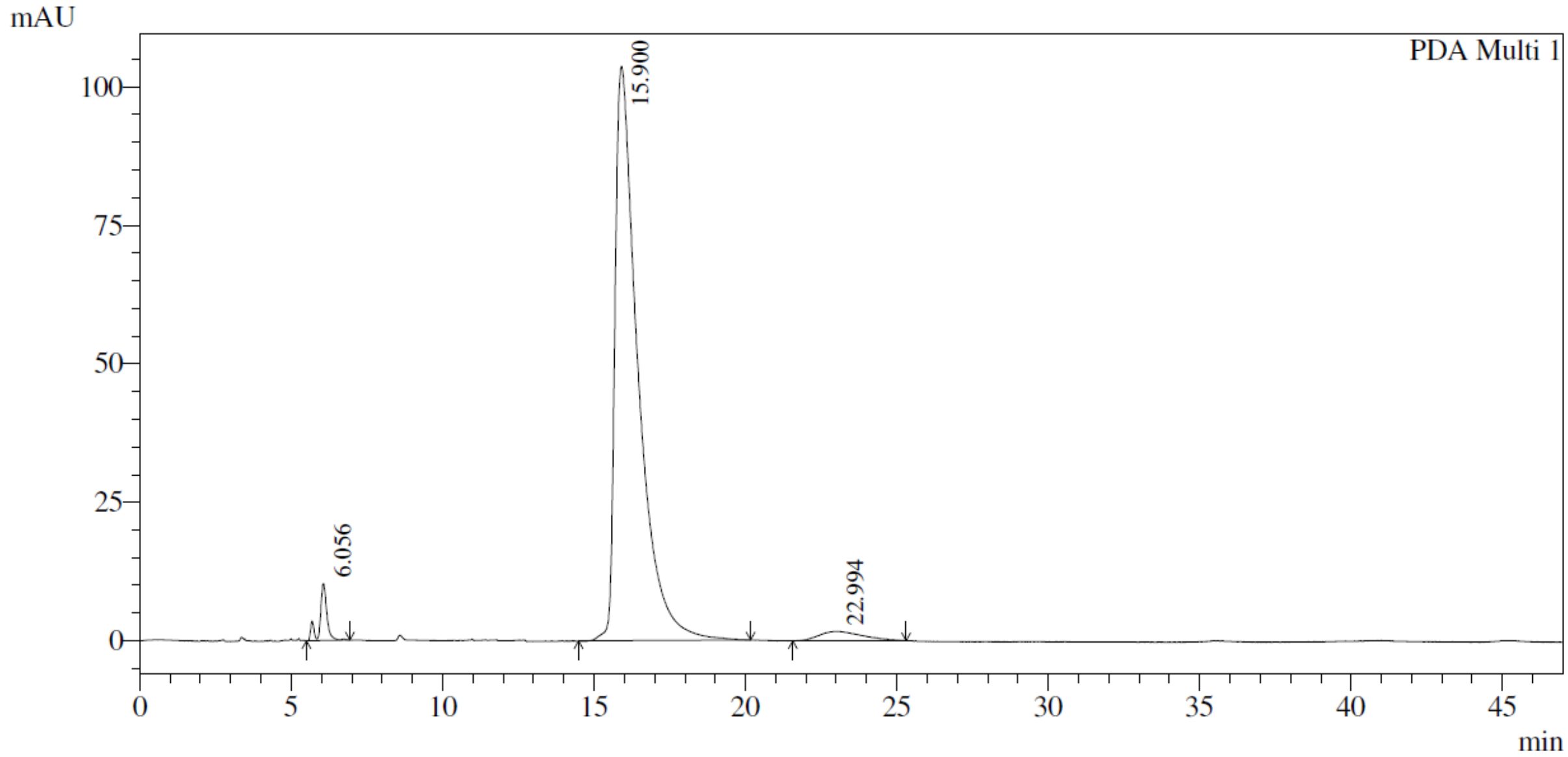

Ret. Time	Area	Height	Area %	Height %
6.056	170099	10298	2.923	8.905
15.900	5481221	103667	94.205	89.644
22.994	167082	1678	2.872	1.451
	5818401	115642	100.000	100.000

Figure S41. HPLC chromatogram of (*S*)-**4a**.

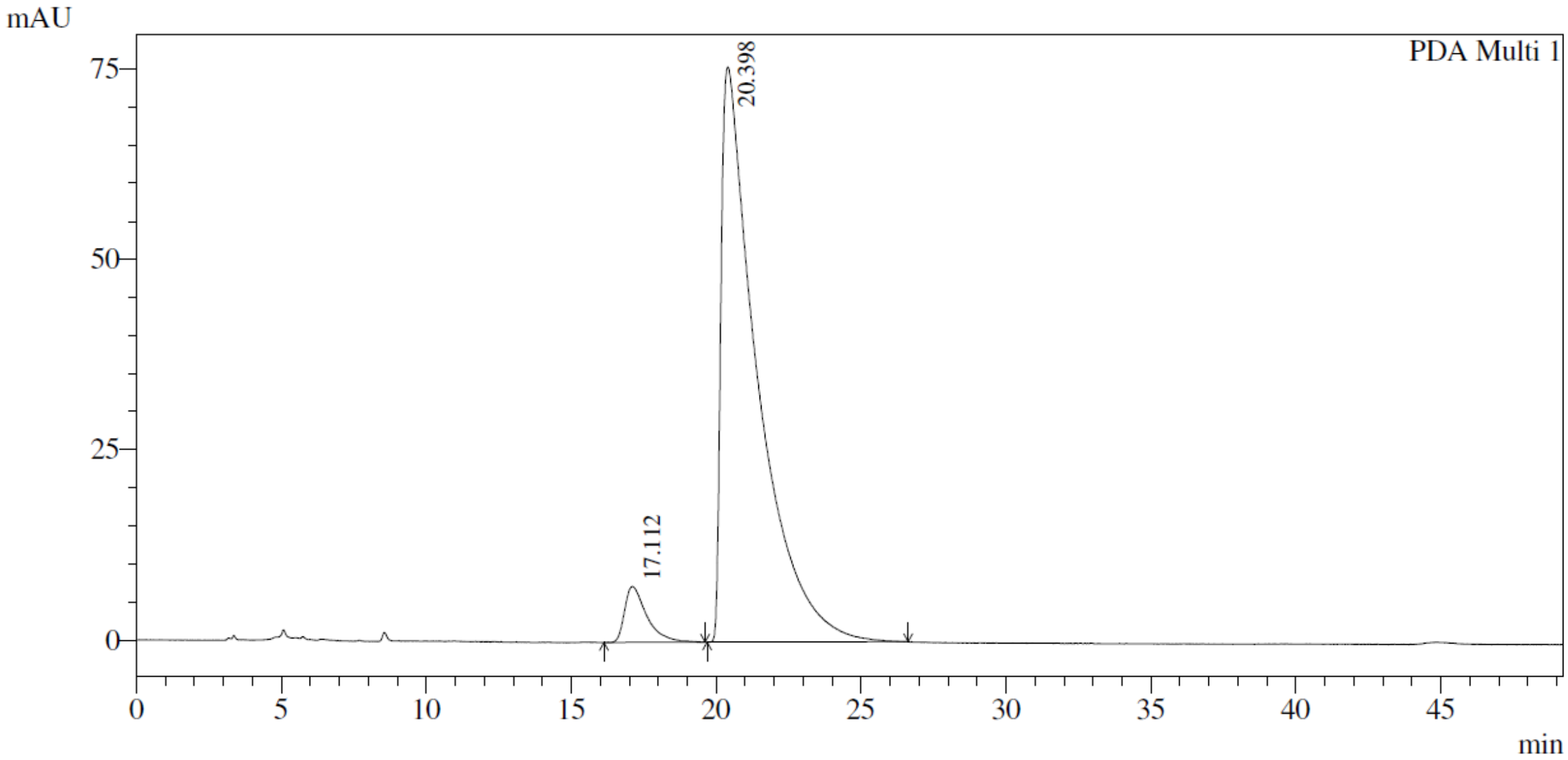

Ret. Time	Area	Height	Area %	Height %
17.112	391016	7348	5.614	8.862
20.398	6573533	75564	94.386	91.138
	6964549	82912	100.000	100.000

Figure S42. HPLC chromatogram of (*R*)-**4a**.

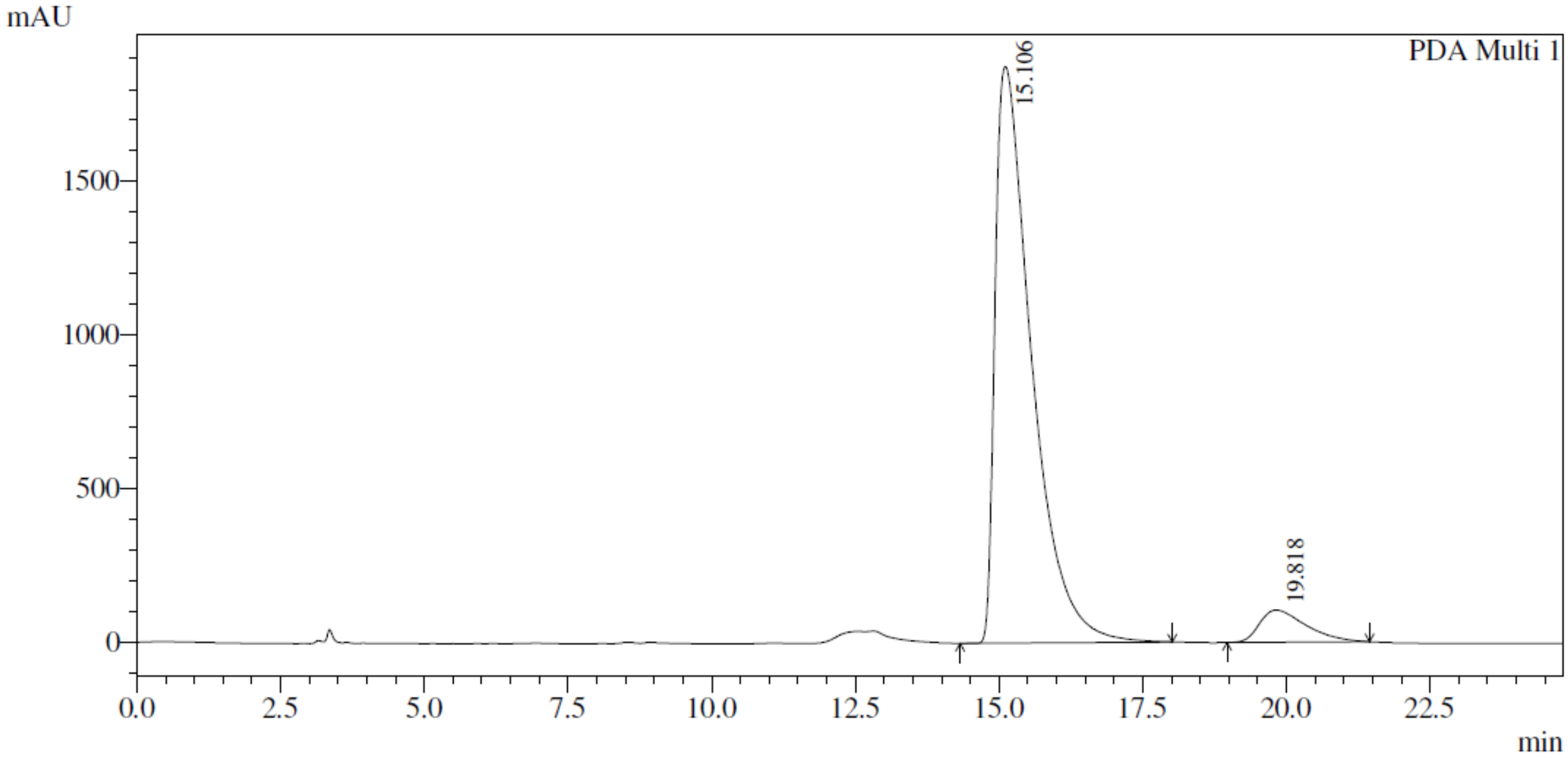

Ret. Time	Area	Height	Area %	Height %
15.106	83037517	1876125	93.337	94.708
19.818	5927916	104831	6.663	5.292
	88965433	1980955	100.000	100.000

Figure S43. HPLC chromatogram of (*S*)-**4b**.

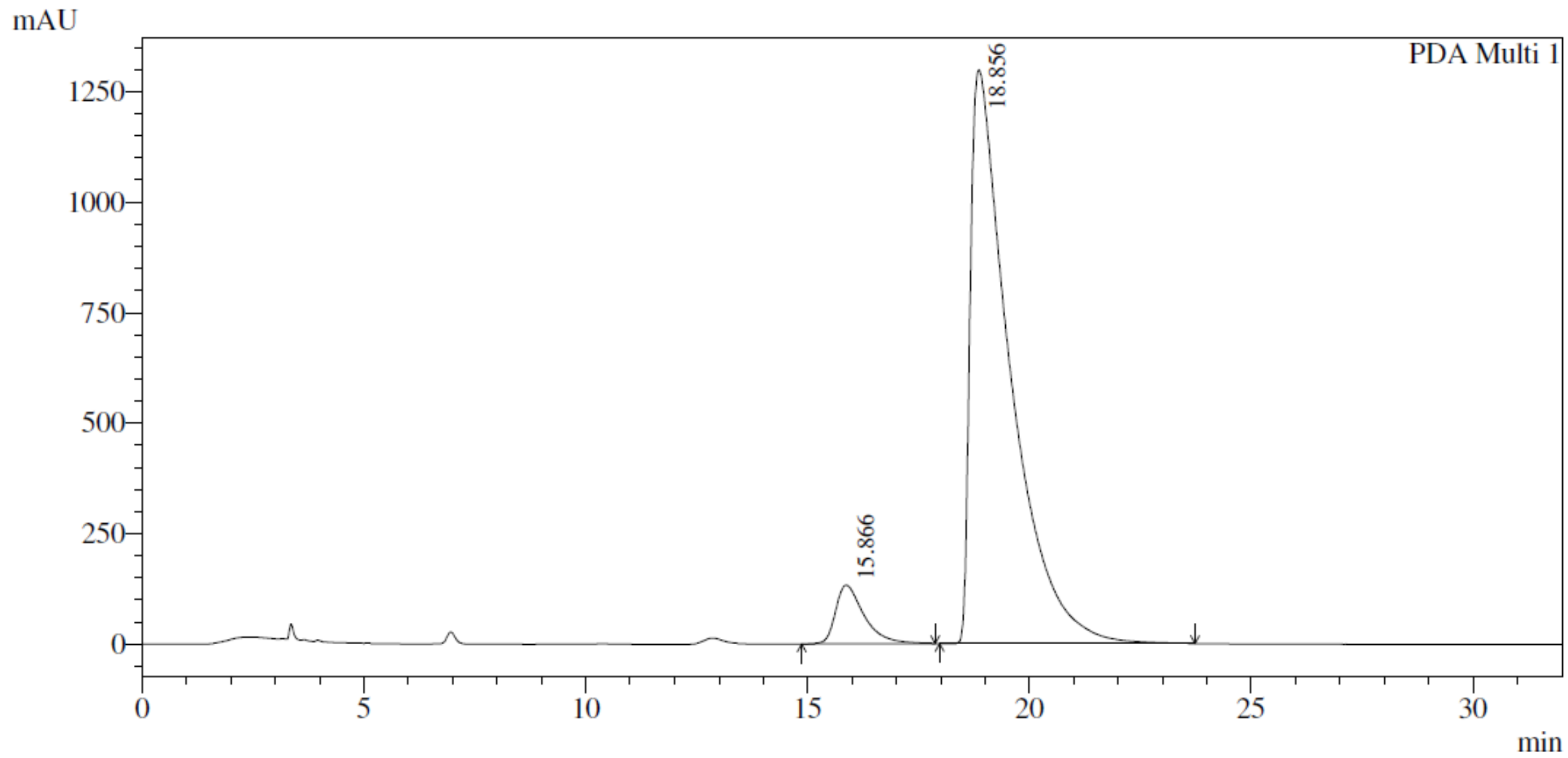

Ret. Time	Area	Height	Area %	Height %
15.866	5915529	132547	6.783	9.264
18.856	81301635	1298260	93.217	90.736
	87217164	1430807	100.000	100.000

Figure S44. HPLC chromatogram of (*R*)-**4b**.

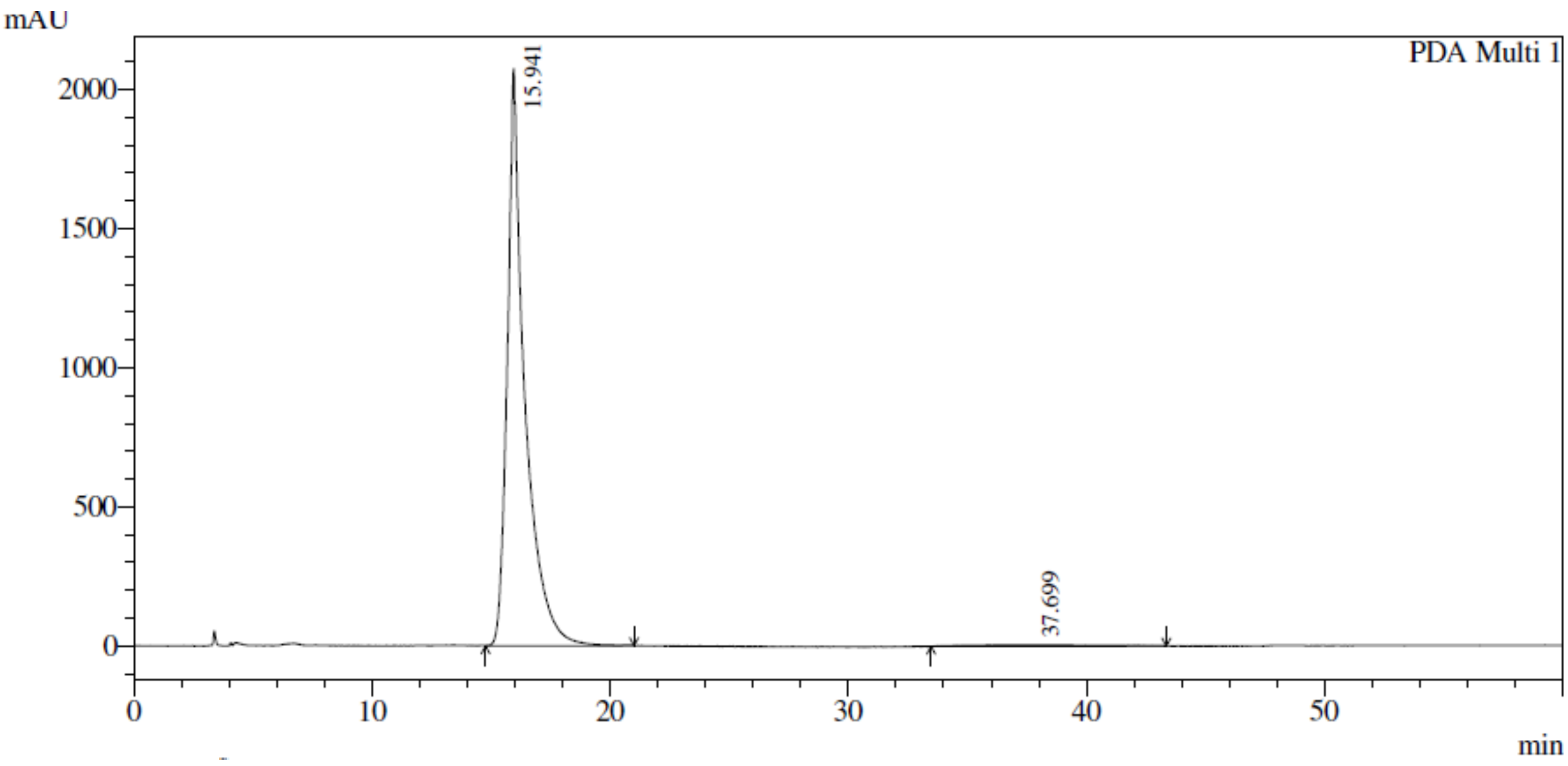

Ret. Time	Area	Height	Area %	Height %
15.941	106075834	2074515	98.410	99.719
37.699	1714387	5850	1.590	0.281
	107790221	2080365	100.000	100.000

Figure S45. HPLC chromatogram of (*S*)-**4c**.

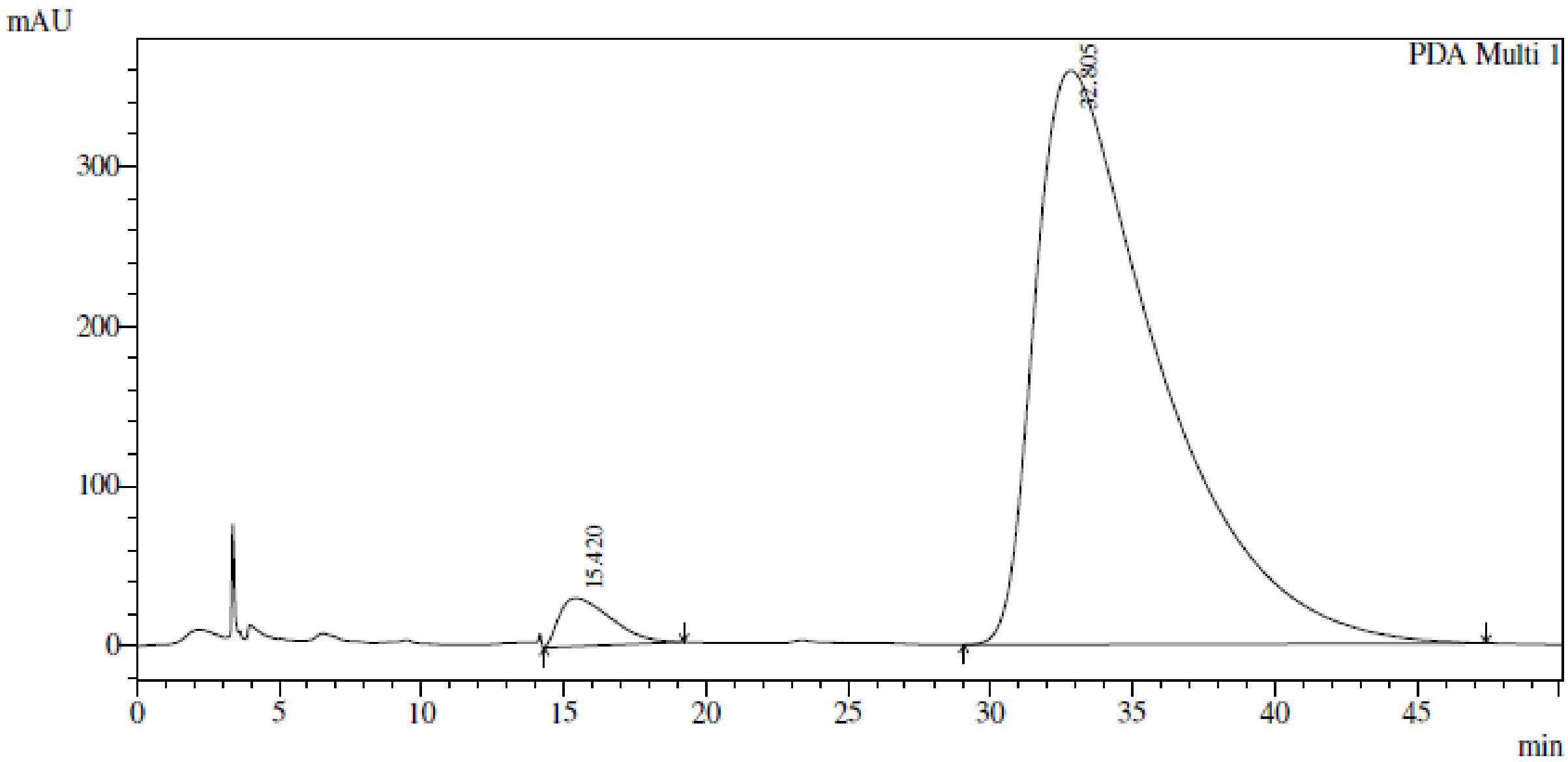

Ret. Time	Area	Height	Area %	Height %
15.420	3807436	30015	3.361	7.720
32.805	109491229	358777	96.639	92.280
	113298665	388792	100.000	100.000

Figure S46. HPLC chromatogram of (*R*)-**4c**.

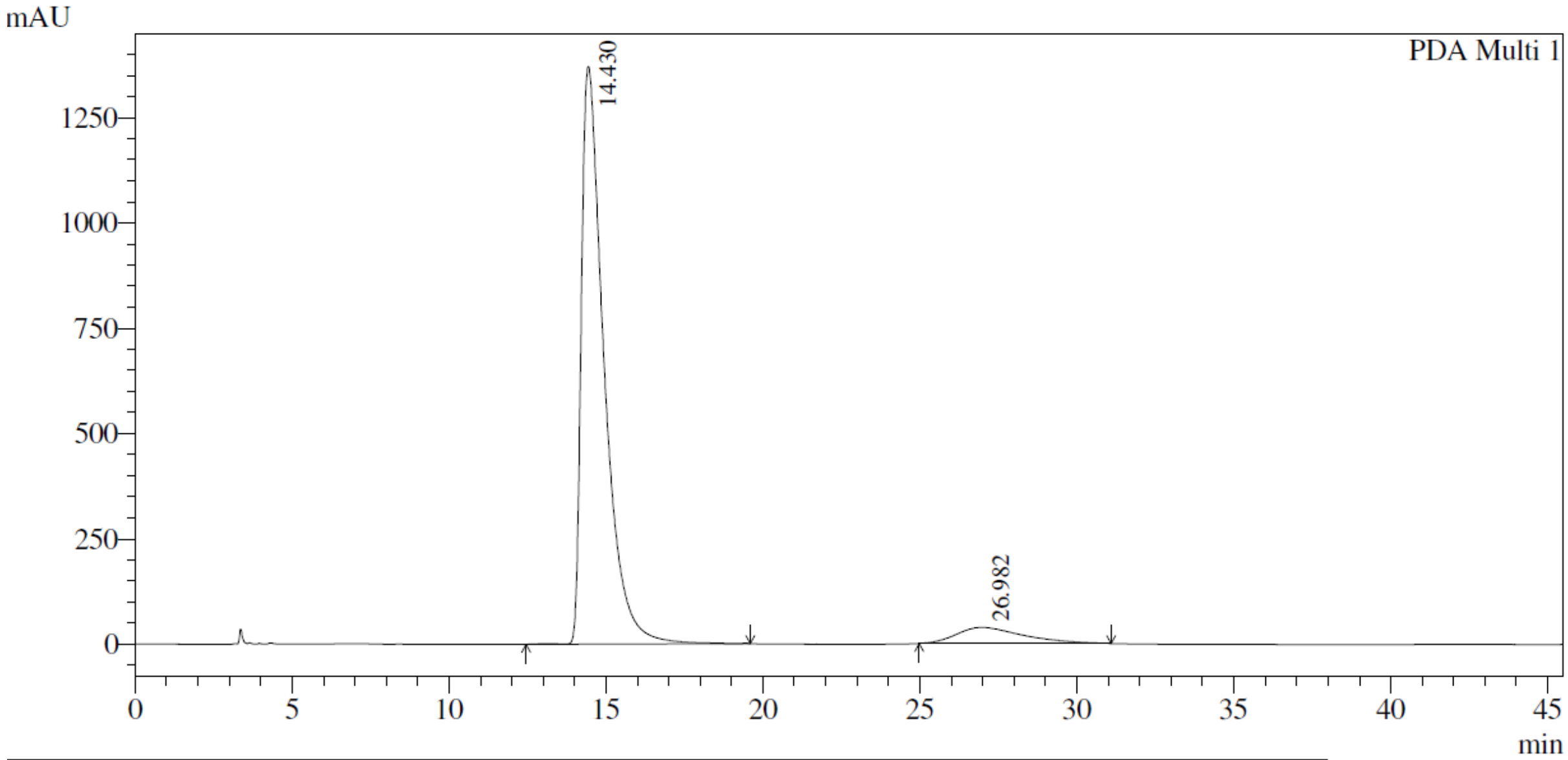

Ret. Time	Area	Height	Area %	Height %
14.430	68988206	1371379	92.490	97.304
26.982	5601420	38002	7.510	2.696
	74589625	1409380	100.000	100.000

Figure S47. HPLC chromatogram of (*S*)-**4d**.

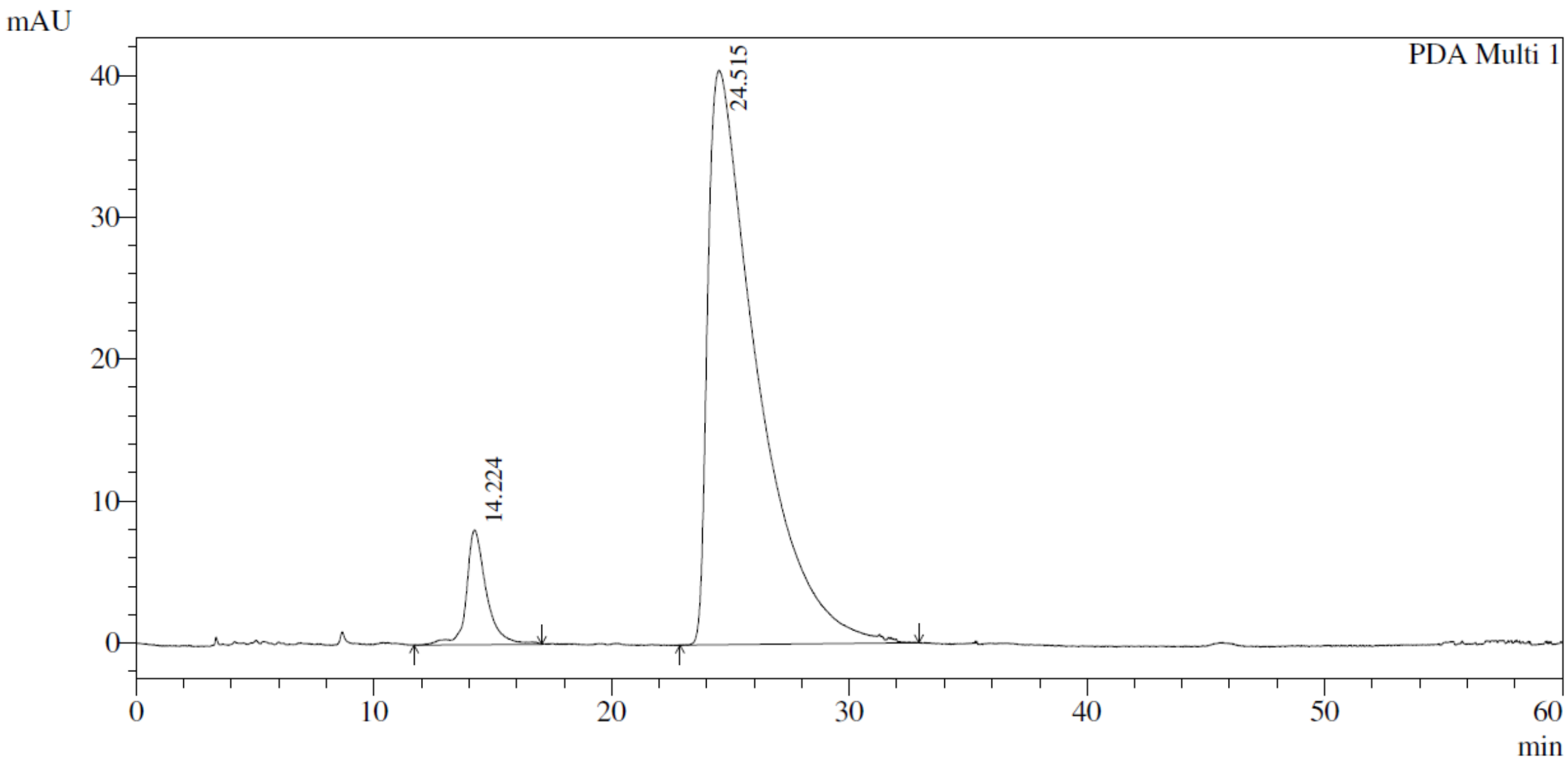

Ret. Time	Area	Height	Area %	Height %
14.224	471423	8089	7.460	16.656
24.515	5847866	40473	92.540	83.344
	6319289	48562	100.000	100.000

Figure S48. HPLC chromatogram of (*R*)-**4d**.

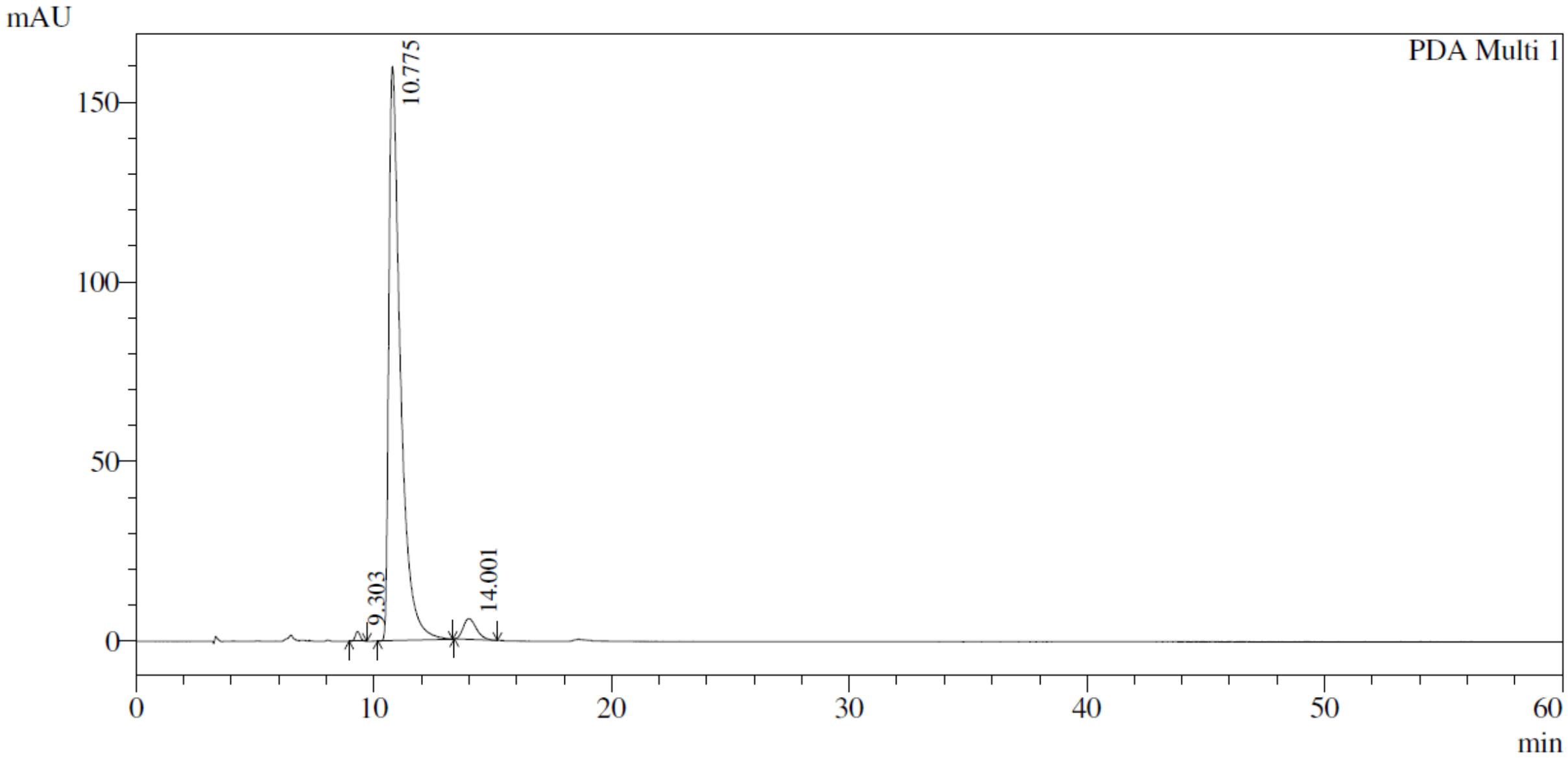

Ret. Time	Area	Height	Area %	Height %
9.303	39743	2731	0.697	1.622
10.775	5433301	159786	95.337	94.909
14.001	225978	5840	3.965	3.469
	5699022	168357	100.000	100.000

Figure S49. HPLC chromatogram of (*S*)-**4e**.

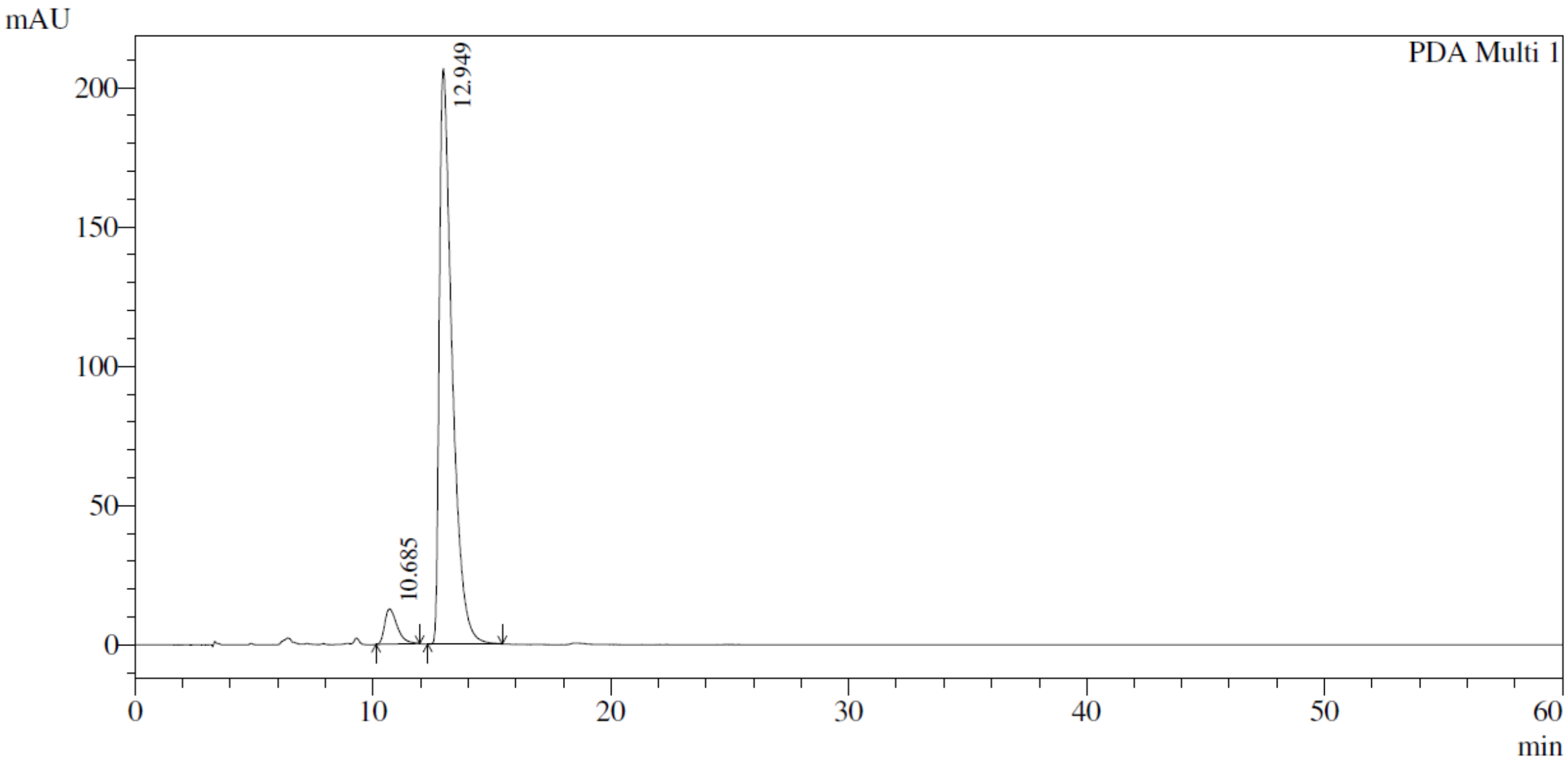

Ret. Time	Area	Height	Area %	Height %
10.685	461527	12647	5.629	5.766
12.949	7737682	206682	94.371	94.234
	8199209	219329	100.000	100.000

Figure S50. HPLC chromatogram of (*R*)-**4e**.

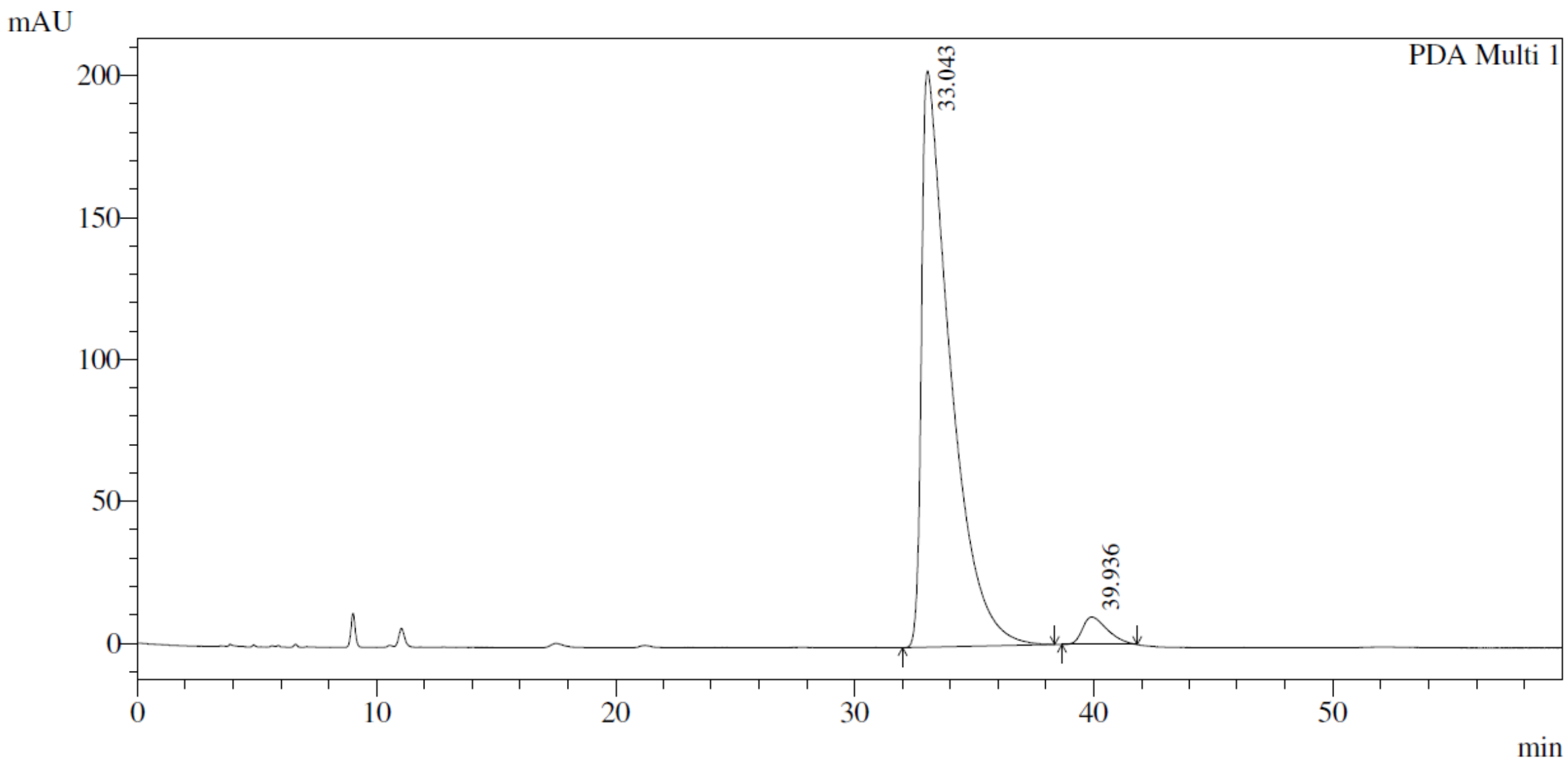

Ret. Time	Area	Height	Area %	Height %
33.043	17065110	203106	95.993	95.439
39.936	712357	9705	4.007	4.561
	17777467	212812	100.000	100.000

Figure S51. HPLC chromatogram of (*S*)-**4f**.

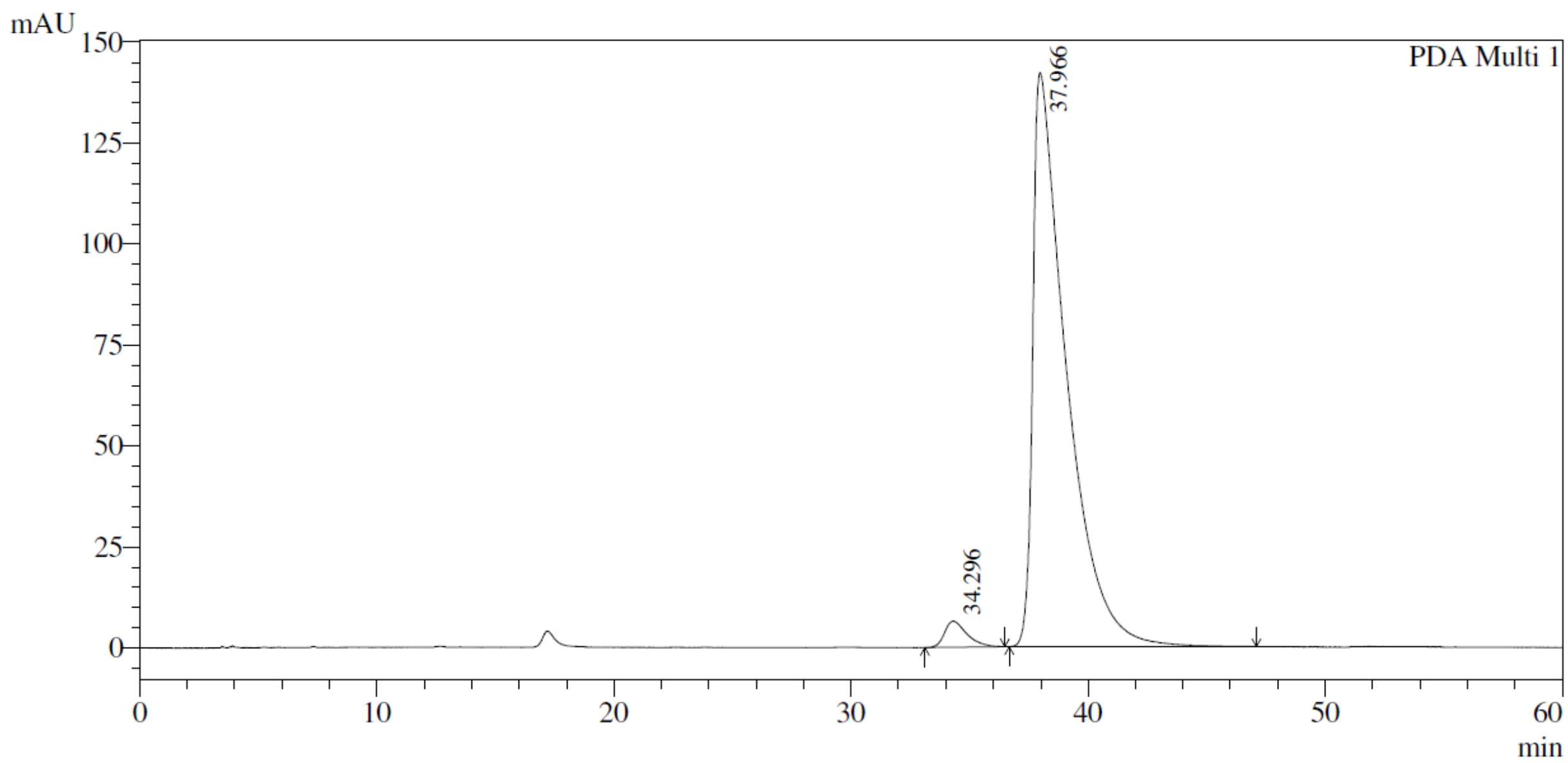

Ret. Time	Area	Height	Area %	Height %
34.296	424002	6501	2.938	4.373
37.966	14006428	142171	97.062	95.627
	14430429	148672	100.000	100.000

Figure S52. HPLC chromatogram of (*R*)-**4f**.

S6. Physicochemical and pharmacokinetic properties.

SiriusT3™ procedures

Materials. Chemical reagents and solvents were obtained from commercial suppliers and used without further purification.

Calibration tests. The cleaning test was designed to clean the entire length of the probes to prevent sample contamination. Cleaning was performed at the beginning and after the logP determination. The blank test was performed to calibrate the pH electrode and standardize the pH metric determinations. The potassium hydrogen phthalate (KHP) determination was standardized to determine the concentration of the basic titrant (KOH). The tris(hydroxymethyl)aminomethane determination was used to experimentally determine the concentration of the acidic titrant (HCl).

Assay prediction. A sample model was created considering the weighed product mass, its molar mass, the predicted pKa and logP values in the neutral form by Schrödinger and Pallas software, and its acidic or basic character. Once the sample model was created, the Assay Design tab displays the ionization profile and the species distribution diagram as a function of the predicted pH. The minimum quantity of compounds to be weighed was estimated from the buffer index profile graph using a pH-metric assay.

Determination of pKa, logP, and logD. All parameters were run with SiriusT3Control. Three pKa values were measured in water/methanol mixtures of 55/45, 45/55, and 35/65, respectively. All results were observed with SiriusT3Refine. Using the Yasuda-Shedlovsky method, linear extrapolation lines were constructed, yielding the true thermodynamic pKa values (pKa0). These values were then used to plot species distribution diagrams as a function of pH. The lipophilicity profile was determined by the potentiometric method in a two-phase water/octanol system. The evolution of the distribution of the compound between the two phases as a function of pH was observed and led to the obtaining of the logP and logD at physiological pH.

S7. *In vitro* antimycobacterial activity.

Materials. Mueller-Hinton (MH) medium was purchased from Millipore (Sigma-Aldrich), whereas cation-adjusted MH broth (CAMHB) and CLR were obtained from Sigma-Aldrich. Oleic acid-dextrose-catalase (OADC) was prepared in-house (NaCl, bovine serum albumin (BSA), dextrose, catalase and oleic acid were sourced from Sigma-Aldrich). The *M. avium* strain ATCC 700898 (or "MAC 101") was purchased from the American Type Culture Collection (ATCC) (Manassas, Virginia, USA). *M. xenopi* DSM43995 and *M. abscessus* subsp. *abscessus* DSM44196 were obtained from the Deutsche Sammlung für Mikroorganismen (Braunschweig, Germany).

Mycobacterial culture. All strains were stored at -80°C in ball-bearing cryotubes (Mast cryobank®). *M. avium* ATCC 700898, *M. xenopi* DSM43995, *M. abscessus* DSM44196, *M. marinum* DSM44344 and *M. kansasii* DSM 44162 were inoculated onto 7H10 BD BBL™ Stacker™ agar plates purchased from Becton Dickinson GmbH (Heidelberg, Germany), and incubated at 30–42 °C for several days, depending on the strain.

Determination of MIC. A 0.5 McFarland inoculum was prepared for each strain in sterile water and then diluted 1:200 in CAMHB medium (for fast-growing strains) or in CAMHB supplemented with 5% OADC (for slow-growing strains). Each antibiotic was dissolved in dimethyl sulfoxide (DMSO), H_2O or 1N HCl according to the supplier's recommendations. In 96-well plates (VWR International, Pennsylvania, USA), antibiotics were tested over a range of seven concentrations: 2, 4, 8, 16, 32, 64 and 128 µg/mL. Each compound was tested in both technical and biological duplicates. Plates were incubated at 30 °C for five days for *M. abscessus* and for seven days for *M. marinum*, at 37 °C for fourteen days for *M. avium* and *M. kansasii*, and at 42 °C for at least three weeks for *M. xenopi*. MIC values were determined according to CLSI guidelines M27-A2 and the recommendations of the Antibiogram Committee of the French Society of Microbiology (CASFM).

S8. *In vitro* cytotoxicity.

Materials. Dulbecco's Modified Eagle Medium (DMEM) was purchased from Gibco (Life Technologies), trypsin-EDTA solutions (0.25%) from Sigma-Aldrich, and 3-(4,5-dimethylthiazol-2-yl)-2,5-diphenyltetrazolium bromide (MTT) from Invitrogen (Thermo Fisher Scientific).

Cell culture, counting and adhesion. The human hepatocarcinoma cell line HepG2 (from ECACC, Merck) were cultured in DMEM supplemented with 10% foetal bovine serum (FBS). At confluence, cells were washed with trypsin to neutralise the FBS and dissociated using PBS. Cells were counted using a KOVA™ Glasstic™ slide at a concentration of up to 250,000 cells/mL. In 96-well flat-bottomed plates, 25,000 HepG2 cells/mL were added with 100 µL of DMEM. Plates were incubated for 24 hours at 37 °C in a humidified atmosphere containing 5% CO_2 to allow cell adhesion.

Preparation of compounds. Stock solutions of antibiotics were prepared in DMSO. Reference antibiotics were diluted to 10 mM in DMEM supplemented with 10% of FBS. DMSO in DMEM was used as a negative control.

Assessment of cytotoxicity. Each antibiotic was tested at eight concentrations: 0.78; 1.56; 3.12; 6.25; 12.5; 25; 50 and 100 µM. Each compound was performed in triplicate. Plates were incubated for 72 hours at 37 °C in a humidified atmosphere containing 5% of CO_2.

MTT reduction assay. MTT was dissolved in PBS (0.5 mg/mL). A volume of 100 µL of the solution was added to each well, followed by incubation for two hours at 37 °C in a humidified atmosphere containing 5% of CO_2. The medium was removed, and intracellular formazan crystals were solubilized in DMSO and incubated for ten minutes at 37 °C. Cell viability was determined by measuring absorbance at 570 nm, with a reference wavelength of 630 nm, using the Tecan Sunrise™. CC_{50} values were calculated using a non-linear regression fit equation ([inhibition] vs. response, variable slope) available at: https://www.aatbio.com/tools/ic50-calculator. All data are expressed as the mean ± standard deviation of three replicates.

S9. Checkerboard assays.

Preparation of compounds. Hit (*R*)-**4c** was diluted in DMSO to a concentration of 20 mg/mL. EMB.2(HCl) was diluted in sterile water to 10 mg/mL. CLR and RIF were diluted in DMSO to 0.64 mg/mL.

Checkerboard assay. An inoculum of *M. avium* ATCC 700898 at 0.5 McFarland was prepared in sterile water and then diluted 1:100 in CAMHB medium or 7H9 medium supplemented with 5% of OADC. In a 96-well plate, 100 µL of diluted inoculum and 50 µL of each dilution series were added to columns 1 to 8 and rows A to H. Thus, antibiotic stock solutions were prepared at four times the highest final concentration in the wells. Tested concentrations ranged from 8 times the MIC to 0.125 times the MIC of each antibiotic alone. A positive control was added for each experiment. Each compound was tested in technical triplicates. Plates were incubated at 37 °C for fourteen days. All data represent the mean of technical triplicates. MIC determination followed CLSI M27-A2 and CASFM guidelines. Results were expressed as the fractional inhibitory concentration index (FICI), calculated according to the following formula: FICI = FICI (A) + FICI (B) = (MIC of antibiotic A in combination)/(MIC of antibiotic A alone) + (MIC of antibiotic B in combination)/(MIC of antibiotic B alone).
